# A platform for on-the-complex annulation reactions with transient aryne intermediates

**DOI:** 10.1038/s41467-021-23970-8

**Published:** 2021-06-17

**Authors:** Jason V. Chari, Katie A. Spence, Robert B. Susick, Neil K. Garg

**Affiliations:** Department of Chemistry and Biochemistry, University of California, Los Angeles, CA USA

**Keywords:** Synthetic chemistry methodology, Synthetic chemistry methodology

## Abstract

Organometallic complexes are ubiquitous in chemistry and biology. Whereas their preparation has historically relied on ligand synthesis followed by coordination to metal centers, the ability to efficiently diversify their structures remains a synthetic challenge. A promising yet underdeveloped strategy involves the direct manipulation of ligands that are already bound to a metal center, also known as chemistry-on-the-complex. Herein, we introduce a versatile platform for on-the-complex annulation reactions using transient aryne intermediates. In one variant, organometallic complexes undergo transition metal-catalyzed annulations with in situ generated arynes to form up to six new carbon–carbon bonds. In the other variant, an organometallic complex bearing a free aryne is generated and intercepted in cycloaddition reactions to access unique scaffolds. Our studies, centered around privileged polypyridyl metal complexes, provide an effective strategy to annulate organometallic complexes and access complex metal–ligand scaffolds, while furthering the synthetic utility of strained intermediates in chemical synthesis.

## Abstract (web summary)

Organometallic complexes have numerous applications in chemistry and biology, but diversification of their structures is synthetically challenging. Here, the authors report a versatile platform for on-the-complex annulation reactions using transient aryne intermediates to access pi-extended polypyridyl complexes.

## Introduction

Organometallic complexes are prevalent in chemistry and biology, with applications ranging from usage as highly selective catalysts^1^ to therapeutics^2^ and enzyme cofactors^3^. Key to this versatility is the ability to tune function through manipulation of ligand structure. Fine tuning of the ligand sphere can lead to profound changes in the properties of an organometallic complex, including stereoelectronic and photophysical properties, catalyst turnover rate and stability^4^. Thus, the continued growth of organometallic chemistry is contingent on the capacity to access metal–ligand architectures with increased structural diversity and complexity.

**Fig. 1:**
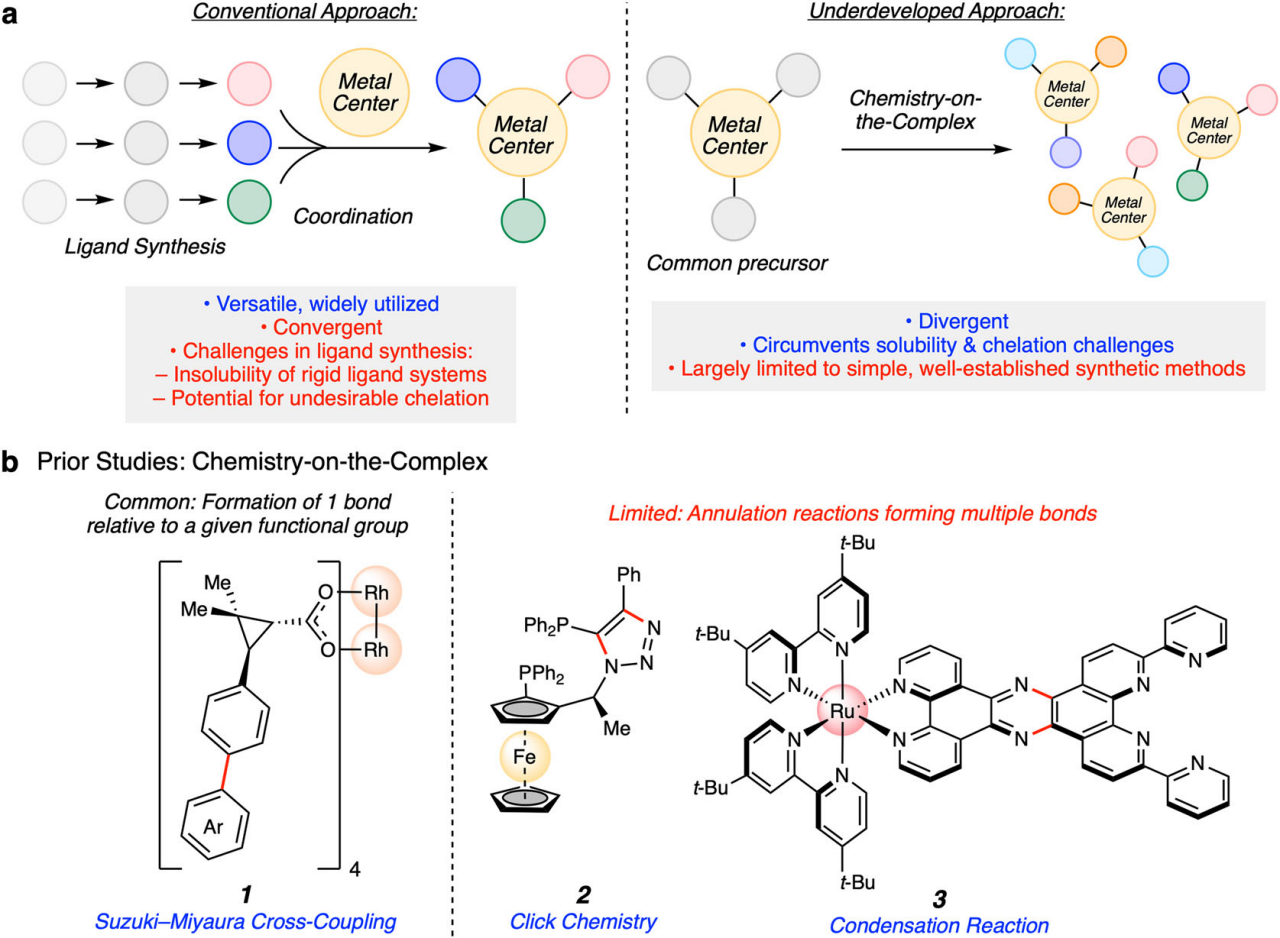
General synthetic approaches toward metal–ligand complexes. **a** Comparison of coordination chemistry and chemistry-on-the-complex. **b** Prior studies involving chemistry-on-the-complex. Me, methyl; Ph, phenyl; *t*-Bu, *tert*-butyl.

Conventional synthetic approaches toward organometallic complexes involve reliance on ligand synthesis followed by coordination to a metal center (Fig. 1a). This general approach remains modular and adaptable, accounting for the syntheses of the majority of known metal–ligand complexes. Nonetheless, this general strategy can have drawbacks in the syntheses of notable ligand classes. For example, the synthesis of strongly chelating ligands can be challenging due to their propensity to form stable metal–ligand chelates and, in turn, prevent the use of metal-mediated transformations such as cross-couplings, C–H functionalization, and annulation reactions^5^. In addition, highly rigid ligand systems can also have poor solubility in organic solvents, thus complicating their syntheses and subsequent coordination to metal centers^6,7^. Finally, ligand synthesis may require long, linear reaction sequences, which can render the process of synthesizing large libraries of organometallic derivatives cumbersome or impractical.

Divergent synthetic routes to organometallic complexes, analogous to those which have proven valuable in medicinal chemistry, are important for generating structurally diverse libraries of compounds. Toward this end, a nascent synthetic approach that complements traditional coordination chemistry is chemistry-on-the-complex^8^, whereby ligands are modified after being bound to a metal center. This strategy provides an attractive means for rapid structural diversification of metal–ligand complexes and can serve to circumvent the aforementioned challenges often encountered in ligand synthesis.

Chemistry-on-the-complex has proven effective in the synthesis of heterodimetallic complexes^9^, with applications in artificial photosynthesis^10^, along with the synthesis and elaboration of ferrocenyl^11,12^ and porphyrin^13^ structures. These studies demonstrate the value of on-the-complex approaches in diversity-oriented synthesis, but also expose the need for further reaction development in this area. One illustrative example of chemistry-on-the-complex is highlighted in Fig. 1b, where Davies and co-workers strategically utilized Suzuki–Miyaura cross-coupling reactions of pre-coordinated dimeric rhodium complexes toward the discovery of catalysts **1** used for the functionalization of unactivated C–H bonds^14^. This case demonstrates the value of the general design, but also highlights that chemistry-on-the-complex is most often used to introduce one bond relative to a given functional group. Methods that allow for the formation of more than one bond using chemistry-on-the-complex remain more limited. Examples include azide cycloadditions (click chemistry) and well-established condensation reactions, resulting in products such as **2**^15^ and **3**^16^, respectively.

**Fig. 2:**
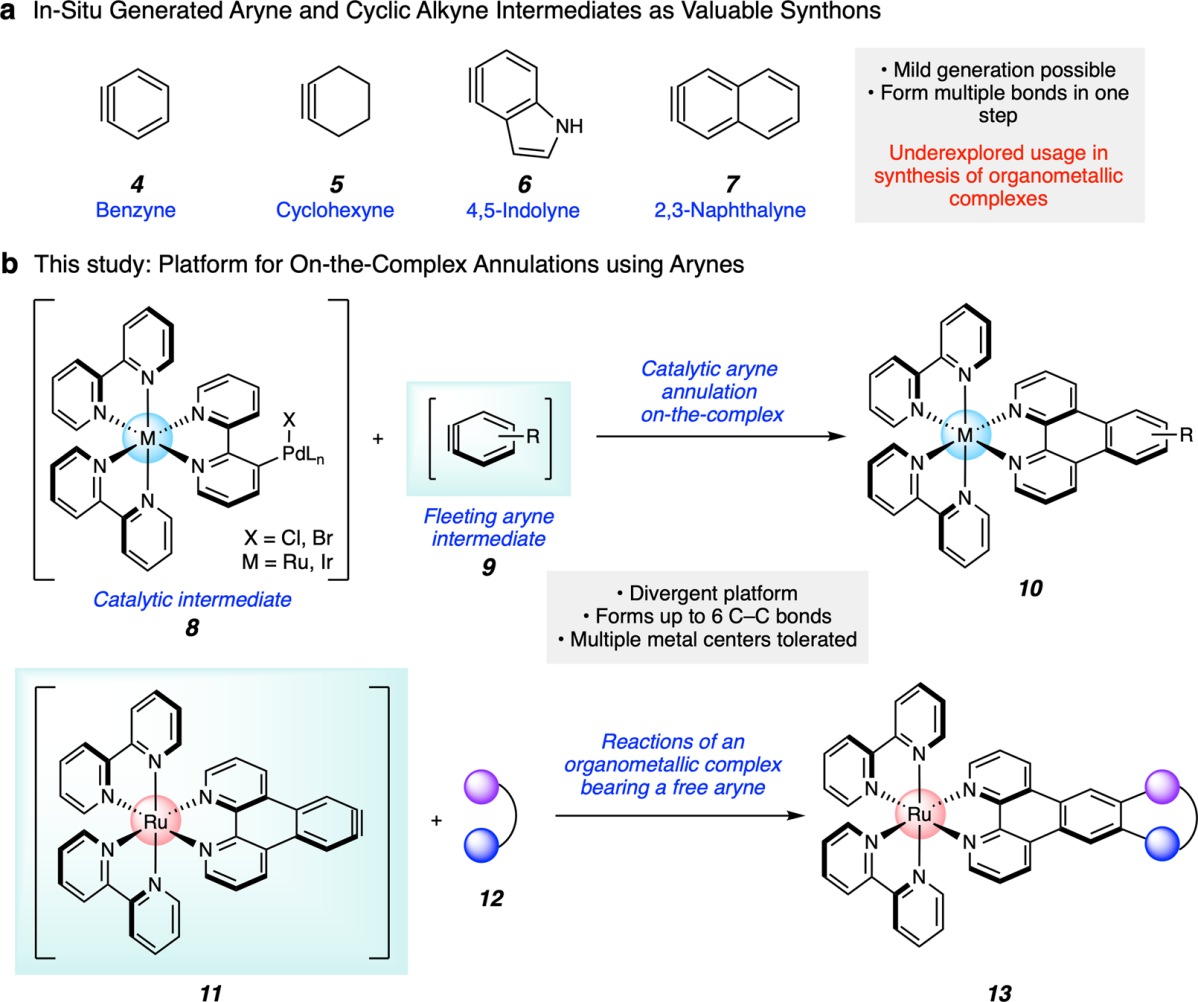
Arynes-on-the-complex approach to metal complexes. **a** Aryne and cyclic alkyne intermediates. **b** Our approach to the direct manipulation of polypyridyl metal complexes using arynes.

In considering the strategic generation of new ring systems on-the-complex, transient aryne intermediates provide a compelling entryway (Fig. 2). Although arynes and related species were once avoided due to their high reactivity, they have recently gained popularity in a number of applications as useful synthons for building molecular complexity^17–26^. For example, strained cyclic intermediates such as **4**–**7** (Fig. 2a) have been used to access heterocycles of value to medicinal chemistry^27^, widely used phosphine ligands^28,29^, agrochemicals^30^, and natural products^31^. Nonetheless, the usage of transient aryne intermediates in chemistry-on-the-complex approaches has remained limited, with only two reports in the literature to date^32,33^. Both examples demonstrate the feasibility of aryne Diels–Alder trappings, but require that the organometallic complex bear a reactive diene ligand. A promising avenue for aryne chemistry-on-the-complex lies in the modification of polypyridyl metal complexes, whose applications span various chemical, biological, and therapeutic disciplines^34–43^.

Herein, we show that intercepting arynes on-the-complex provides a versatile platform for the strategic manipulation of organometallic compounds (Fig. 2b). We disclose two variants. In the first, readily available aryl halides are embedded in the ligand framework and enable palladium-catalyzed annulations with in situ generated arynes. This proceeds via the reaction of catalytically generated bis(metallic) species **8** and fleeting aryne intermediates **9** to give annulated products **10**. In the other variant, compound **11**, a unique organometallic complex that bears an unligated aryne, is generated transiently. In situ trapping with cycloaddition partners **12** gives cycloadducts **13**. Our approaches enable the formation of multiple carbon–carbon (C–C) bonds in a single operation, offer a means to access functionalized polypyridyl metal complexes, underscore the utility of traditionally avoided aryne intermediates, and validate the aryne on-the-complex approach for accessing a diverse range of organometallic compounds.

## Results

### Development of the Pd-catalyzed on-the-complex aryne reaction

**Fig. 3:**
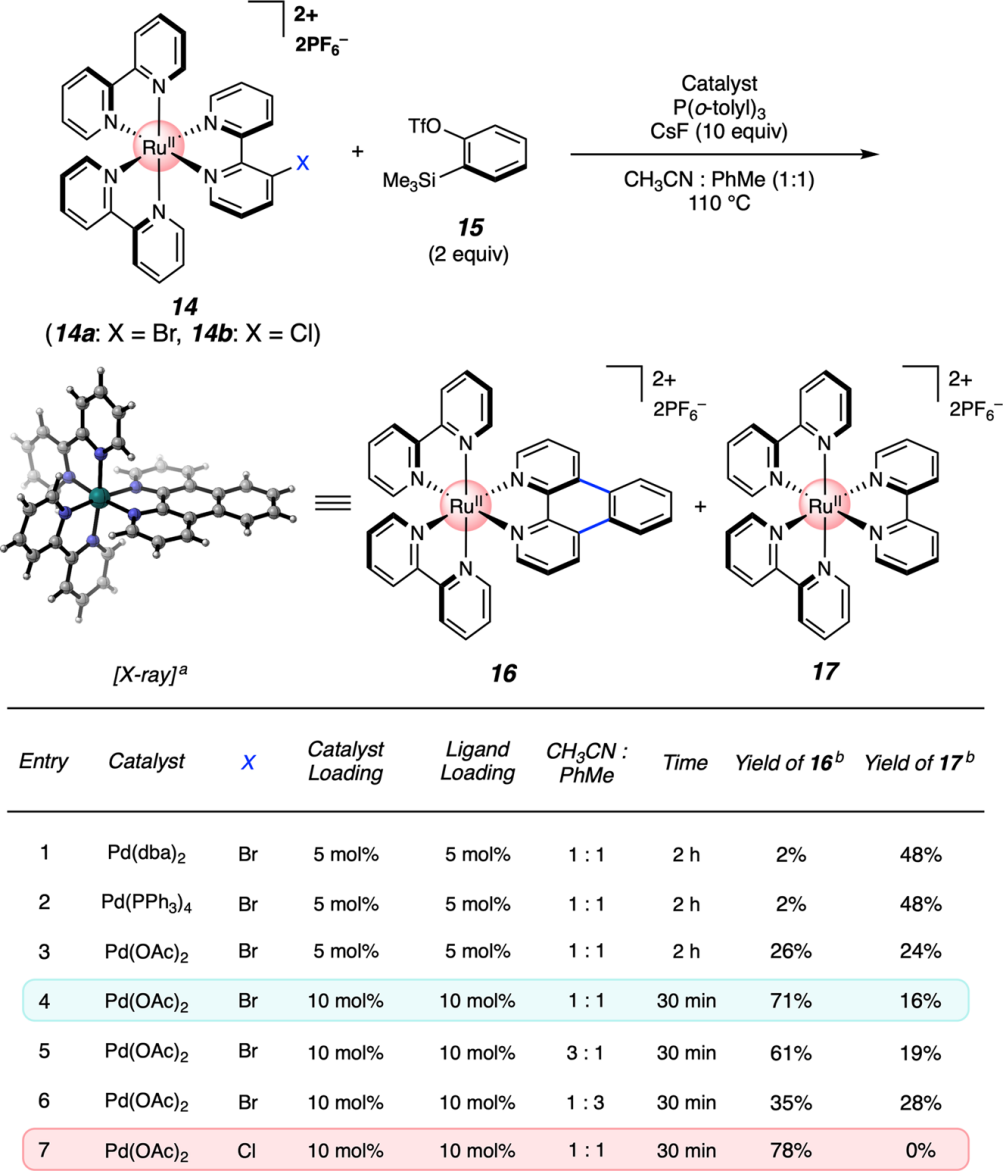
Optimization studies for Pd-catalyzed annulation of benzyne onto Ru-polypyridyl complex 14. ^a^PF_6_ counterions have been removed from the X-ray crystal structure for clarity. ^b^Yields were determined by ^1^H NMR analysis, using 1,3,5-trimethoxybenzene as an external standard. OTf, trifluoromethanesulfonate.

To initiate our studies, we sought to identify a versatile functional group handle for aryne on-the-complex manipulations. We settled on the use of aryl halides, given their ready availability and their prevalence in transition metal-catalyzed reactions, and prepared halogenated Ru(bpy)_3_ derivatives **14** (Fig. 3 and see Supplementary Information). Although many impressive examples of Pd-catalyzed transformations of arynes have now been reported^44–51^ use of this chemistry in the manipulation of organometallic complexes has remained unexplored. Inspired by Larock’s impressive annulation of biaryl halides^52,53^, we sought to perform a Pd-catalyzed annulation of **14** with commercially available benzyne precursor **15**. Initial attempts involved the use of Pd(dba)_2_ and P(*o*-tolyl)_3_, in the presence of CsF, but were met with limited success, as we observed formation of the desired π-extended adduct **16**, albeit in only 2% yield (entry 1). Instead, undesired protodehalogenation product, Ru(bpy)_3_ (**17**), was observed in 48% yield. Efforts to prevent this dehalogenation pathway via reduced temperatures and rigorous exclusion of air and moisture proved unfruitful^54^, as did the use of other Pd^0^ sources such as Pd(PPh_3_)_4_ (e.g., entry 2). Alternatively, the use of Pd(OAc)_2_ led to an improved 26% yield of the desired π-extended adduct **16** (entry 3). By increasing the catalyst and ligand loadings to 10 mol%, we observed a further increase in yield of **16** to 71%, with a reaction time of just 30 min (entry 4). Employing modified ratios of the co-solvents, acetonitrile and toluene, resulted in decreased reaction efficiency (entries 5 and 6) (CsF, which governs aryne formation, has sparing solubility in organic solvents. By modulating the solvent mixture, one can tune the effective concentration of aryne in solution. In a catalytic reaction, such as that reported herein, it is critical to balance the amount of aryne in solution relative to the reactive organometallic species.). Finally, shifting from brominated substrate **14a** to chlorinated derivative **14b** effectively shut down the dehalogenation pathway and provided the desired product in 78% yield (entry 7). We surmise that the conversion of **14** + **15** to **16** proceeds via initial oxidative addition and aryne formation occurring concomitantly (see Figs. 2b, 8 and 9), followed by aryne insertion, palladation, and reductive elimination (For a pertinent report and proposed mechanism for Pd-catalyzed annulation of arynes with halobiaryl substrates, see ref. ^55^.). It is worth noting that attempts to perform the analogous annulation on uncoordinated bromo- or chlorobipyridine ligands proved unproductive, potentially owing to *N*,*N*-chelation of palladium (see Supplementary Information), thus highlighting an aforementioned benefit of on-the-complex chemistry.

### Scope of the Pd-catalyzed annulation

**Fig. 4:**
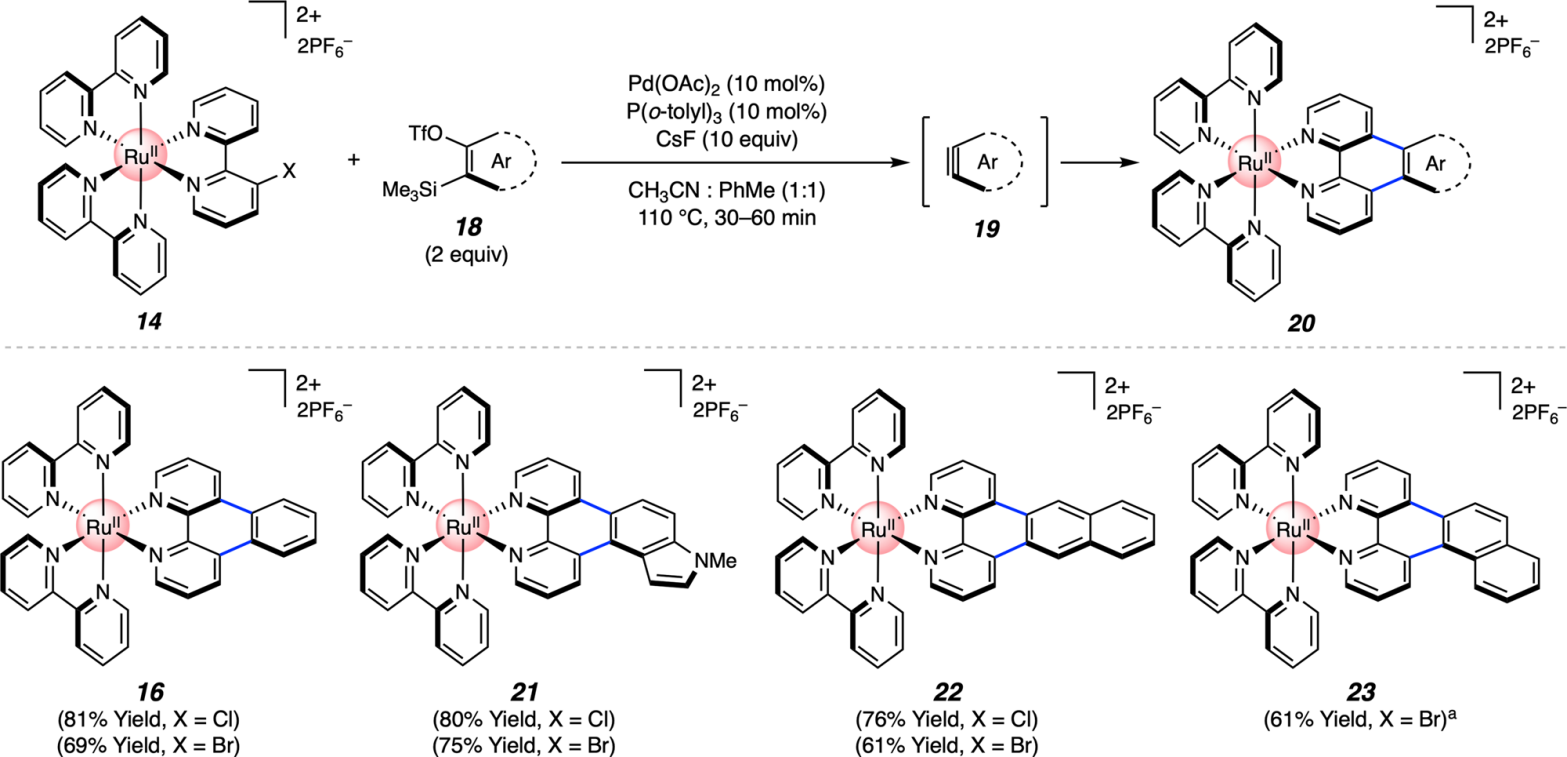
Aryne scope of the Pd-catalyzed aryne annulation. Yields shown reflect the average of two isolation experiments. ^a^Significant decomposition was observed when *X* = Cl. Me, methyl; OTf, trifluoromethanesulfonate.

Variation of either the aryne or organometallic component was tolerated in the annulation, thus giving rise to a range of polypyridyl metal complexes in synthetically useful yields. With regard to the aryne component (Fig. 4), benzyne adduct **16** was isolated in 81% yield (*X* = Cl) or 69% yield (*X* = Br) using standard column chromatography. Notably, the only available protocol to access the diazatriphenylene ligand found in **16** involves the use of hazardous reagents and exceptionally forcing conditions^56^. *N*-Me-4,5-indolyne could also be employed to deliver adduct **21** in 80% yield (*X* = Cl) or 75% yield (*X* = Br), thus demonstrating the expedient incorporation of a heterocycle into the π-framework of the metal complex. Naphthalynes were also deemed competent reaction partners, as judged by the formation of **22** and **23**. Prior routes to synthesize the naphthophenanthroline ligand present in **22** are lengthy or low yielding^57^, in part due to poor solubility of the free ligand^6^.

**Fig. 5:**
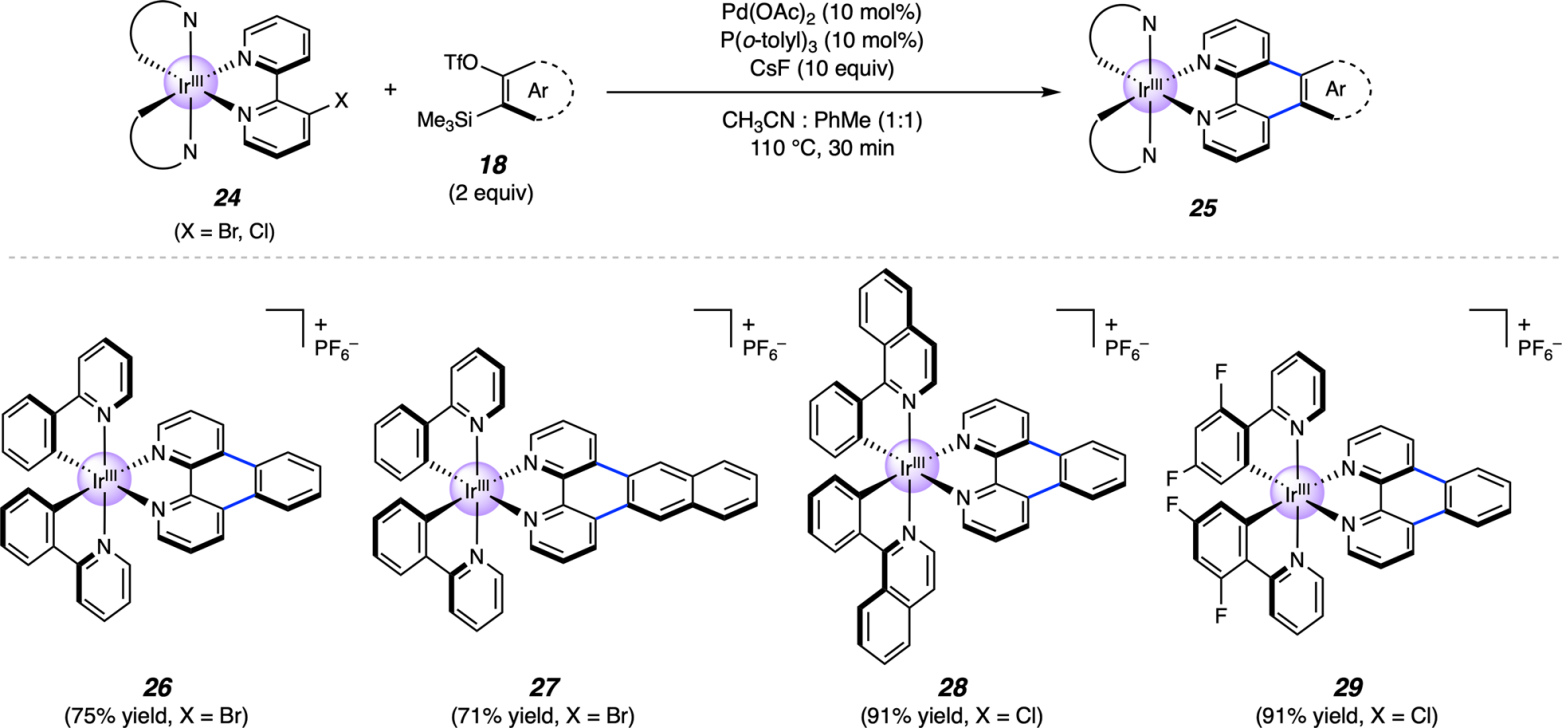
Pd-catalyzed aryne annulation of Ir-centered polypyridyl metal complexes. Yields shown reflect the average of two isolation experiments. OTf, trifluoromethanesulfonate.

Although we primarily focused the current study on Ru complexes, we opted to probe the methodology in the context of Ir-centered polypyridyl complexes as well. Ir-centered polypyridyl complexes are prevalent in photochemistry^58–61^, with documented value of extended π-conjugation in structure–property relationship studies^62^. We were gratified to find that the methodology could be used to access several Ir(ppy)_2_bpy derivatives, as delineated in Fig. 5. Via the intermediacy of benzyne and 2,3-naphthalyne, **26** and **27** could be accessed in 75% and 71% yield, respectively. Excellent yields were also observed upon varying the phenylpyridine ligands of the substrate, as isoquinolinyl annulation product **28** and tetrafluorinated adduct **29** could each be obtained in high yields from the corresponding chloride substrates.

**Fig. 6:**
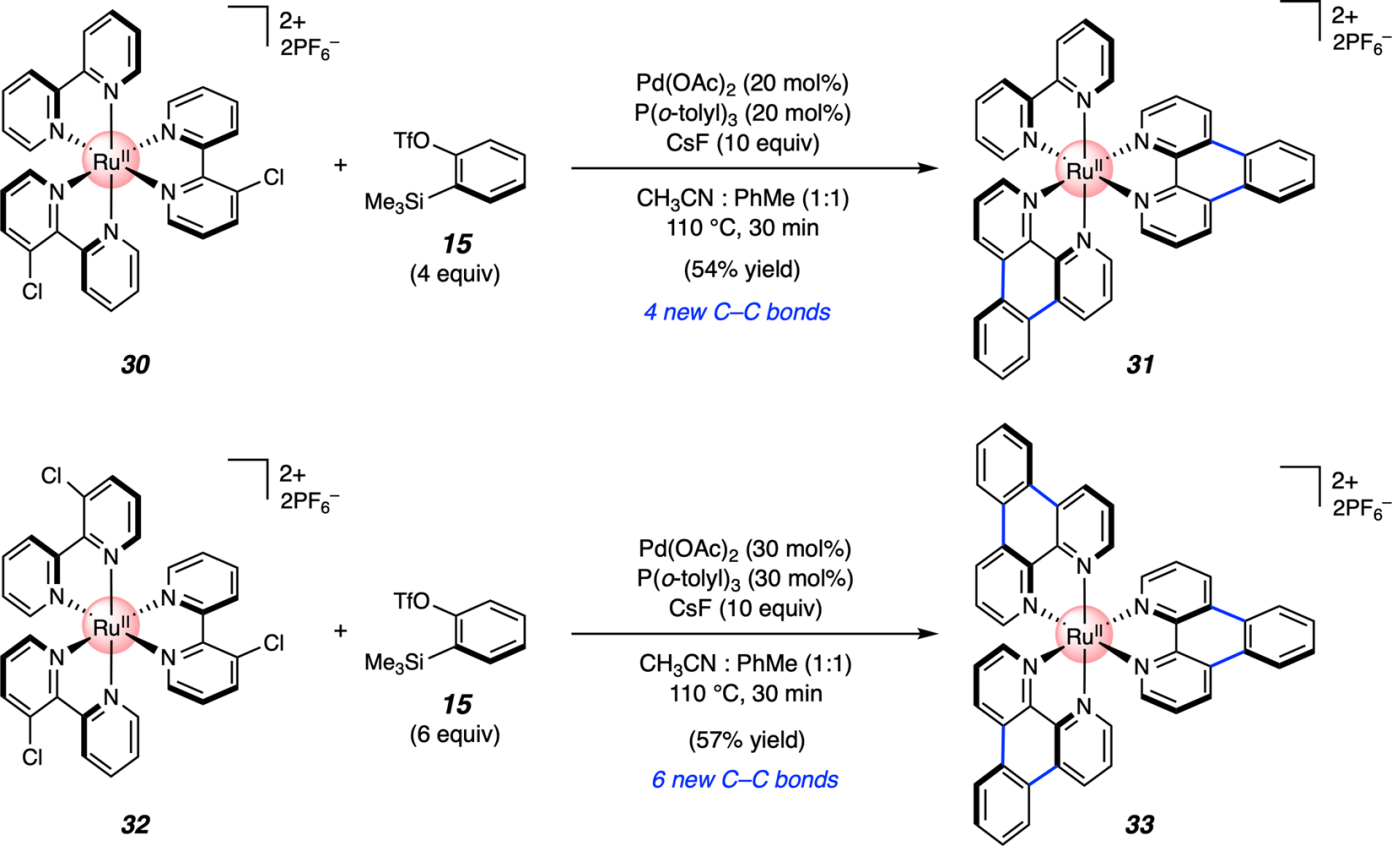
Pd-catalyzed aryne annulation at multiple sites of Ru complexes. Yields shown reflect the average of two isolation experiments. OTf, trifluoromethanesulfonate.

To assess the possibility of carrying out multiple annulations on a given organometallic complex and test the limits of our aryne on-the-complex chemistry, we prepared Ru complexes **30** and **32**, bearing two or three chlorides, respectively (Fig. 6). Subjecting these complexes independently to slightly modified reaction conditions delivered double and triple annulation products **31** and **33**, via the efficient formation of four or six new C–C bonds, respectively. Half of the bonds formed in either process arise from arene C–H functionalization.

### Generation and trapping of an organometallic aryne

**Fig. 7:**
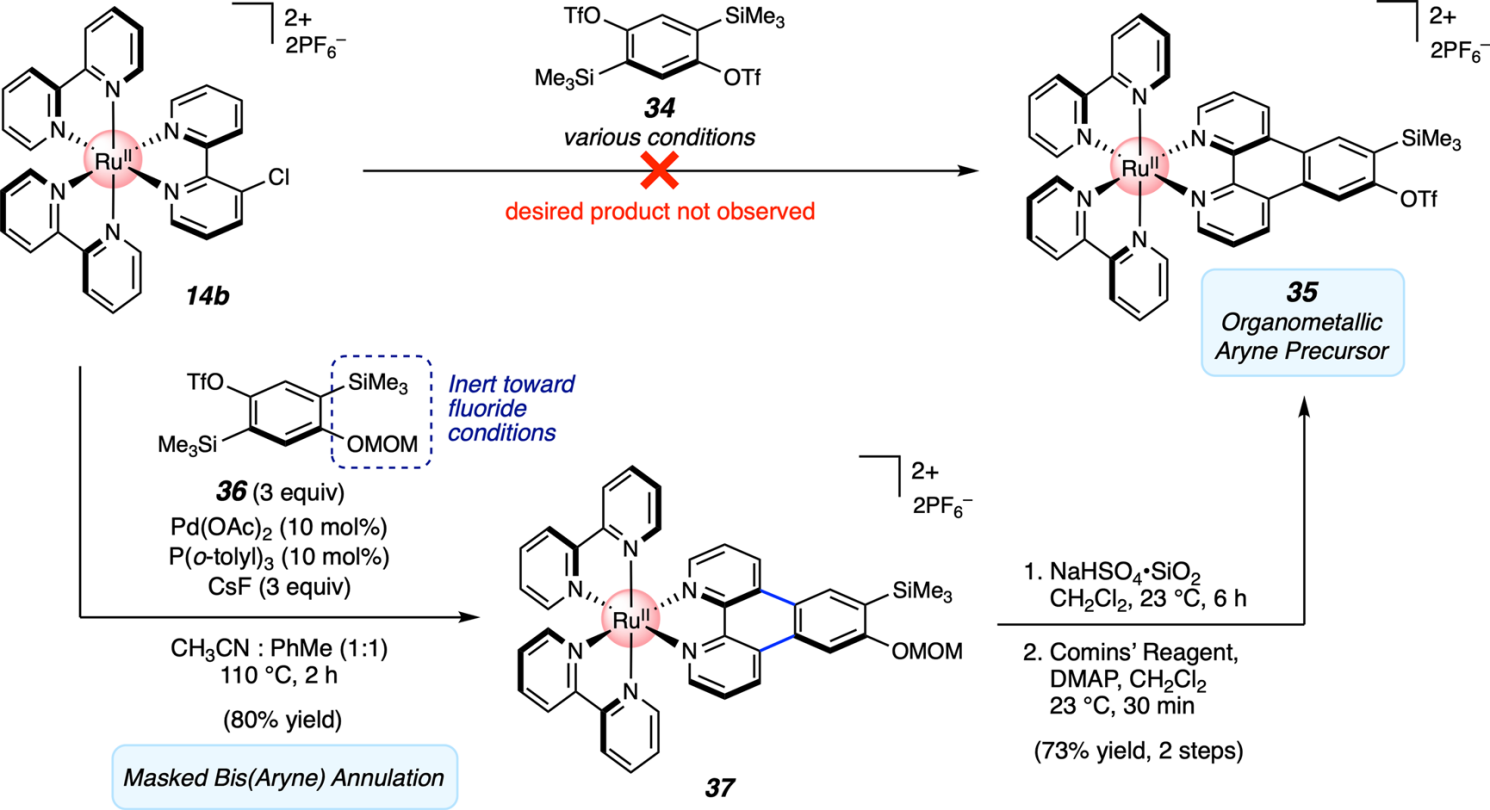
Synthesis of an organometallic aryne precursor via masked bis(aryne) annulation. OTf, trifluoromethanesulfonate; MOM, methoxymethyl; DMAP, 4-dimethylaminopyridine.

With an aryne-driven method for the annulation of organometallic complexes in hand, we sought to exploit this methodology to further extend the utility of aryne chemistry in accessing organometallic complexes. In particular, we sought to generate a free aryne on the organometallic complex itself and trap it in cycloaddition reactions. In contrast to arynes coordinated directly to metal centers (e.g., Zr, Ti)^55^, which are well-studied, free arynes embedded in an organometallic framework have remained elusive. Notably, previous efforts toward organometallic species bearing a free aryne have been met with difficulty^63^, and we therefore viewed the development of strategies in this area as an opportunity for advances in both aryne chemistry and chemistry-on-the-complex. As shown in Fig. 7, we targeted silyl triflate **35** as the suitable aryne precursor. Unfortunately, initial efforts to access **35** via the annulation of Ru-complex **14b** with bis(silyl triflate) **34**^64^ proved unsuccessful. As a workaround, we employed methoxymethyl (MOM) ether **36**, prepared in two steps from commercially available materials, in the annulation reaction. After careful tuning of reaction conditions, adduct **37** could be generated in 80% yield with retention of both the MOM ether and trimethylsilyl group^65^. Subsequent cleavage of the MOM group, followed by triflation, delivered the desired silyl triflate **35** in 73% yield over two steps.

**Fig. 8:**
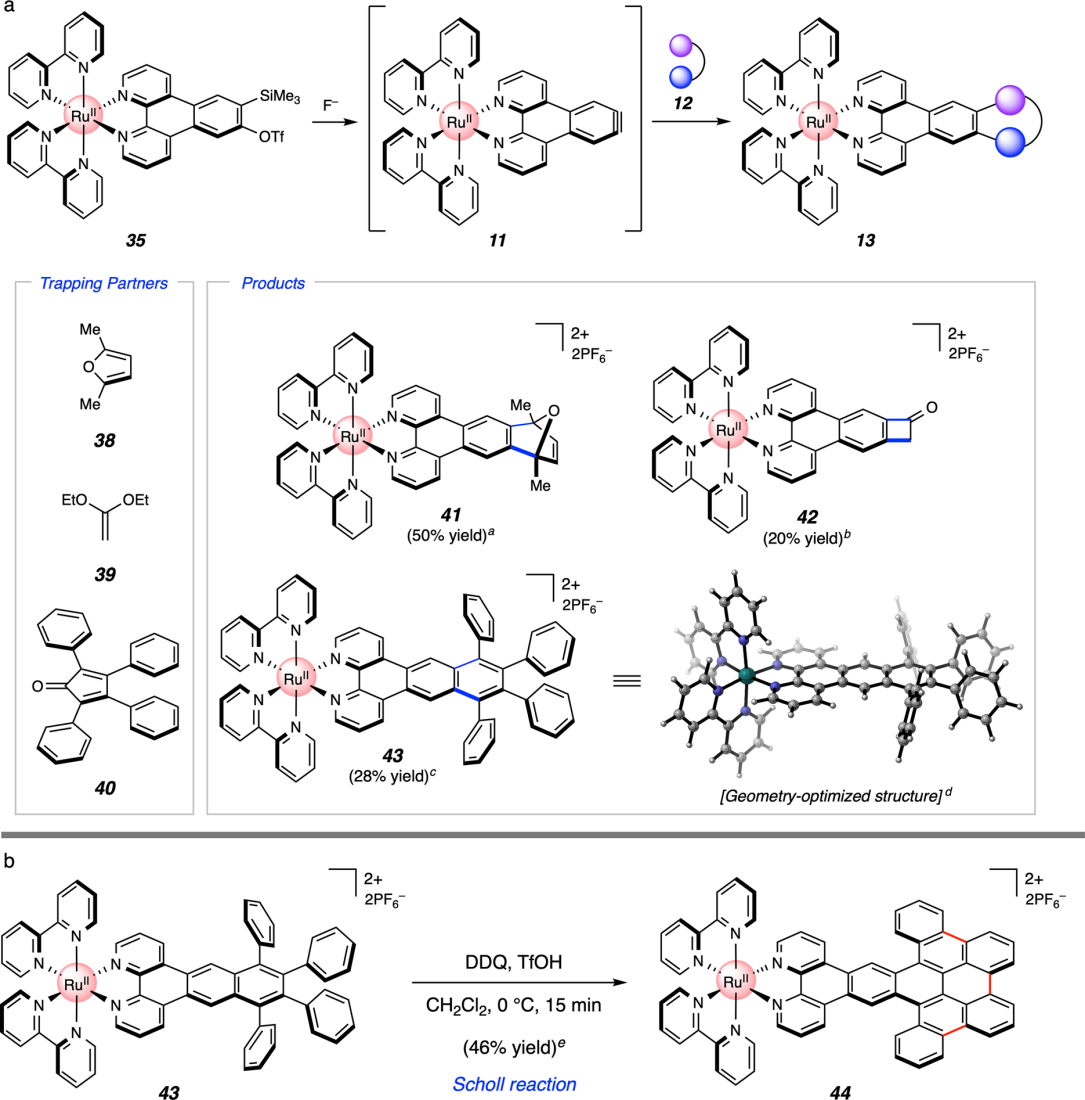
Mild generation and trapping of a Ru(II) aryne. **a** Cycloaddition reactions of organometallic aryne **11**. **b** Scholl reaction of **43** to give **44**. ^a^Conditions: **35** (1 equiv), **38** (10 equiv), CsF (5 equiv), CH_3_CN, 23 °C, 12 h. ^b^Conditions: **35** (1 equiv), **39** (5 equiv), CsF (3 equiv), CH_3_CN, 23 °C, 1.5 h; TFA. ^c^Conditions: **35** (1 equiv), **40** (2 equiv), CsF (5 equiv), CH_3_CN:CH_2_Cl_2_ (2:1), 50 °C, 1.5 h. ^d^Geometry optimization of **43** (without counterions) was performed using B3LYP/6-31 G(d)/LANL2DZ/CPCM(MeCN). ^e^Conditions: **43** (1 equiv), DDQ (20 equiv), CH_2_Cl_2_:TfOH (40:1), 0 °C, 15 min. OTf, trifluoromethanesulfonate; Me, methyl; Et, ethyl; DDQ, 2,3-dichloro-5,6-dicyano-1,4-benzoquinone.

As highlighted in Fig. 8a, we found that silyl triflate **35** indeed served as a suitable precursor to aryne **11**, which, in turn, underwent cycloaddition in situ with trapping partners **12**. Trapping of **11** in the presence of 2,5-dimethylfuran (**38**) gave Diels–Alder adduct **41** in 50% yield. In addition, a formal [2 + 2] cycloaddition of **11** with diketene acetal **39** was achieved, generating adduct **42** bearing a carbonyl functional handle. Finally, trapping of **11** with tetraphenylcyclopentadienone (**40**) gave rise to the unusual adduct **43** via a Diels–Alder and subsequent cheletropic cycloreversion to extrude CO. Complex **43** displays an excited state lifetime that is roughly two-fold longer than that of Ru(bpy)_3_ (see Supplementary Information). Overall, the ability to access **41**–**43** from aryne precursor **35** showcases a free aryne being generated directly on an organometallic complex and demonstrates the utility of such species to access metal complexes with a diverse array of ring systems. Moreover, the results shown in Figs. 7 and 8a provide an unconventional strategy to access unique coordination complexes via two iterations of aryne on-the-complex chemistry (i.e., **14b** → **37** and **35** → **41**–**43**), which collectively enables the formation of four C–C bonds in each organometallic complex made.

Lastly, we explored the possibility of further manipulating the interesting organometallic complex **43**. Geometry optimization of **43** via DFT calculations suggests that its four phenyl substituents are oriented perpendicular to the plane of the bipyridyl ligand (see Fig. 8a and Supplementary Information). We therefore questioned whether these rings could be joined through an oxidative cyclization reaction (Fig. 8b). Gratifyingly, treatment of **43** with DDQ and triflic acid facilitated triple C–C bond formation to give **44** in 46% yield, which notably occurs without oxidation of the Ru center. This approach to **44** circumvents solubility challenges historically encountered in efforts to access similar π-extended complexes through off-the-complex protocols^66^, while providing access to a unique scaffold via a Scholl reaction^67^ of a Ru-centered organometallic complex^68^.

### Photophysical studies

**Fig. 9:**
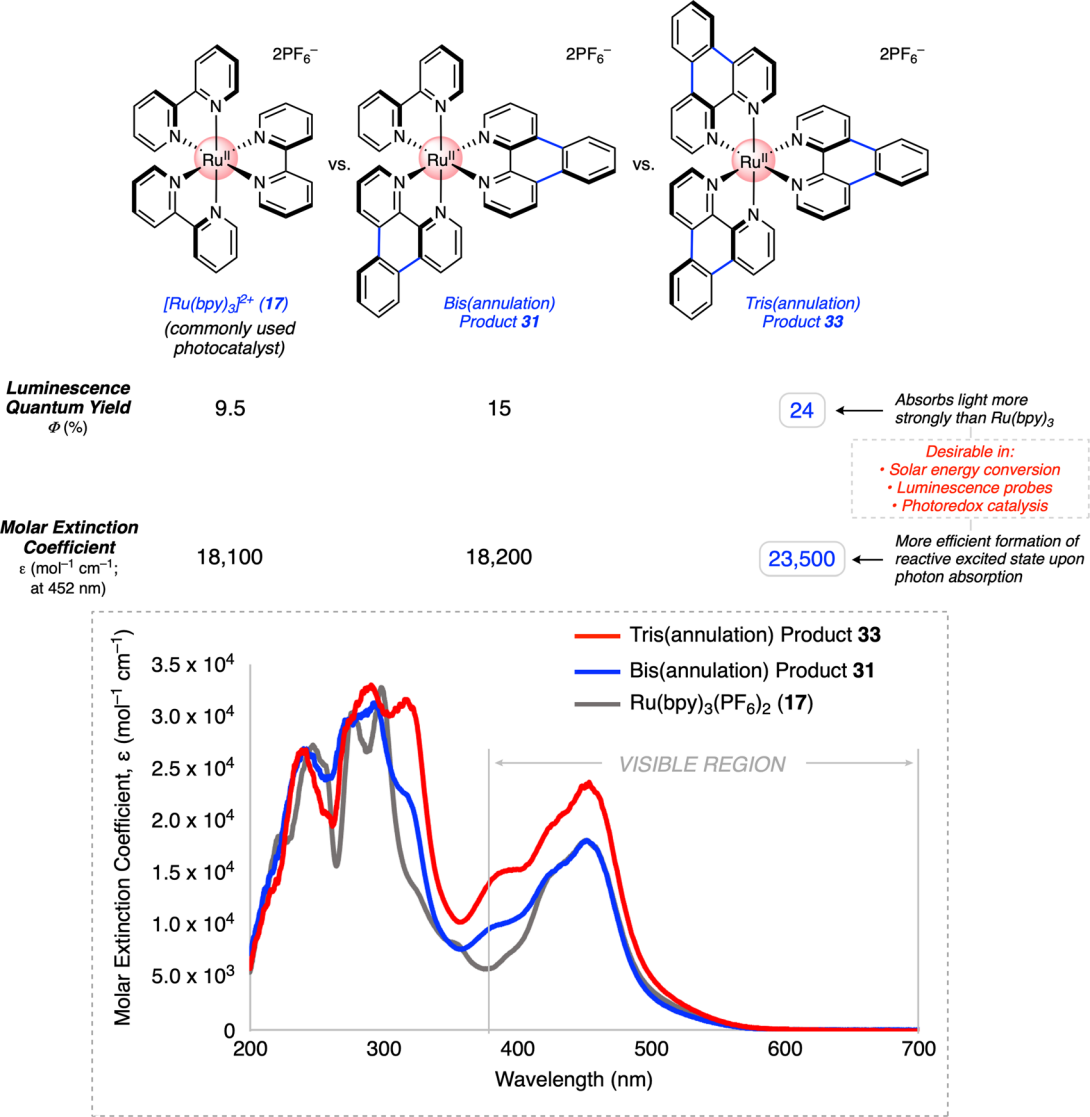
Photophysical studies of bis(annulation) product 31 and tris(annulation) product 33 relative to [Ru(bpy)_3_]^2+^ (17). Evaluation of luminescence quantum yield (Φ, %) and molar extinction coefficient (ε, mol^–1^ cm^–1^). Experimental absorption spectra (molar extinction coefficient) are shown from 200 to 700 nm.

Although our primary objective was to develop the fundamental synthetic methodology described above, we also sought to identify and evaluate trends in photophysical properties of the products obtained. We deemed this particularly important given the broad impact of [Ru(bpy)_3_]^2+^ (**17**) and other polypyridyl metal complexes in light-based applications, as mentioned earlier. Thus, we compared the photophysical properties of [Ru(bpy)_3_]^2+^ (**17**) to that of annulation products obtained through our methodology. Examining luminescence quantum yield and molar extinction coefficients provided useful insights and revealed adducts **31** and **33** as being particularly interesting (Fig. 9). First, a positive trend in luminescence quantum yield was observed from [Ru(bpy)_3_]^2+^ (**17**) to bis(annulation) product **31** to tris(annulation) product **33**. In particular, **33** exhibits a high luminescence quantum yield of 24%, which is notably 2.5-fold greater than that of [Ru(bpy)_3_]^2+^ (**17**) at 9.5%. A high luminescence quantum yield indicates more efficient formation of a reactive excited state upon photon absorption, and is desirable in such applications as luminescence sensing, solar energy conversion, and photoredox catalysis^36^. Additionally, **33** displays a higher molar extinction coefficient across the visible region (e.g., 23,500 mol^–1^ cm^–1^ at 452 nm) than that of [Ru(bpy)_3_]^2+^ (**17**) (e.g., 18,100 mol^–1^ cm^–1^ at 452 nm), which suggests that it exhibits stronger ground state absorption of light in the visible region, a desirable quality in the aforementioned applications. All three compounds exhibit a strong visible absorption peak at 452 nm, which is characteristic of the metal-to-ligand charge transfer (MLCT)-based luminescence that is typically observed in Ru(II) polypyridyl complexes. Of note, a shoulder also emerges in the region from 370 to 410 nm in compounds **31** and, more prominently, in **33**, and can likely be ascribed to delocalized ^1^π–π* transitions that are characteristic of other π-expansive ligands (e.g., phenazine derivatives)^69^. These findings bode well for the future use of our methodology to access complexes with promising and improved photophysical properties.

## Discussion

We have developed two variants of elusive aryne on-the-complex chemistry in the context of privileged polypyridyl metal complexes. In one variant, organometallic complexes bearing aryl halides undergo transition metal-catalyzed annulations with in situ, transiently generated arynes. In the second version, an organometallic complex bearing a free aryne is intercepted in cycloaddition reactions to access complex scaffolds. Multiple C–C bonds (i.e., up to 6) can be formed in single synthetic operations, thus providing access to metal complexes bearing unique substitution patterns. These studies not only underscore the utility of traditionally avoided aryne intermediates and the value of on-the-complex aryne chemistry, but should also stimulate the development of on-the-complex reactions that enable transformations that are challenging by other means. Further studies will aim to evaluate and expand the utility of this methodology in accessing other valuable classes of organometallic complexes. From the standpoint of synthetic strategy, we hope these studies encourage the use of pre-coordinated ligands as synthons in the pursuit of complex organometallic architectures.

## Methods

### General procedure for Pd-catalyzed on-the-complex annulation of arynes

To a flame-dried 1-dram vial was added Pd(OAc)_2_ (6.7 µmol, 10 mol%), substrate **14a** (0.066 mmol, 1.0 equiv), P(*o*-tolyl)_3_ (6.7 µmol, 10 mol%), MeCN (0.5 mL, 0.15 M), PhMe (0.5 mL, 0.15 M), silyl triflate **15** (0.134 mmol, 2.0 equiv), an oven-dried magnetic stirbar, and CsF (101 mg, 0.663 mmol, 10.0 equiv) sequentially. The reaction was then purged with N_2_ for 5 min before being stirred at 110 °C for 30–60 min. After cooling to 23 °C, the mixture was filtered through a plug of celite with MeCN (6 mL), concentrated under reduced pressure, and purified by flash chromatography (100% EtOAc **→** 14:1:1 MeCN:H_2_O:sat. aq. KNO_3_). To the concentrated aqueous mixture was added saturated aqueous KPF_6_ (50 mL) to precipitate the desired product, followed by addition of CH_2_Cl_2_ (50 mL). The layers were separated and the aqueous layer was extracted with CH_2_Cl_2_ (2 × 50 mL). The combined organic layers were then dried over Na_2_SO_4_ and concentrated under reduced pressure to afford adduct **16**.

### General procedure for cycloaddition reactions of Ru-centered aryne

To a flame-dried 1-dram vial was added silyl triflate **35** (16.9 µmol, 1.0 equiv) and dissolved in MeCN (0.8 mL, 0.02 M). 2,5-Dimethylfuran (**38**, 0.17 mmol, 10 equiv) was then added in one portion. While stirring, CsF (0.085 mmol, 5 equiv) was added in one portion and the reaction was stirred at 23 °C for 1 h. After 1 h, the reaction mixture was filtered through a plug of celite with MeCN (10 mL), concentrated under reduced pressure, and purified by flash chromatography (100% EtOAc **→** 14:1:1 MeCN:H_2_O:sat. aq. KNO_3_). To the concentrated aqueous mixture was added saturated aqueous KPF_6_ (50 mL) to precipitate the desired product, followed by addition of CH_2_Cl_2_ (50 mL). The layers were separated and the aqueous layer was extracted with CH_2_Cl_2_ (2 × 50 mL). The combined organic layers were then dried over Na_2_SO_4_ and concentrated under reduced pressure to afford adduct **41**.

## Supplementary information

Supplementary Information

## Data Availability

Crystallographic data are available free of charge from the Cambridge Crystallographic Data Centre under CCDC 2048567. The authors declare that all other data supporting the findings of this study are available within the manuscript and its supplementary information files. Correspondence and requests for materials should be addressed to N.K.G. (neilgarg@chem.ucla.edu).
