# Supplementary material for: A platform for on-the-complex annulation reactions with transient aryne intermediates
Source: Nat Commun. 2021 Jun 17;12:3706. doi: 10.1038/s41467-021-23970-8 (PMC8211856; doi:10.1038/s41467-021-23970-8)
Supplement: Supplementary file 1 — Supplementary Information [file 41467_2021_23970_MOESM1_ESM.pdf]

A platform for on-the-complex annulation reactions with transient aryne intermediates

Jason V. Chari†, Katie A. Spence†, Robert B. Susick, and Neil K. Garg*

Department of Chemistry and Biochemistry, University of California
Los Angeles, California 90095

Supplementary Information – Table of Contents

Supplementary Methods **S2**

Experimental Procedures **S3**
- **A. Synthesis of Halobipyridine Organometallic Complexes** **S3**
- **B. Optimization of on-the-Complex Annulation with Benzyne** **S14**
- **C. Crystallographic Data** **S16**
- **D. Synthesis of Silyl Triflate Aryne Precursors** **S17**
- **E. Scope of Pd-Catalyzed Aryne Annulation** **S20**
- **F. Synthesis and Trapping Experiments of Ru-Centered Aryne** **S28**

Photophysical Data **S35**
- **A. Compiled Photophysical Data Table** **S36**
- **B. UV–Vis Spectra** **S37**
- **C. Molar Extinction Coefficient Measurements** **S38**
- **D. Quantum Yield Measurements** **S41**

Computational Methods **S42**
- **A. Complete Citation of Gaussian 16** **S42**
- **B. Energy and Cartesian Coordinates for Optimized Structure** **S42**

^{1}H NMR Spectra **S46**
^{13}C NMR Spectra **S75**

Supplementary References **S100**

Supplementary Methods

Unless stated otherwise, reactions were conducted in flame-dried glassware under an atmosphere of nitrogen or argon and commercially obtained reagents were used as received. Anhydrous solvents were either freshly distilled or passed through activated alumina columns, unless otherwise stated. Reaction temperatures were controlled using an IKAmag temperature modulator, and unless stated otherwise, reactions were performed at room temperature (approximately 23 °C). Cesium fluoride (CsF), palladium(II) acetate ($Pd(OAc)_2$), and di-μ-chlorotetrakis[2-(2-pyridinyl-kN)phenyl-kC]diiridium(III) (**53**) were obtained from Strem Chemicals. Methyl iodide was obtained from Spectrum Chemical. Ruthenium(III) chloride trihydrate (**51**), 3-(trimethylsilyl)-2-naphthyl trifluoromethanesulfonate (**71**), 2,5-dibromohydroquinone (**63**), 2,5-dimethylfuran (**38**), and 2,3-dichloro-5,6-dicyano-1,4-benzoquinone (DDQ) were obtained from Combi-Blocks. 1-(trimethylsilyl)-2-naphthyl trifluoromethanesulfonate (**72**) was obtained from TCI America. Tri(*o*-tolyl)phosphine (P(*o*-tolyl)$_3$), Garg 4,5-indolyne precursor (**61**), *cis*-bis(2,2'-bipyridine)dichlororuthenium(II) hydrate (**50**), phosphorus trichloride (PCl_3), 1,1,1,3,3,3-hexamethyldisilazane (HMDS), bromomethyl methyl ether (MOMBr), tetraphenylcyclopentadienone (**40**), di-μ-chlorotetrakis[2-(1-isoquinolinyl-N)phenyl-C]diiridium(III) (**55**), and dichlorotetrakis[3,5-difluoro-2-(2-pyridinyl)phenyl]diiridium(III) (**57**) were obtained from Sigma-Aldrich. *N*-bromosuccinimide and *N*-chlorosuccinimide were obtained from Acros Organics. Phosphorus tribromide (PBr_3) and trifluoromethanesulfonic acid (TfOH) was obtained from Oakwood Chemical. 1,1'-Diethoxyethene (**39**) was obtained from Fluka. 2,2'-bipyridine *N*-oxide (**45**) was prepared according to literature procedures[1], and is also commercially available. Thin-layer chromatography (TLC) was conducted with EMD gel 60 F254 pre-coated plates (0.25 mm for analytical chromatography and 0.50 mm for preparative chromatography) and visualized using UV. Silicycle Siliaflash P60 (particle size 0.040–0.063 mm) was used for flash column chromatography. ^{1}H NMR spectra were recorded on Bruker spectrometers (at 400, 500 and 600 MHz) and are reported relative to residual solvent signals. Data for ^{1}H NMR spectra are reported as follows: chemical shift (δ ppm), multiplicity, coupling constant (Hz), integration. Data for ^{13}C NMR are reported in terms of chemical shift (at 100 Hz and 125 MHz). IR spectra were recorded on a Perkin-Elmer UATR Two FT-IR spectrometer and are reported in terms of frequency absorption (cm^{-1}). ESI-TOF measurements were carried out in positive ionization mode on a Waters LCT-Premier XE

Time of Flight Instrument controlled by MassLynx 4.1 software (Waters Corporation, Milford MA). The instrument was equipped with the Multi Mode Ionization source operated in the electrospray mode. A solution of Leucine Enkephalin (Sigma Chemical, L9133) was used in the Lock-Spray to obtain accurate mass measurements. Samples were infused using direct loop injection on a Waters Acquity UPLC system. GC-MS measurements were carried out using an Agilent Model 7693 Autosampler, 7890B Gas Chromatograph, and 7250 Q-TOF Mass Selective Detector in the Electron Ionization mode. Sample injection was carried out in split mode with inlet temperature set to 280 °C. Separation was carried out on an Agilent HP5-MS column with dimensions 30m x 250 µm x 0.25 µm. Ultra High Purity Grade He (Airgas) was used as carrier gas with the flow set to 1.1 mL/min in constant flow mode. The initial oven temperature was set to 70 °C for 1 min followed by a 20 °C/min ramp to a final temperature of 300 °C which was maintained for 4 min. A 3.0 min solvent delay was used. EI energy was set to 70 eV. The MSD was set to scan the 50–500 m/z range. Data collection and analysis were performed using Mass Hunter Acquisition and Qualitative Analysis software (Agilent).

Experimental Procedures

A. Synthesis of Halobipyridine Organometallic Complexes

Note: The following procedures for the synthesis of halobipyridines were adapted from a published literature protocol[2] to facilitate gram-scale synthesis.

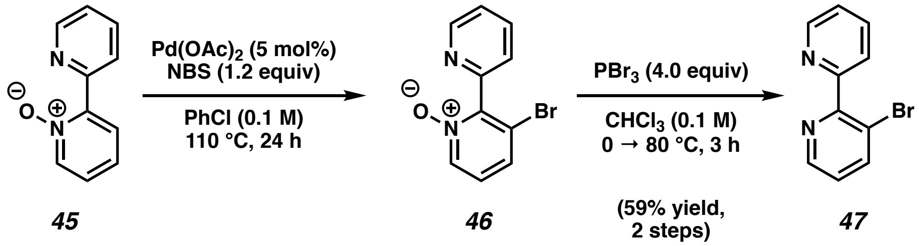

Bromobipyridine 47. To a flask containing 2,2'-bipyridyl *N*-oxide (**45**, 1.00 g, 5.81 mmol), $Pd(OAc)_2$ (65.4 mg, 0.291 mmol, 5 mol%), and *N*-Bromosuccinimide (1.24 g, 6.97 mmol, 1.2 equiv) was added PhCl (58 mL, 0.10 M). This suspension was then heated to 110 °C and stirred for 24 h. The reaction was then allowed to cool to 23 °C, at which point it was transferred with

CH_2Cl_2 (30 mL) to a separatory funnel containing 1.0 M saturated aqueous NaOH (30 mL). The layers were separated and the aqueous layer was extracted with CH_2Cl_2 (3 x 30 mL). The combined organic layers were then dried over Na_2SO_4, filtered, and concentrated under reduced pressure to afford the corresponding bromobipyridine *N*-oxide **46** as a brown oil. This was carried forward without further purification.

The crude oil was dissolved in $CHCl_3$ (50 mL, 0.10 M) and cooled to 0 °C. To this stirred solution was added PBr_3 (2.0 mL, 21.0 mmol, 4.0 equiv) dropwise over 8 min. The cooling bath was then removed and the reaction was warmed to 80 °C and stirred for 3 h. It was then allowed to cool to 23 °C before being cooled further to 0 °C. The reaction was then quenched by addition of aqueous NaOH (1.0 M, 40 mL) over 1 min before being warmed to 23 °C. 6.0 M NaOH (40 mL) was then added in one portion and the mixture was stirred at 23 °C for 5 min before being transferred to a separatory funnel. The layers were separated and the aqueous layer was extracted with CH_2Cl_2 (3 x 50 mL). The combined organic layers were then dried over Na_2SO_4, filtered, and concentrated under reduced pressure. The resulting oil was purified by flash chromatography (5% Et_3N in 9:1 hexanes:EtOAc → 5% Et_3N in 7:2 hexanes:EtOAc) using silica gel neutralized with Et_3N to afford bromobipyridine **47** as a beige solid (800 mg, 59% yield over two steps). **Bromobipyridine 47**: R_f 0.25 (3:1 EtOAc:Hexanes); ^1H NMR (500 MHz, $CDCl_3$): δ 8.75 (dd, J = 4.9, 0.8, 1H), 8.66 (d, J = 4.6, 1H), 8.03 (d, J = 7.9, 1H), 7.83 (td, J = 7.6, 1.4, 1H), 7.73 (d, J = 7.7, 1H), 7.36 (dd, J = 7.6, 4.9, 1H), 7.22 (dd, J = 8.3, 4.7, 1H). Spectral data match those previously reported in the literature.[2]

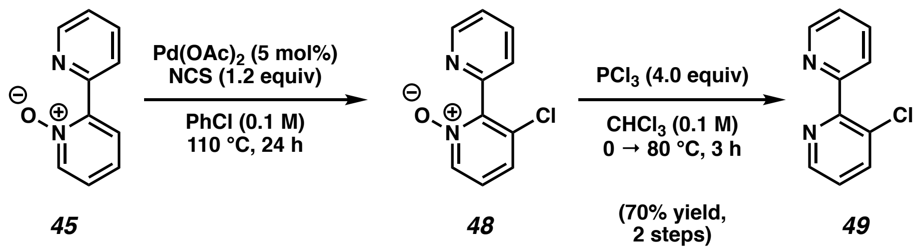

Chlorobipyridine 49. To a flask containing 2,2'-bipyridyl *N*-oxide (**45**, 3.81 g, 22.1 mmol), $Pd(OAc)_2$ (248 mg, 1.11 mmol, 5 mol%), and *N*-Chlorosuccinimide (3.55 g, 26.6 mmol, 1.2 equiv) was added PhCl (220 mL, 0.10 M). This suspension was then heated to 110 °C and stirred for 24 h. The reaction mixture was then allowed to cool to 23 °C, at which point it was transferred with CH_2Cl_2 (15 mL) to a separatory funnel containing aqueous NaOH (1.0 M, 100 mL). The layers were separated and the aqueous layer was extracted with CH_2Cl_2 (3 x 60 mL). The combined organic layers were then dried over Na_2SO_4, filtered, and concentrated under reduced pressure to afford the corresponding chlorobipyridine *N*-oxide **48** as a brown oil. This was carried forward without further purification.

The crude oil was dissolved in $CHCl_3$ (200 mL, 0.10 M) and cooled to 0 °C. To this stirred solution was added PCl_3 (7.28 mL, 83.2 mmol, 4.0 equiv) dropwise at 0 °C over 12 min. The cooling bath was then removed and the reaction was warmed to 80 °C and stirred for 3 h. It was then allowed to cool to 23 °C before being cooled further to 0 °C. The reaction was then quenched by addition of aqueous NaOH (1.0 M, 100 mL) over 2 min before being warmed to 23 °C. Aqueous NaOH (6.0 M, 50 mL) was then added in one portion and the mixture was stirred at 23 °C for 5 min before being transferred to a separatory funnel. The layers were separated and the aqueous layer was extracted with CH_2Cl_2 (3 x 100 mL). The combined organic layers were then dried over Na_2SO_4, filtered, and concentrated under reduced pressure. The resulting brown oil was purified by flash chromatography (5% Et_3N in 3:1 hexanes:EtOAc → 5% Et_3N in 1:1 hexanes:EtOAc → 5% Et_3N in 1:3 hexanes:EtOAc) using silica gel neutralized with Et_3N to afford chlorobipyridine **49** as an off-white solid (2.96 g, 70% yield over two steps). **Chlorobipyridine 49**: R_f 0.30 (3:1 EtOAc:hexanes); ^1H NMR (400 MHz, CD_3CN): δ 8.77 (d, *J* = 4.8, 1H), 8.64 (dd, *J* = 4.8, 1.4, 1H), 7.83 (td, *J* = 8.7, 1.4, 2H), 7.77 (d, *J* = 7.9, 1H), 7.36 (ddd, *J* = 7.7, 4.9, 1.3, 1H), 7.31 (dd, *J* = 8.31, 4.7, 1H). Spectral data match those previously reported in the literature.[2]

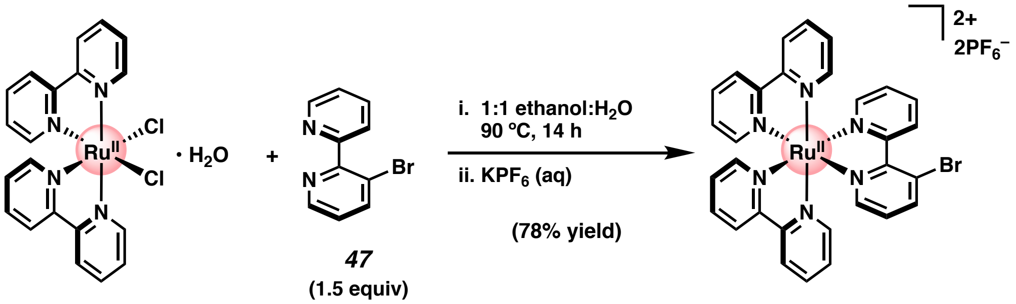

Bromo-Ru(bpy)$_3$[PF$_6$]$_2$ 14a. To a flask containing *cis*-bis(2,2'-bipyridine)dichlororuthenium(II) hydrate (**50**, 899 mg, 1.86 mmol, 1.0 equiv) and bromobipyridine **47** (667 mg, 2.84 mmol, 1.5 equiv) was added EtOH (120 mL) and H_2O (120 mL). The flask was topped with an air condenser and the system placed under N_2. The reaction was heated to 90 °C and stirred for 14 h, during which the solution changed in color from deep purple to red. The reaction was then allowed to cool to 23 °C before saturated aqueous KPF_6 (50 mL) was added over 1 min while stirring to produce a red precipitate. The mixture was then filtered over a pad of celite (packed with Et_2O), washed with Et_2O (3 x 20 mL), and the filtrate was discarded. The remaining solid residue was then redissolved in CH_3CN (90 mL) and passed through the same celite plug. The resulting deep red filtrate was then concentrated under reduced pressure and then passed through a pad of neutral alumina with CH_3CN (250 mL). The red band was collected and concentrated under reduced pressure to afford a red semi-solid. This material was then recrystallized from 1:1 MeOH:H_2O (10 mL) and the resulting red crystals were washed with 1:1 MeOH:H_2O (2 x 2 mL, cooled to 0 °C) and dried under reduced pressure (<1 torr) for 12 h at 60 °C to afford bromo-Ru(bpy)$_3$[PF$_6$]$_2$ **14a** as a red crystalline solid (1.36 g, 78% yield based on anhydrous **50**). **Bromo-Ru(bpy)$_3$[PF$_6$]$_2$ 14a**: mp >250 °C; R_f 0.64 (7:2:1 MeCN:H_2O:sat. aq. KNO_3); ^{1}H NMR (400 MHz, CD_3CN): δ 9.56 (dq, *J* = 8.6, 0.7, 1H), 8.49 (dd, *J* = 8.3, 3.4, 4H), 8.30 (dd, *J* = 8.3, 1.3, 1H), 8.10–8.02 (m, 5H), 7.84 (dd, *J* = 5.5, 1.4, 2H), 7.76 (dq, *J* = 5.7, 0.7, 1H), 7.70 (dq, *J* = 5.7, 0.7, 1H), 7.68–7.63 (m, 2H); ^{13}C NMR (125 MHz, CD_3CN, 26 of 30 signals observed): δ 157.79, 157.78, 157.69, 157.65, 157.5, 154.9, 153.2, 152.8, 152.7, 152.5, 152.3, 145.7, 138.93, 138.91, 138.90, 138.0, 129.3, 128.7, 128.64, 128.58, 128.55, 128.46, 127.9, 125.29, 125.28, 121.8; IR (film): 1606, 1467, 1447, 1425, 837, 762 cm^{-1}; HR-ESI-MS (*m/z*) [M – PF$_6$]$^+$ calcd for $C_{30}H_{23}N_6BrPF_6Ru^+$, 792.98529; found 792.9849.

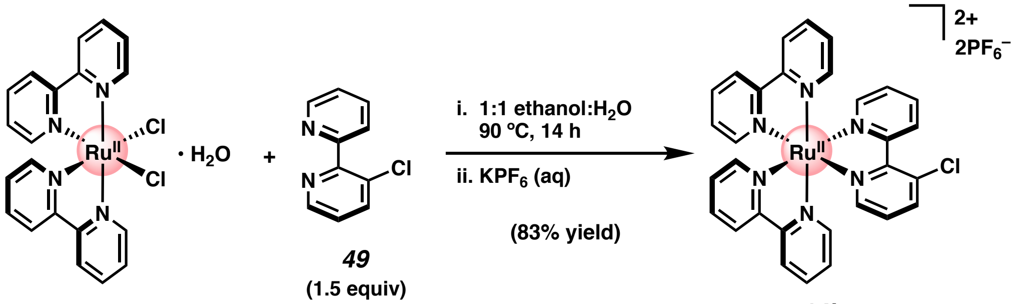

Chloro-Ru(bpy)$_3$[PF$_6$]$_2$ 14b. To a flask containing *cis*-bis(2,2'-bipyridine)dichlororuthenium(II) hydrate (**50**, 4.00 g, 8.26 mmol, 1.0 equiv) and chlorobipyridine **49** (2.36 g, 12.4 mmol, 1.5 equiv) was added EtOH (330 mL) and H_2O (330 mL). The flask was topped with an air condenser and the system placed under N_2. The reaction was heated to 90 °C and stirred for 14 h, during which the solution changed in color from deep purple to red. The reaction was then allowed to cool to 23 °C before saturated aqueous KPF_6 (300 mL) was added over 5 min while stirring to produce a red precipitate. The mixture was then filtered over a pad of celite (packed with Et_2O), washed with Et_2O (3 x 50 mL), and the filtrate was discarded. The remaining solid residue was redissolved in CH_3CN (200 mL) and passed through the same celite plug. The resulting deep red filtrate was then concentrated under reduced pressure and then passed through a pad of neutral alumina with CH_3CN (600 mL). The red band was collected and concentrated under reduced pressure to afford a red semi-solid. The purified material was then divided into three portions and each was recrystallized from 1:1 MeOH:H_2O (10 mL) and the resulting red crystals were washed with 1:1 MeOH:H_2O (2 x 2 mL for each portion, cooled to 0 °C) and dried under reduced pressure (<1 torr) for 12 h at 50 °C to afford chloro-Ru(bpy)$_3$[PF$_6$]$_2$ **14b** as a red crystalline solid (6.13 g, 83% yield based on anhydrous **50**). **Chloro-Ru(bpy)$_3$[PF$_6$]$_2$ 14b**: mp >250 °C; R_f 0.60 (7:2:1 MeCN:H_2O:sat. aq. KNO_3); ^{1}H NMR (400 MHz, CD_3CN): δ 9.41 (dq, J = 8.5, 0.8, 1H), 8.50 (dd, J = 8.2, 1.0, 4H), 8.12–8.02 (m, 6H), 7.86 (dq, J = 5.6, 0.7, 1H), 7.81 (dd, J = 5.5, 1.3, 1H), 7.76 (dq, J = 5.6, 0.7, 1H), 7.70 (dq, J = 5.6, 0.7, 1H), 7.68–7.64 (m, 2H), 7.44–7.35 (m, 5H), 7.31 (dd, J = 8.4, 5.5, 1H); ^{13}C NMR (125 MHz, CD_3CN, 24 of 30 signals observed) δ 157.80, 157.78, 157.71, 157.67, 157.1, 154.0, 153.2, 152.8, 152.47, 152.45, 151.9, 142.0, 138.94, 138.91, 138.4, 134.2, 129.4, 128.7, 128.65, 128.61, 128.57, 128.5, 128.1, 125.30, 125.29; IR (film): 1604, 1467, 1447, 1427, 1243, 837 cm^{-1}; HR-ESI-MS (*m/z*) [M – PF$_6$]$^+$ calcd for $C_{30}H_{23}N_6ClPF_6Ru^+$, 749.03580; found 749.0350.

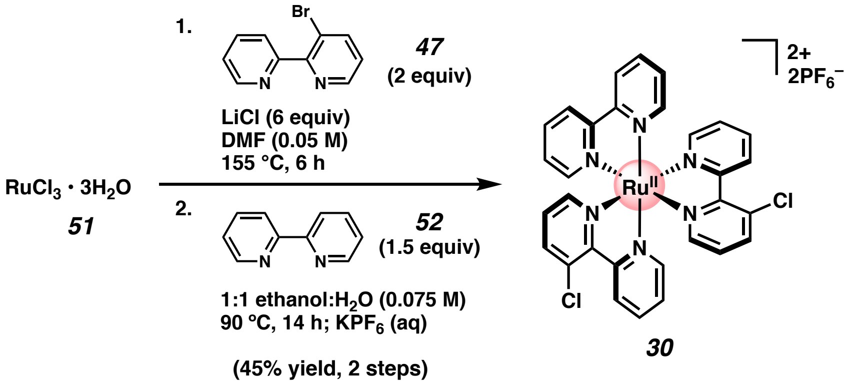

Bis(chloro)-Ru(bpy)$_3$[PF$_6$]$_2$ 30. To a flask containing ruthenium(III) chloride trihydrate (**51**, 309 mg, 1.18 mmol, 1.0 equiv), bromobipyridine **47** (559 mg, 2.38 mmol, 2.0 equiv), and LiCl (301 mg, 7.10 mmol, 6.0 equiv) was added DMF (25 mL, 0.05 M). The flask was topped with an air condenser and the system placed under N_2. The reaction was heated to 155 °C and stirred for 6 h. The reaction mixture was then allowed to cool to 23 °C before acetone (25 mL) was added in one portion and the solution was added dropwise to vigorously stirring Et_2O (300 mL). The resultant mixture was then filtered over a pad of celite (packed with Et_2O) and washed with Et_2O (3 x 30 mL). The solid residue was then redissolved in CH_2Cl_2 (200 mL) and concentrated under reduced pressure to afford a deep purple solid. This was carried forward without further purification.

To the crude solid was added 2,2'-bipyridine (**52**, 281 mg, 1.80 mmol, 1.5 equiv), EtOH (80 mL), and H_2O (80 mL) sequentially. The flask was topped with an air condenser and the system placed under N_2. The reaction was heated to 90°C and stirred for 14 h, during which the solution changed in color from deep purple to red. The reaction was then allowed to cool to 23 °C before saturated aqueous KPF_6 (70 mL) was added over 2 min while stirring to produce a red precipitate. The mixture was then filtered over a pad of celite (packed with Et_2O), washed with Et_2O (3 x 30 mL), and the solid residue was redissolved in MeCN (200 mL). The deep red filtrate was then concentrated under reduced pressure and then purified by flash chromatography on neutral alumina (100% MeCN). The red band was collected and concentrated under reduced pressure to afford a red semi-solid. This material was then recrystallized from 1:1 MeOH:H_2O (5 mL) and the resulting red crystals were washed with 1:1 MeOH:H_2O (2 x 2 mL, cooled to 0 °C) and dried under reduced pressure (<1 torr) for 12 h at 60 °C to afford bis(chloro)-Ru(bpy)$_3$[PF$_6$]$_2$ **30** as a red crystalline solid (491 mg, 45% yield over 2 steps). **Bis(chloro)-Ru(bpy)$_3$[PF$_6$]$_2$ 30**: mp >250 °C; R_f 0.63 (7:2:1 MeCN:H_2O:sat. aq. KNO_3); ^{1}H NMR (500 MHz, CD_3CN): δ 9.43–9.39 (m, 2H), 8.50 (d, *J* = 8.2, 2H), 8.13–8.04 (m, 6H), 7.88 (dq, *J* = 5.6, 0.7, 1H), 7.85–7.82 (m, 1H), 7.81–7.77 (m, 1H),

7.75 (ddd, J = 5.6, 3.7, 1.4, 1H) 7.71 (app. d, J = 5.6, 1H), 7.67–7.63 (m, 1H), 7.45–7.37 (m, 4H), 7.34–7.28 (m, 2H); ^{13}C NMR (125 MHz, CD_3CN) δ 157.59, 157.57, 157.56, 157.5, 156.92, 156.85, 156.81, 153.85, 153.78, 153.75, 153.3, 153.04, 153.01, 152.64, 152.63, 152.03, 152.02, 151.74, 151.71, 142.24, 142.22, 142.21, 139.13, 139.11, 139.10, 139.09, 138.58, 138.57, 134.32, 134.29, 129.53, 129.51, 128.8, 128.72, 128.67, 128.61, 128.60, 128.5, 128.2, 128.1, 128.0, 125.41, 125.39, 125.37; IR (film): 1603, 1468, 1447, 1419, 1214, 835, 797 cm^{-1}; HR-ESI-MS (*m/z*) $[M - PF_6]^+$ calcd for $C_{30}H_{22}N_6Cl_2PF_6Ru^+$, 782.99683; found 782.9984.

Note: Though unexpected, it is postulated that LiCl facilitates chloro-debromination during the reaction. ***30*** *was obtained as a mixture of geometric isomers; these data represent empirically observed chemical shifts from the* ^{13}*C NMR spectrum.*

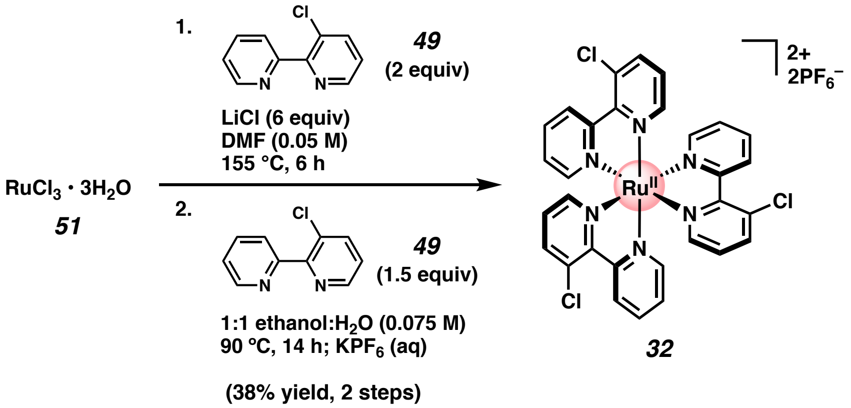

Tris(chloro)-Ru(bpy)$_3$[PF$_6$]$_2$ 32. To a flask containing ruthenium(III) chloride trihydrate (**51**, 401 mg, 1.54 mmol, 1.0 equiv), chlorobipyridine **49** (584 mg, 3.07 mmol, 2.0 equiv), and LiCl (390 mg, 9.21 mmol, 6.0 equiv) was added DMF (30 mL, 0.05 M). The flask was topped with an air condenser and the system placed under N_2. The reaction was heated to 155 °C and stirred for 6 h. The reaction mixture was then allowed to cool to 23 °C before acetone (35 mL) was added in one portion and the mixture was added dropwise to vigorously stirring Et_2O (300 mL). The resultant mixture was then filtered over a pad of celite (packed with Et_2O) and washed with Et_2O (4 x 30 mL). The solid residue was then redissolved in CH_2Cl_2 (250 mL) and concentrated under reduced pressure to afford a deep purple solid. This was carried forward without further purification.

To the crude solid material was added chlorobipyridine **49** (443 mg, 2.32 mmol, 1.5 equiv), EtOH (100 mL), and H_2O (100 mL) sequentially. The flask was topped with an air condenser and the system placed under N_2. The reaction was heated to 90°C and stirred for 14 h, during which the solution changed in color from deep purple to red. The reaction was then allowed to cool to 23 °C before saturated aqueous KPF_6 (70 mL) was added over 2 min while stirring to produce a red precipitate. The mixture was then filtered over a pad of celite (packed with Et_2O), washed with Et_2O (3 x 30 mL), and the solid residue was redissolved in MeCN (250 mL). The deep red filtrate was then concentrated under reduced pressure and then purified by flash chromatography on neutral alumina (100% MeCN). The red band was collected and concentrated under reduced pressure to afford a red semi-solid. This material was then recrystallized from 1:1 MeOH:H_2O (5 mL) and the resulting red crystals were washed with 1:1 MeOH:H_2O (2 x 2 mL, cooled to 0 °C) and dried under reduced pressure (<1 torr) for 12 h at 60 °C to afford tris(chloro)-Ru(bpy)$_3$[PF$_6$]$_2$ **32** as a red crystalline solid (567 mg, 38% yield over 2 steps). **Tris(chloro)-Ru(bpy)$_3$[PF$_6$]$_2$ 32**: mp >250 °C; R_f 0.69 (7:2:1 MeCN:H_2O:sat. aq. KNO_3); ^1H NMR (500 MHz, CD_3CN): δ 9.41 (d, J = 8.5, 3H), 8.14–8.06 (m, 6H), 7.85–7.72 (m, 6H), 7.41–7.38 (qd, J = 6.8, 1.3, 3H), 7.35–7.28 (m, 3H); ^{13}C NMR (125 MHz, CD_3CN) δ 156.7, 156.5, 153.63, 153.62, 153.59, 153.20, 153.18, 151.89, 151.87, 142.40, 142.38, 142.37, 138.74, 138.71, 134.38, 134.36, 134.35, 134.34, 129.61, 129.60, 129.57, 128.83, 128.79, 128.70, 128.66, 128.3, 128.2, 128.13, 128.07; IR (film): 1473, 1428, 1420, 1215, 837, 797 cm^{-1}; HR-ESI-MS (*m/z*) [M – PF$_6$]$^+$ calcd for $C_{30}H_{21}N_6Cl_3PF_6Ru^+$, 816.95786; found 816.9572.

*Note: **32** was obtained as a mixture of geometric isomers. These data represent empirically observed chemical shifts from the ^{13}C NMR spectrum.*

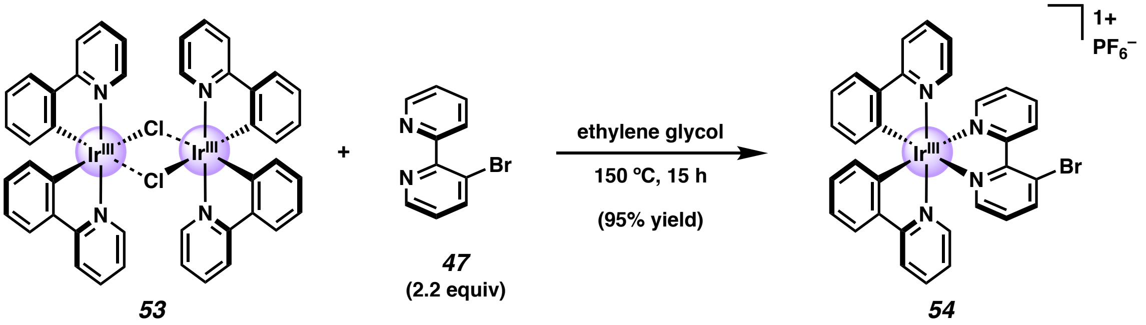

Bromo-Ir(ppy)$_2$bpy 54. To a 20 mL scintillation vial was added di-μ-chlorotetrakis[2-(2-pyridinyl-kN)phenyl-kC]diiridium(III) (**53**, 100.0 mg, 0.093 mmol, 1.0 equiv) in a glovebox. The

vial was removed from the glovebox and placed under nitrogen. To a separate 1-dram vial was added bromobipyridine **47** (48.2 mg, 0.205 mmol, 2.2 equiv) and ethylene glycol (4.0 mL, 0.023 M), and the mixture was then sparged with N_2 for 15 minutes. This solution was then transferred to the vial containing **53**. The septa cap was replaced with a Teflon-lined screw cap and the reaction was transferred to an Al-block and stirred at 150 °C for 15 h. After cooling to 23 °C, the reaction was transferred to a separatory funnel containing deionized water (48 mL). The aqueous phase was washed with hexanes (2 x 24 mL) and then Et_2O (24 mL). The aqueous layer was then heated to 85 °C for 5 minutes to evaporate any remaining organic solvent. The solution cooled to 23 °C, and then then aqueous NH_4PF_6 (0.27 M, 12 mL) was added, resulting in an orange-yellow precipitate. This mixture was filtered through a plug of celite and washed with water (20 mL) to remove ethylene glycol. The celite pad was then eluted with acetonitrile (50 mL) to redissolve the product, and the resulting filtrate was collected and concentrated under reduced pressure to afford bromo-Ir(ppy)$_2$bpy complex **54** as a dark yellow solid (156 mg, 95% yield). **Bromo-Ir(ppy)$_2$bpy 54**. R_f 0.60 (7:2:1 CH_3CN:H_2O:sat. aq. KNO_3); ^{1}H NMR (500 MHz, CD_3CN): δ 9.42 (dt, *J* = 8.3, 1.6, 0.9, 1H), 8.36 (dd, *J* = 8.4, 1.4, 1H), 8.13 (ddd, *J* = 8.7, 7.9, 1.8 1H), 8.09–8.04 (m, 4H), 7.88–7.83 (m, 2H), 7.78 (ddd, *J* = 7.9, 3.3, 1.1, 2H), 7.73–7.70 (m, 2H), 7.49 (ddd, *J* = 7.7, 5.5, 1.2, 1H), 7.29 (dd, *J* = 8.6, 5.4, 1H), 7.06–7.01 (m, 4H), 6.90 (tdd, *J* = 7.4, 2.4, 1.3, 2H), 6.22 (ddd, *J* = 7.6, 4.6, 1.0, 2H); ^{13}C NMR (125 MHz, CD_3CN) δ 168.6, 168.5, 156.2, 154.9, 152.6, 151.7, 151.5, 150.83, 150.79, 150.7, 147.5, 145.4, 145.3, 140.1, 140.0, 139.8, 132.9, 132.8, 131.9, 131.8, 130.2, 129.9, 129.5, 126.3, 124.9, 124.8, 124.1, 124.0, 123.8, 123.0, 121.3, 121.2; IR (film): 3064, 2921, 1607, 1478, 1420, 1342 cm^{-1}; HR-ESI-MS (*m/z*) [M – PF_6]$^+$ calcd for $C_{32}H_{23}BrN_4Ir^+$, 735.0735; found 735.0738.

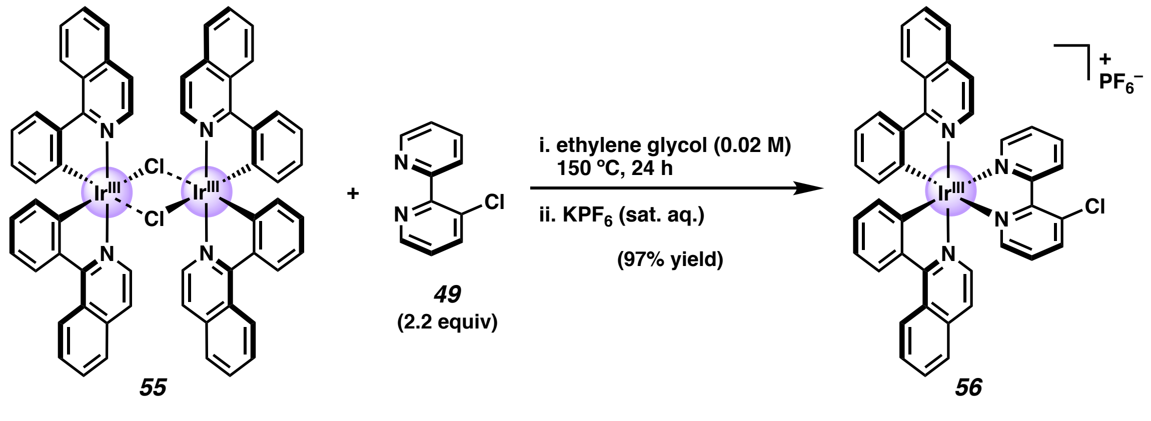

Isoquinolinyl Complex 56. To an 8-dram vial containing di-μ-chlorotetrakis[2-(1-isoquinolinyl-N)phenyl-C]diiridium(III) (**55**, 200.0 mg, 0.157 mmol, 1.0 equiv) and chlorobipyridine **49** (65.9 mg, 0.346 mmol, 2.2 equiv) was added ethylene glycol (6.8 mL, 0.023 M). The resulting suspension was then sparged with N_2 for 15 minutes before being transferred to an Al-block and stirred at 150 °C for 24 h. After cooling to 23 °C, the reaction was diluted with water (20 mL) before being transferred with water (5 mL) to a flask containing sat. aq. KPF_6 (50 mL), resulting in formation of a red precipitate. This mixture was then filtered through a plug of celite and washed sequentially with water (300 mL), hexanes (200 mL), and Et_2O (200 mL). The celite pad was then eluted with CH_2Cl_2 (200 mL) to redissolve the product, and the resulting filtrate was collected, dried over Na_2SO_4, concentrated under reduced pressure, and dried under reduced pressure (<1 torr) at 100 °C for 12 h to afford Ir(ppy)$_2$bpy complex **56** as a red solid (286.3 mg, 97% yield).
Isoquinolinyl Complex 56. R_f 0.62 (14:1:1 CH_3CN:H_2O:sat. aq. KNO_3); ^{1}H NMR (500 MHz, CD_3CN): δ 9.34 (dt, *J* = 8.5, 1.0, 1H), 9.06–8.94 (m, 2H), 8.37 (d, *J* = 8.1, 2H), 8.16–8.08 (m, 2H), 8.02–7.98 (m, 2H), 7.97 (ddd, *J* = 5.4, 1.7, 0.8, 1H), 7.93 (dd, *J* = 5.3, 1.4, 1H), 7.87–7.80 (m, 4H), 7.63 (d, *J* = 6.4, 1H), 7.61 (d, *J* = 6.5, 1H), 7.47–7.40 (m, 3H), 7.35 (dd, *J* = 8.3, 5.2, 1H), 7.11 (dddd, *J* = 8.8, 6.6, 1.6, 1.4, 2H), 6.86 (t, *J* = 7.4, 2H), 6.25 (ddd, *J* = 7.7, 4.4, 1.1, 2H); ^{13}C NMR (125 MHz, CD_3CN, 35 of 40 signals observed) δ 169.3, 169.1, 155.2, 154.4, 153.7, 153.2, 152.2, 150.8, 146.5, 146.4, 143.4, 141.93, 141.90, 139.7, 138.1, 138.05, 134.8, 132.92, 132.87, 132.7, 131.8, 131.53, 131.47, 129.9, 129.8, 129.4, 129.2, 128.51, 128.50, 127.7, 127.1, 123.4, 123.3, 122.9, 122.8; IR (film): 3080, 3045, 1576, 1541, 1433, 840 cm^{-1}; HR-ESI-MS (*m/z*) [M – PF_6]$^+$ calcd for $C_{40}H_{27}ClN_4Ir^+$, 791.1553; found 791.1603.

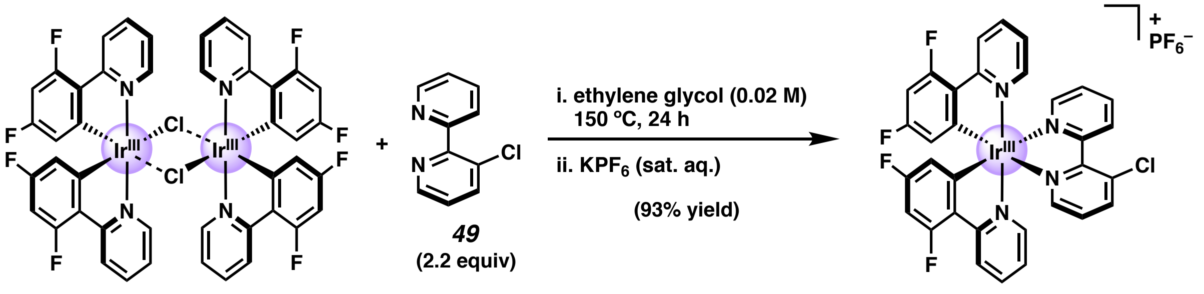

Fluorinated Ir Complex 58. To an 8-dram vial containing dichlorotetrakis[3,5-difluoro-2-(2-pyridinyl)phenyl]diiridium(III) (**57**, 200.0 mg, 0.165 mmol, 1.0 equiv) and chlorobipyridine **49** (69.0 mg, 0.362 mmol, 2.2 equiv) was added ethylene glycol (7.2 mL, 0.023 M). The resulting

suspension was then sparged with N_2 for 15 minutes before being transferred to an Al-block and stirred at 150 °C for 24 h. After cooling to 23 °C, the reaction was diluted with water (20 mL) before being transferred with water (5 mL) to a flask containing sat. aq. KPF_6 (50 mL), resulting in formation of a red precipitate. This mixture was then filtered through a plug of celite and washed sequentially with water (300 mL), hexanes (200 mL), and Et_2O (200 mL). The celite pad was then eluted with CH_2Cl_2 (200 mL) to redissolve the product, and the resulting filtrate was collected, dried over Na_2SO_4, concentrated under reduced pressure, and dried under reduced pressure (<1 torr) at 100 °C for 12 h to afford $Ir(ppy)_2bpy$ complex **58** as a yellow solid (277.7 mg, 93% yield).

Fluorinated Ir Complex 58. R_f 0.64 (14:1:1 CH_3CN:H_2O:sat. aq. KNO_3); ^1H NMR (500 MHz, CD_3CN): δ 9.36 (dt, *J* = 8.4, 0.9, 1H), 8.31 (d, *J* = 8.5, 2H), 8.23–8.15 (m, 2H), 8.12 (ddd, *J* = 5.4, 1.7, 0.7, 1H), 8.07 (dd, *J* = 5.3, 1.4, 1H), 7.95–7.87 (ddddd, *J* = 8.5, 7.9, 4.2, 1.6, 0.7, 2H), 7.71 (d, *J* = 5.8, 2H), 7.54 (ddd, *J* = 7.8, 5.5, 1.2, 1H), 7.45 (dd, *J* = 8.6, 5.3, 1H), 7.09 (dddd, *J* = 10.9, 7.8, 5.9, 1.4, 2H), 6.69 (dddd, *J* = 12.8, 9.4, 2.5, 2.1), 5.69 (ddd, *J* = 8.6, 7.2, 2.4); ^{13}C NMR (125 MHz, CD_3CN) δ 165.5, 165.4. 164.43, 164.38, 164.34, 164.28, 163.5, 163.43, 163.40, 163.38, 163.3, 161.31, 161.26, 155.21, 155.15, 155.1, 154.43, 154.37, 153.0, 152.6, 151.3, 150.84, 150.75, 144.1, 140.59, 140.57, 140.4, 135.1, 130.0, 129.8, 129.5, 128.91, 128.89, 128.87, 128.85, 128.81, 128.79, 128.77, 128.75, 125.0, 124.8, 124.7, 114.81, 114.79, 114.67, 114.65, 114.6, 114.51, 114.48, 100.03, 99.98, 99.81, 99.76, 99.6, 99.5; IR (film): 3088, 2973, 1739, 1604, 1575, 1479, 1430, 1406, 840 cm^{-1}; HR-ESI-MS (*m/z*) $[M - PF_6]^+$ calcd for $C_{32}H_{19}ClF_4N_4Ir^+$, 763.0864; found 763.0864.

(Note: Complex splitting patterns observed due to presence of fluorine atoms; these data represent empirically observed chemical shifts from the ^{13}C NMR spectrum)

B. Optimization of on-the-Complex Annulation with Benzyne

Representative Procedure for reaction optimization. Benzyne adduct 16 (Supplementary Table 1, entry 11): To a 1-dram vial was added $Pd(OAc)_2$ (1.6 mg, 7.3 µmol, 10 mol%), bromo-$Ru(bpy)_3[PF_6]_2$ **14a** (68.6 mg, 73.1 µmol, 1.0 equiv), P(*o*-tolyl)$_3$ (2.2 mg, 7.3 µmol, 10 mol%), MeCN (0.50 mL, 0.15 M), PhMe (0.50 mL, 0.15 M), silyl triflate (43.6 mg, 73.1 µmol, 2.0 equiv, **15**), an oven-dried magnetic stir bar, and CsF (111 mg, 731 µmol, 10.0 equiv) sequentially. The reaction was then purged with N_2 for 5 min before being sealed with a Teflon-lined screw cap under a flow of N_2, sealed with Teflon tape and electrical tape, transferred to an Al-block, and stirred at 110 °C for 30 min. After cooling to 23 °C, the mixture was filtered through a plug of celite with MeCN (6 mL) and concentrated under reduced pressure. The yield was determined by ^{1}H NMR analysis with 1,3,5-trimethoxybenzene as an external standard.

Optimization efforts that deviate from the above conditions are indicated below.

Supplementary Table 1. Optimization studies of on-the-complex annulation with benzyne. Yields were determined using ^{1}H NMR analysis with 1,3,5-trimethoxybenzene (TMB) as an external standard.

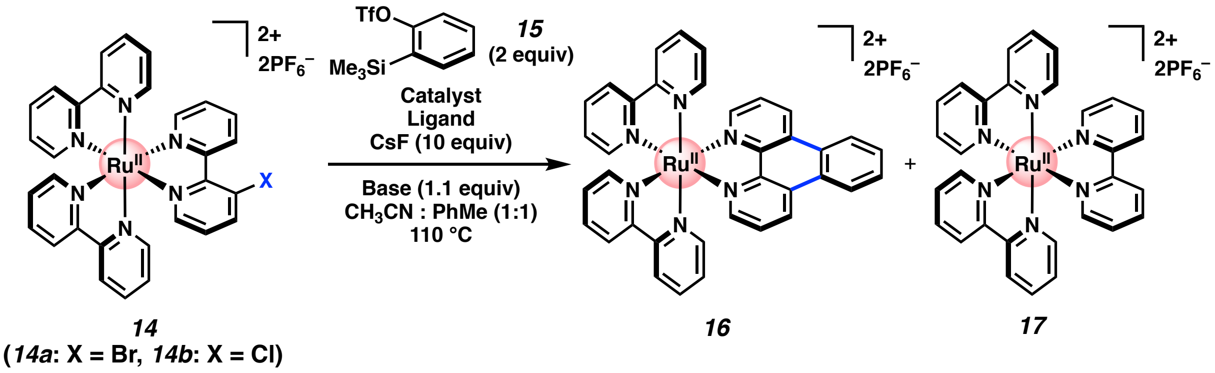

Entry	*Catalyst*	*Ligand*	***X***	*Catalyst Loading*	*Ligand Loading*	*Base*	*CH$_3$CN : PhMe*	*Time*	***16***	***17***
1	Pd(dba)$_2$	P(*o*-tolyl)$_3$	Br	5 mol%	5 mol%	–	1 : 1	2 h	2%	48%
2	Pd(PPh$_3$)$_4$	P(*o*-tolyl)$_3$	Br	5 mol%	5 mol%	–	1 : 1	2 h	2%	48%
3	XPhos Pd G2		Br	5 mol%	5 mol%	–	1 : 1	2 h	4%	40%
4	Pd(OAc)$_2$	P(*o*-tolyl)$_3$	Br	5 mol%	5 mol%	–	1 : 1	2 h	26%	24%
5	Pd(OAc)$_2$	P(*o*-tolyl)$_3$	Br	10 mol%	30 mol%	–	1 : 1	2 h	60%	18%
6	Pd(OAc)$_2$	P(*o*-tolyl)$_3$	Br	10 mol%	30 mol%	Ag_2CO_3	1 : 1	2 h	54%	16%
7	Pd(OAc)$_2$	P(*o*-tolyl)$_3$	Br	10 mol%	30 mol%	K_2CO_3	1 : 1	2 h	41%	19%
8	Pd(OAc)$_2$	P(4-ClC$_6$H$_4$)$_3$	Br	10 mol%	30 mol%	–	1 : 1	2 h	32%	29%
9	Pd(OAc)$_2$	P(4-MeOC$_6$H$_4$)$_3$	Br	10 mol%	30 mol%	–	1 : 1	2 h	16%	35%
10	Pd(OAc)$_2$	SPhos	Br	10 mol%	30 mol%	–	1 : 1	2 h	44%	26%
11	Pd(OAc)$_2$	P(*o*-tolyl)$_3$	Br	10 mol%	10 mol%	–	1 : 1	30 min	71%	16%
12	Pd(OAc)$_2$	P(*o*-tolyl)$_3$	Br	10 mol%	10 mol%	–	3 : 1	30 min	61%	19%
13	Pd(OAc)$_2$	P(*o*-tolyl)$_3$	Br	10 mol%	10 mol%	–	9 : 1	30 min	44%	18%
14	Pd(OAc)$_2$	P(*o*-tolyl)$_3$	Br	10 mol%	10 mol%	–	100% CH$_3$CN	30 min	47%	16%
15	Pd(OAc)$_2$	P(*o*-tolyl)$_3$	Br	10 mol%	10 mol%	–	1 : 3	30 min	35%	28%
16	Pd(OAc)$_2$	P(*o*-tolyl)$_3$	Cl	10 mol%	10 mol%	–	1 : 1	30 min	78%	0%

Significant decomposition was observed when the halogenated bipyridyl ligand was not pre-ligated to the Ru complex (see below). It is postulated that deleterious intermediates such as ***51***

may form under these conditions due to the propensity for bipyridyl ligands to undergo N,N'-chelation to transition metals.

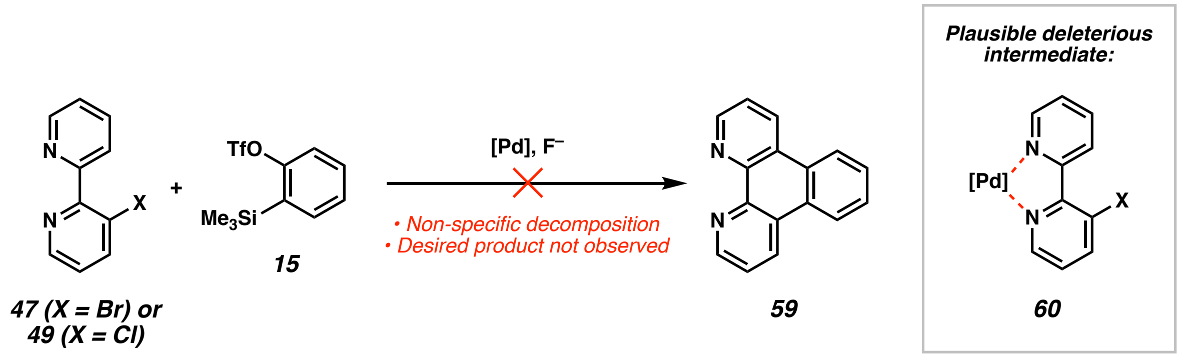

Supplementary Figure 1: Unsuccessful aryne annulation of free ligands **47** and **49**.

C. Crystallographic Data

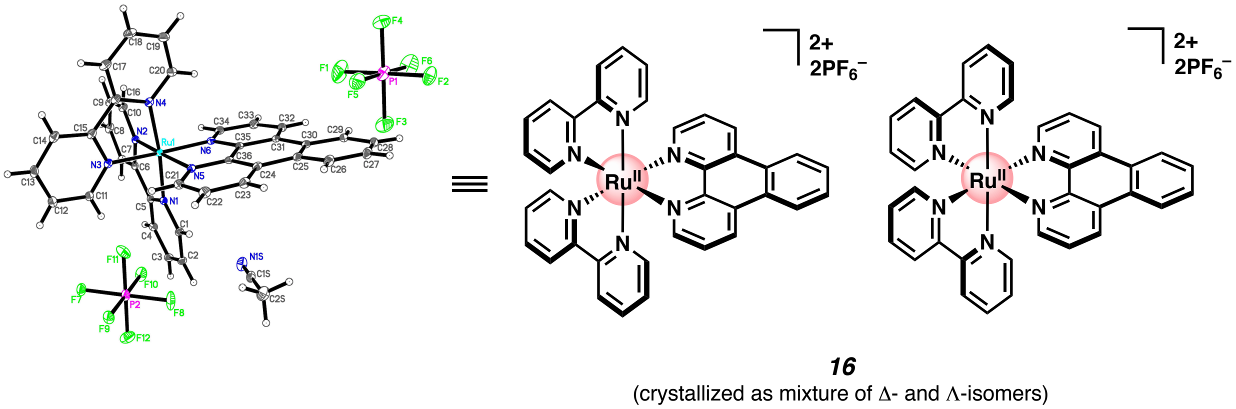

Supplementary Figure 2: ORTEP representation of X-ray crystallographic structure **16**. (CCDC Registry #2048567).

Supplementary Table 2. Crystal data and structure refinement for compound **16**.

Identification code	cu_garg1904_a_sq_s	
Empirical formula	C38 H29 F12 N7 P2 Ru	
Formula weight	974.69	
Temperature	100(2) K	
Wavelength	1.54178 Å	
Crystal system	Monoclinic	
Space group	$P2_1/c$	
Unit cell dimensions	a = 11.6931(3) Å	α = 90°.
	b = 30.8685(9) Å	β = 112.4510(10)°.
	c = 12.4524(3) Å	γ = 90°.
Volume	4154.01(19) $Å^3$	
Z	4	

Density (calculated)	1.559 Mg/m^3
Absorption coefficient	4.611 mm^{-1}
F(000)	1952
Crystal size	.16 x .08 x .04 mm^3
Theta range for data collection	2.863 to 70.067°.
Index ranges	-13<=h<=12, -36<=k<=37, -14<=l<=15
Reflections collected	34574
Independent reflections	7544 [R(int) = 0.0345]
Completeness to theta = 67.679°	96.9 %
Absorption correction	Semi-empirical from equivalents
Max. and min. transmission	0.75 and 0.62
Refinement method	Full-matrix least-squares on F^2
Data / restraints / parameters	7544 / 0 / 542
Goodness-of-fit on F^2	1.060
Final R indices [I>2sigma(I)]	R1 = 0.0259, wR2 = 0.0644
R indices (all data)	R1 = 0.0302, wR2 = 0.0657
Extinction coefficient	n/a
Largest diff. peak and hole	0.464 and -0.490 e.Å^{-3}

D. Synthesis of Silyl Triflate Aryne Precursors

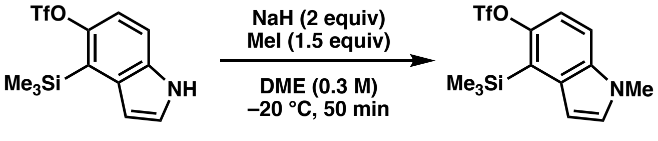

Silyl triflate 62. To a solution of Garg 4,5-indolyne precursor (**61**, 913 mg, 2.71 mmol, 1.0 equiv) in DME (9 mL, 0.3 M) at –20 °C was added MeI (256 µL, 4.10 mmol, 1.5 equiv) and NaH (60 wt% dispersion in mineral oil; 215 mg, 5.41 mmol, 2.0 equiv) sequentially. The reaction mixture was then stirred at –20 °C for 50 min before being quenched by addition of saturated aqueous NH_4Cl (5 mL). It was then allowed to warm to 23 °C before being transferred to a separatory funnel with H_2O (10 mL) and EtOAc (10 mL). The layers were then separated and the aqueous layer was extracted with EtOAc (3 x 15 mL). The combined organic layers were dried over $MgSO_4$, filtered, and concentrated under reduced pressure. The crude residue was then purified by flash chromatography (100% hexanes → 100:1 hexanes:EtOAc → 50:1 hexanes:EtOAc) to afford silyl triflate **62** as a viscous, pale yellow oil (927 mg, 97% yield). ***N*-Me silyl triflate 62**: R_f 0.63 (4:1 hexanes:EtOAc); ^{1}H NMR (500 MHz, $CDCl_3$): δ 7.33 (d, J = 9.0, 0.9, 1H), 7.20–7.14 (m, 2H), 6.68 (dd, J = 3.4, 0.6, 1H), 3.81 (s, 3H), 0.50 (s, 9H). Spectral data match those previously reported in the literature.[3]

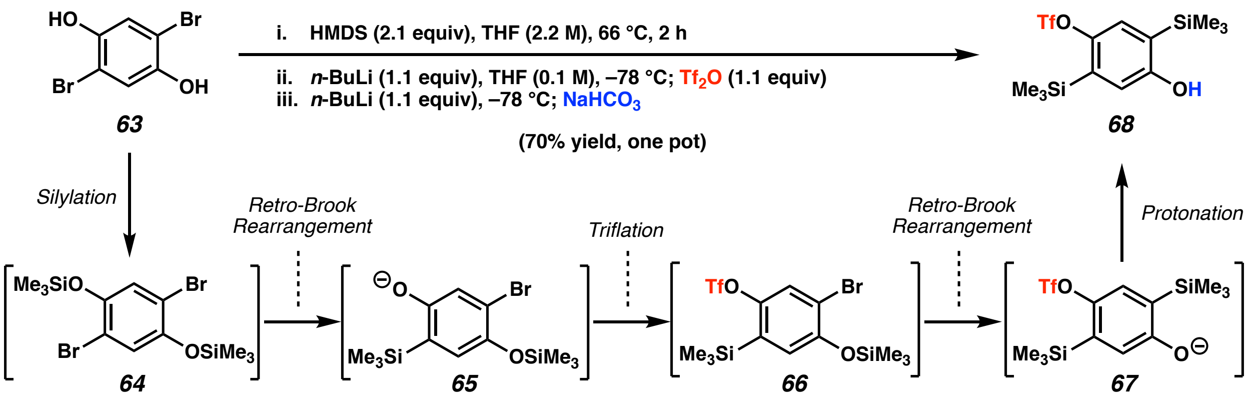

Silyl alcohol 68. To a stirred solution of 2,5-dibromohydroquinone (**63**, 2.60 g, 9.71 mmol, 1.0 equiv) in THF (4.40 mL, 2.20 M) was added HMDS (4.25 mL, 20.4 mmol, 2.1 equiv). The flask was topped with an air condenser and the system placed under N_2. The reaction was heated to 66 °C and stirred for 2 h. Then, the reaction mixture was allowed to cool to 23 °C and the volatiles were removed. THF (69 mL, 0.14 M) was then added and the mixture was cooled to –78 °C. *n*-BuLi (2.49 M in hexanes; 4.29 mL, 10.7 mmol, 1.1 equiv) was then added dropwise over 8 min. The reaction mixture was then stirred at –78 °C for 25 min. Tf_2O (1.80 mL, 10.7 mmol, 1.1 equiv) was then added dropwise over 5 min at –78 °C, and the mixture was allowed to stir at this temperature for 25 min. *n*-BuLi (2.49 M in hexanes; 4.29 mL, 10.7 mmol, 1.1 equiv) was then added dropwise over 8 min. The reaction mixture was then stirred at –78 °C for 25 min. Finally, the reaction was quenched by addition of saturated aqueous $NaHCO_3$ (40 mL) in one portion and the mixture was allowed to warm to 23 °C before being transferred to a separatory funnel with Et_2O (100 mL) and the layers were separated. The aqueous layer was then extracted with Et_2O (2 x 100 mL) and the combined organic layers were dried over Na_2SO_4, filtered, and concentrated under reduced pressure. The crude residue was then purified by flash chromatography (100% hexanes → 3% EtOAc in hexanes → 5% EtOAc in hexanes) to afford silyl alcohol **68** as a viscous yellow oil (2.62 g, 70% yield). **Silyl alcohol 68**: R_f 0.39 (9:1 hexanes:EtOAc) ^{1}H NMR (600 MHz, $CDCl_3$): δ 7.23 (s, 1H), 6.74 (s, 1H), 4.88 (s, 1H), 0.35 (s, 9H), 0.31 (s, 9H). Spectral data match those previously reported in the literature.[4]

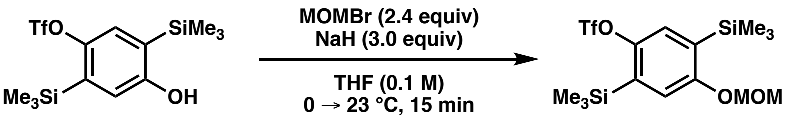

Methoxymethyl ether 36. To a stirred solution of silyl alcohol **69** (1.03 g, 2.66 mmol, 1.0 equiv) in THF (25 mL, 0.10 M) at 0 °C was added sodium hydride (dry, 95%; 202 mg, 7.99 mmol, 3.0 equiv) in one portion. The solution was then stirred at 0 °C for 15 min, followed by dropwise addition of bromomethyl methyl ether (522 µL, 6.39 mmol, 2.40 equiv) over 1 minute. Following addition, the reaction was allowed to warm to 23 °C and stirred for 15 min before being diluted with Et_2O (30 mL). H_2O (30 mL) was then added over 30 seconds and the mixture was transferred with Et_2O (5 mL) to a separatory funnel. The layers were separated and the aqueous layer was extracted with Et_2O (3 x 30 mL). The combined organic layers were then washed with H_2O (30 mL), dried over Na_2SO_4, and concentrated under reduced pressure. The crude residue was then purified by flash chromatography (100% hexanes) to afford methoxy methyl ether **36** as a white solid (814 mg, 71% yield). **Methoxymethyl ether 36**: mp: 37.5–38.5 ºC; R_f 0.56 (9:1 hexanes:EtOAc); ^{1}H NMR (500 MHz, $CDCl_3$): δ 7.25 (s, 1H), 7.14 (s, 1H), 5.19 (s, 2H), 3.48 (s, 3H), 0.36 (s, 9H), 0.28 (s, 9H); ^{13}C NMR (125 MHz, CD_3CN) δ 160.5, 149.6, 135.3, 132.8, 126.1, 126.0, 119.2, 118.7 (q, J = 320.5 Hz), 94.3, 56.3, –0.7, –1.2; IR (film): 2957, 1468, 1420, 1341, 1246, 1210, 1144, 1082, 1013, 945, 841, 757, 633 cm^{-1}; HR-GC-MS (*m/z*) $[M]^+$ calcd for $C_{15}H_{25}F_3O_5SSi_2^+$ 430.09133; found 430.0908.

E. Scope of Pd-Catalyzed Aryne Annulation

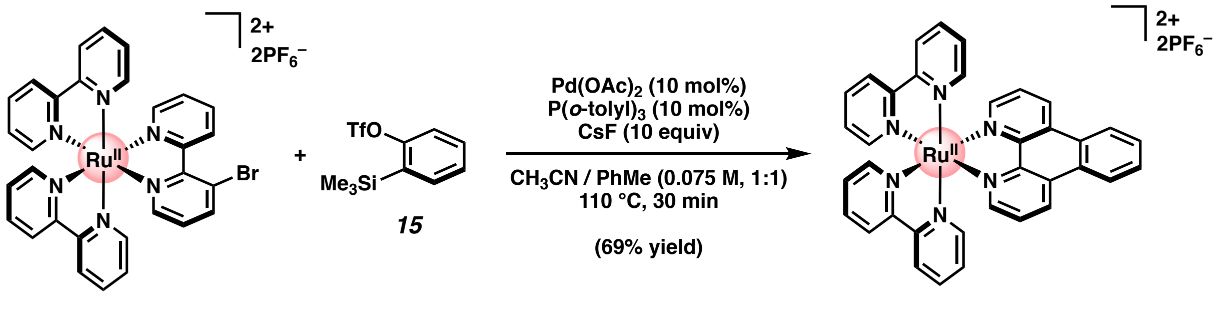

Representative Procedure A for aryne annulations (Figure 3, benzyne adduct 16 used as an example). To a 1-dram vial was added $Pd(OAc)_2$ (1.5 mg, 6.7 µmol, 10 mol%), Bromo-$Ru(bpy)_3[PF_6]_2$ **14a** (62.2 mg, 0.066 mmol, 1.0 equiv), P(*o*-tolyl)$_3$ (2.2 mg, 7.2 µmol, 10 mol%), MeCN (0.50 mL, 0.15 M), PhMe (0.50 mL, 0.15 M), silyl triflate **15** (40.1 mg, 0.134 mmol, 2.0 equiv), an oven-dried magnetic stir bar, and CsF (101 mg, 0.663 mmol, 10.0 equiv) sequentially. The reaction was then purged with N_2 for 5 min before being capped with a Teflon-lined screw cap under a flow of N_2, sealed with Teflon tape and electrical tape, transferred to an Al-block, and stirred at 110 °C for 30 min. After cooling to 23 °C, the mixture was filtered through a plug of celite with MeCN (6 mL), and concentrated under reduced pressure. The crude residue was adsorbed onto silica gel (500 mg) under reduced pressure and purified by flash chromatography (100% EtOAc → 14:1:1 MeCN:H_2O:sat. aq. KNO_3). To the concentrated aqueous mixture was added saturated aqueous KPF_6 (50 mL) to precipitate the desired product, and the resultant mixture was transferred to a separatory funnel with CH_2Cl_2 (50 mL). The layers were separated and the aqueous layer was extracted with CH_2Cl_2 (2 x 50 mL). The combined organic layers were then dried over Na_2SO_4, concentrated under reduced pressure and dried under reduced pressure (<1 torr) at 100 °C for 12 h to afford benzyne adduct **16** as a deep red solid (69% yield, average of two experiments). **Benzyne adduct 16**: mp >250 °C; R_f 0.68 (7:2:1 MeCN:H_2O:sat. aq. KNO_3); ^{1}H NMR (500 MHz, CD_3CN): δ 9.23 (dd, *J* = 8.4, 0.9, 2H), 8.92–8.88 (m, 2H), 8.55 (d, *J* = 8.3, 2H), 8.51 (d, *J* = 8.3, 2H), 8.11 (td, *J* = 7.9. 1.5, 2H), 8.07 (dd, *J* = 5.3, 0.9, 2H), 8.02–7.98 (m, 4H), 7.85 (d, *J* = 5.8, 2H), 7.79 (dd, *J* = 8.2, 5.3, 2H), 7.63 (d, *J* = 5.6, 2H), 7.46 (td, *J* = 6.9, 1.3, 2H), 7.23 (td, *J* = 6.9, 1.2, 2H); ^{13}C NMR (125 MHz, CD_3CN) δ 158.09, 153.11, 152.91, 152.82, 149.00, 148.82, 138.70, 133.18, 133.03, 131.32, 131.26, 131.21, 129.14, 128.34, 127.42, 127.28, 125.64, 125.60, 125.13; IR (film): 1604, 1466, 1447, 1437, 838, 761, 731, 557 cm^{-1}; HR-ESI-MS (*m/z*) $[M – PF_6]^+$ calcd for $C_{36}H_{26}F_6N_6PRu^+$, 789.09043; found 789.0905.

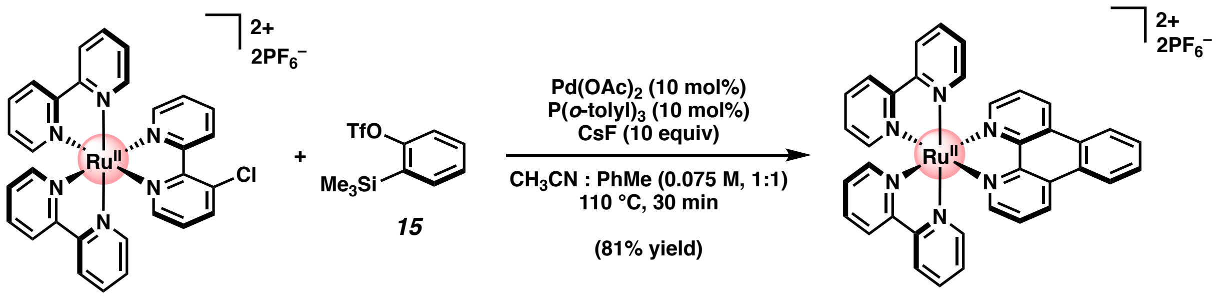

Benzyne adduct 16. Followed representative procedure A. Purification by flash chromatography (100% EtOAc → 14:1:1 CH_3CN:H_2O:sat. aq. KNO_3) afforded benzyne adduct **16** (81% yield, average of two experiments) as a red solid. Spectral data matched those provided above.

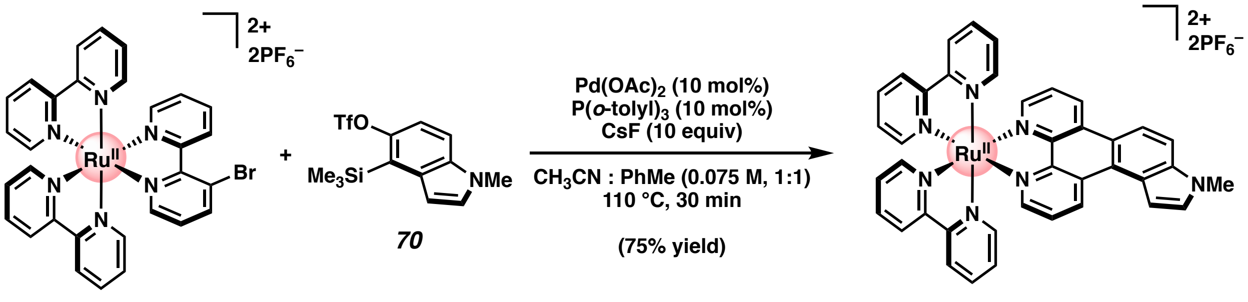

Indolyne adduct 21. Followed representative procedure A. Purification by flash chromatography (7:2:1 CH_3CN:H_2O:sat. aq. KNO_3) afforded indolyne adduct **21** (75% yield, average of two experiments) as a red solid. **Indolyne adduct 21**: mp >250 °C; R_f 0.63 (7:2:1 CH_3CN:H_2O:sat. aq. KNO_3); ^{1}H NMR (600 MHz, CD_3CN): δ 9.71 (dd, J = 8.8, 1.1, 1H), 9.27 (d, J = 8.8, 1H), 8.73 (d, J = 9.1, 1H), 8.54 (ddt, J = 8.3, 4.4, 1.1, 2H), 8.51–8.48 (m, 2H), 8.12–8.07 (m, 3H), 8.06 (dd, J = 5.2, 1.1, 1H), 8.01 (dd, J = 5.3, 1.1, 1H), 8.00–7.96 (m, 2H), 7.87–7.81 (m, 3H), 7.75 (dd, J = 8.6, 5.2, 1H), 7.67 (d, J = 3.3, 1H), 7.64–7.60 (m, 2H), 7.57 (d, J = 3.3, 1H), 7.57–7.43 (m, 2H), 7.22–7.18 (m, 2H), 4.04 (s, 3H); ^{13}C NMR (125 MHz, CD_3CN): δ 158.6, 158.5, 158.4, 153.23, 153.22, 151.71, 151.66, 149.1, 147.8, 139.1, 139.0, 138.7, 136.2, 133.6, 133.4, 132.8, 132.6, 128.92, 128.90, 128.76, 128.75, 127.6, 127.3, 125.61, 125.60, 125.5, 125.1, 123.9, 123.6, 115.2, 103.9, 34.4; IR (film): 3707, 2681, 2973, 2923, 1055, 1033; HR-ESI-MS (*m/z*) $[M - PF_6]^+$ calcd for $C_{39}H_{29}N_7P F_6Ru^+$, 842.1180; found 842.1110.

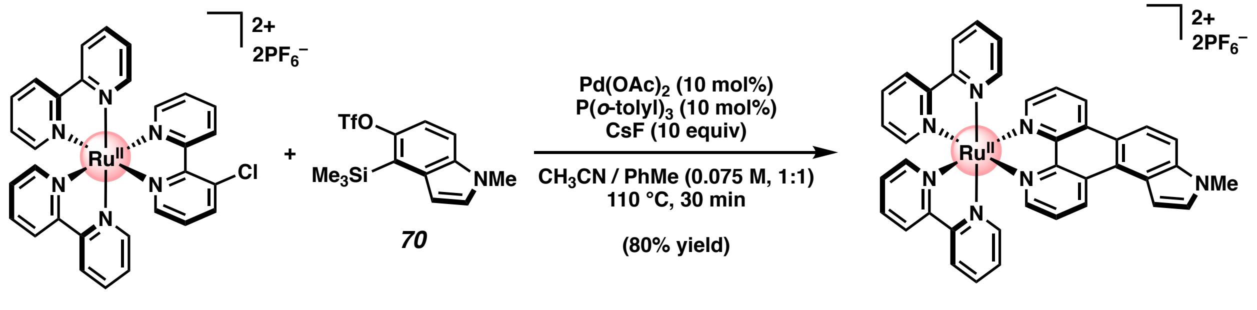

Indolyne adduct 21. Followed representative procedure A. Purification by flash chromatography (7:2:1 CH_3CN:H_2O:sat. aq. KNO_3) afforded indolyne adduct **21** (80% yield, average of two experiments) as a red solid. Spectral data matched those provided above.

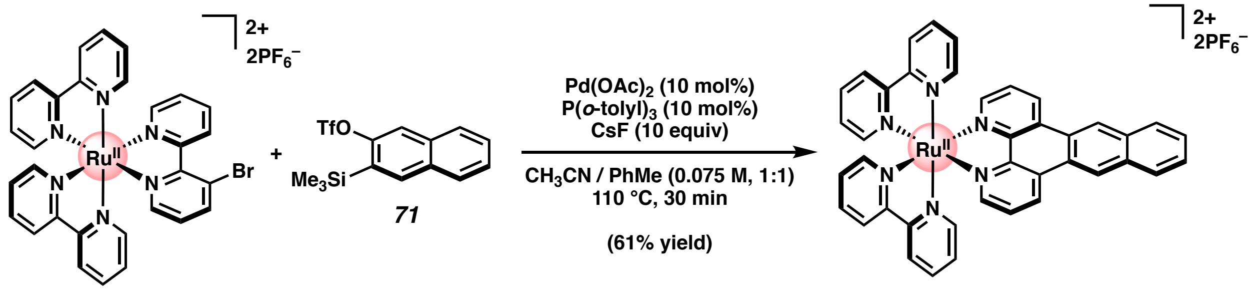

Representative Procedure B for aryne annulations (Figure 3, naphthalyne adduct 22 used as an example). To a 1-dram vial was added $Pd(OAc)_2$ (1.4 mg, 6.4 µmol, 10 mol%), Bromo-Ru(bpy)$_3$[PF$_6$]$_2$ **14a** (60.0 mg, 0.064 mmol, 1.0 equiv), P(*o*-tolyl)$_3$ (2.0 mg, 6.4 µmol, 10 mol%), MeCN (0.50 mL, 0.15 M), PhMe (0.50 mL, 0.15 M), silyl triflate **71** (44.6 mg, 0.128 mmol, 2.0 equiv), an oven-dried magnetic stir bar, and CsF (97.1 mg, 0.639 mmol, 10.0 equiv) sequentially. The reaction was then purged with N_2 for 5 min before being sealed with a Teflon-lined screw cap under a flow of N_2, sealed with Teflon tape and electrical tape, transferred to an Al-block, and stirred at 110 °C for 30 min. After cooling to 23 °C, the mixture was filtered through a plug of celite with MeCN (6 mL), and concentrated under reduced pressure. The crude residue was adsorbed onto silica gel (500 mg) under reduced pressure and purified by flash chromatography (100% EtOAc → 7:2:1 MeCN:H_2O:sat. aq. KNO_3). To the concentrated aqueous mixture was added saturated aqueous KPF_6 (50 mL) to precipitate the desired product, and the resultant mixture was transferred to a separatory funnel with CH_2Cl_2 (50 mL). The layers were separated and the aqueous layer was extracted with CH_2Cl_2 (2 x 50 mL). The combined organic layers were then concentrated under reduced pressure before being redissolved in CH_3CN (10 mL). Activated

charcoal (150 mg) was then added the mixture was agitated for 10 seconds before being filtered over celite, concentrated under reduced pressure, and dried under reduced pressure (<1 torr) at 100 °C for 12 h to afford naphthalyne adduct **22** as a deep red solid (61% yield, average of two experiments). **Naphthalyne adduct 22**: mp >250 °C; R_f 0.57 (7:2:1 CH_3CN:H_2O:saturated aqueous KNO_3); ^{1}H NMR (500 MHz, CD_3CN): δ 9.41 (s, 2H), 9.29 (dd, *J* = 8.0, 1.2, 2H), 8.56–8.51 (m, 4H), 8.30–8.27 (m, 2H), 8.11 (td, *J* = 10, 1.4, 2H), 8.03–7.99 (m, 4H), 7.85 (dd, *J* = 5.5, 0.5, 2H), 7.79–7.76 (m, 4H), 7.72 (dd, *J* = 5.6, 0.5, 2H), 7.46 (ddd, *J* = 7.8, 5.7, 1.3, 2H), 7.27 (ddd, *J* = 7.8, 5.7, 1.3, 2H); ^{13}C NMR (125 MHz, CD_3CN): δ 158.0, 157.9, 152.9, 152.8, 152.3, 149.5, 138.8, 138.6, 133.9, 133.0, 132.2, 129.3, 129.1, 128.5, 128.4, 127.7, 126.4, 125.5, 125.2, 125.1; IR (film): 3681, 2981, 2923, 1332, 1054, 1033, 1013 cm^{-1}; HR-ESI-MS (*m/z*) $[M – PF_6]^+$ calcd for $C_{40}H_{27}N_6PF_6Ru^+$, 839.1061; found 839.1050.

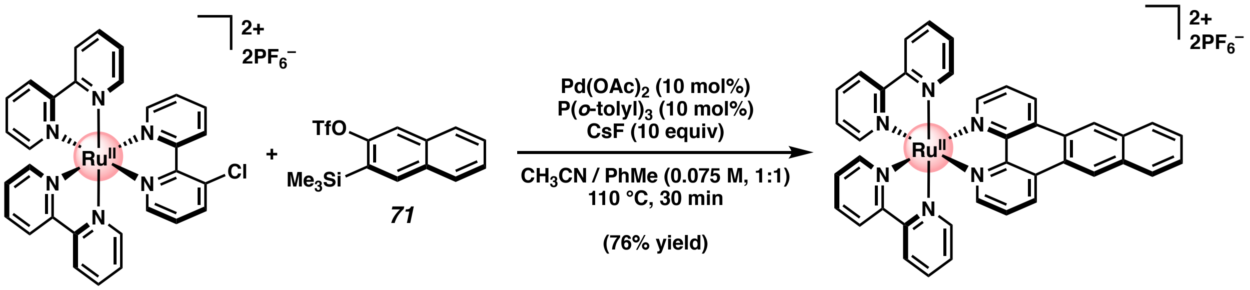

14b ***22***

Naphalyne adduct 22. Followed representative procedure B. Purification by flash chromatography (7:2:1 CH_3CN:H_2O:sat. aq. KNO_3) afforded naphthalyne adduct **22** (76% yield, average of two experiments) as a red solid. Spectral data matched those provided above.

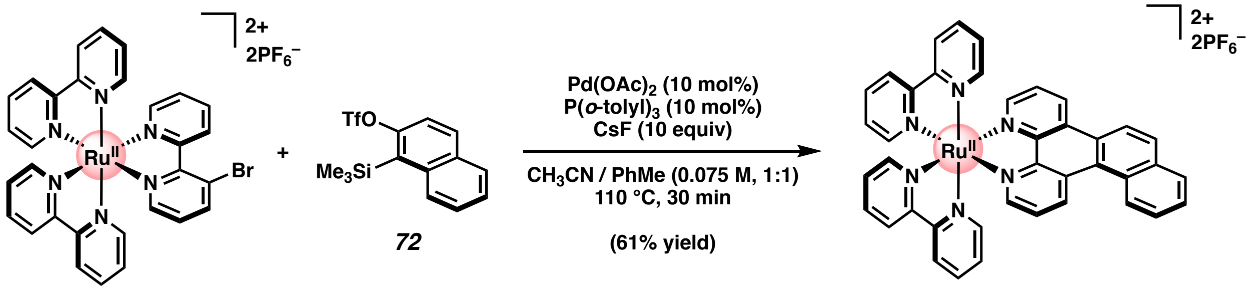

Naphthalyne adduct 23. Followed representative procedure B. Purification by flash chromatography (14:2:1 CH_3CN:H_2O:sat. aq. KNO_3) afforded naphthalyne adduct **23** (61% yield, average of two experiments) as a red solid. **Naphthalyne adduct 23**: mp >250 °C; R_f 0.70 (7:2:1 CH_3CN:H_2O:sat. aq. KNO_3); ^1H NMR (600 MHz, CD_3CN): δ 9.52 (d, J = 8.7, 1H), 9.29 (d, J = 8.7, 1H), 8.96 (d, J = 8.3, 1H), 8.82 (d, J = 8.7, 1H), 8.57–8.50 (m, 4H), 8.37 (d, J = 8.8, 1H), 8.24 (d, J = 7.9, 1H), 8.15–8.09 (m, 4H) 8.00 (t, J = 7.8, 2H), 7.89–7.78 (m, 6H), 7.69 (d, J = 5.2, 1H), 7.64 (d, J = 5.6, 1H), 7.49–7.44 (m, 2H), 7.23–7.20 (m, 2H); ^{13}C NMR (125 MHz, CD_3CN): δ 158.1, 157.9, 152.92, 152.86, 152.8, 152.0, 149.1, 148.6, 138.8, 138.7, 138.0, 135.3, 133.7, 132.0, 130.8, 130.6, 130.5, 130.0, 129.1, 128.9, 128.6, 128.5, 128.3, 127.3, 126.9, 126.3, 125.2, 125.1, 121.6; IR (film): 3663, 3589, 3084, 2925, 2854 cm^{-1}; HR-ESI-MS (*m/z*) $[M - PF_6]^+$ calcd for $C_{40}H_{27}N_6PF_6Ru^+$, 839.1061; found 839.1119.

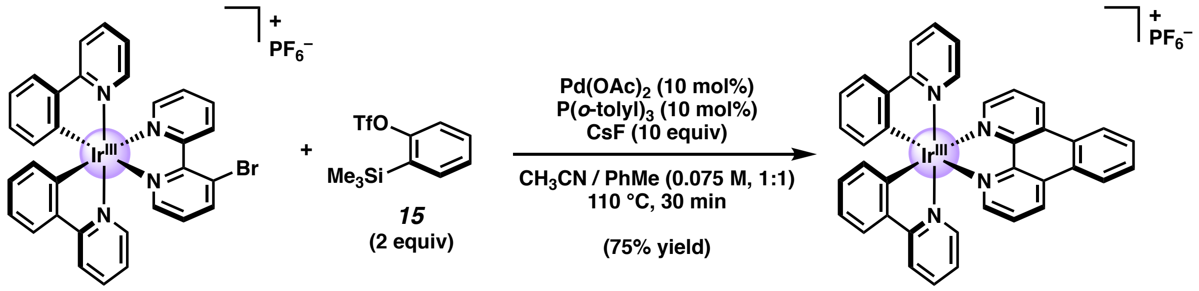

Ir-benzyne adduct 26. Followed representative procedure A. Purification by flash chromatography (9:1 benzene:acetonitrile → 3:2 benzene:acetonitrile) afforded Ir-benzyne adduct **26** (75% yield, average of two experiments) as a dark orange solid. **Ir-benzyne adduct 26**: mp >250 °C; R_f 0.59 (7:2:1 CH_3CN:H_2O:sat. aq. KNO_3); ^1H NMR (600 MHz, CD_3CN): δ 9.30 (dd, J = 8.6, 0.9, 1H), 8.92–8.88 (m, 1H), 8.29 (dd, J = 5.1, 1.3, 1H), 8.06 (d, J = 8.3, 1H), 8.00–7.98 (m, 1H), 7.89 (dd, J = 8.6, 5.1, 1H), 7.84 (dd, J = 8.0, 1.1, 1H), 7.79 (ddd, J = 8.2, 7.6, 1.5, 1H), 7.49 (ddd, J = 6.0, 1.5, 0.7, 1H), 7.29 (dd, J = 8.4, 5.3, 1H), 7.06–7.00 (m, 4H), 6.90 (tdd, J = 7.5, 2.5,

1.3, 2H), 6.22 (ddd, 7.5, 4.8, 1.2, 2H); ^{13}C NMR (125 MHz, CD_3CN): δ 168.33, 151.5, 151.2, 150.4, 147.9, 145.1, 139.3, 134.7, 132.6, 131.7, 131.3, 131.1, 129.1, 128.2, 125.8, 125.5, 124.3, 123.5, 120.7; IR (film): 2981, 1735, 1689, 1368, 1149 cm^{-1}; HR-ESI-MS (*m/z*) $[M – PF_6]^+$ calcd for $C_{38}H_{26}N_4Ir^+$, 731.1787; found 731.1704.

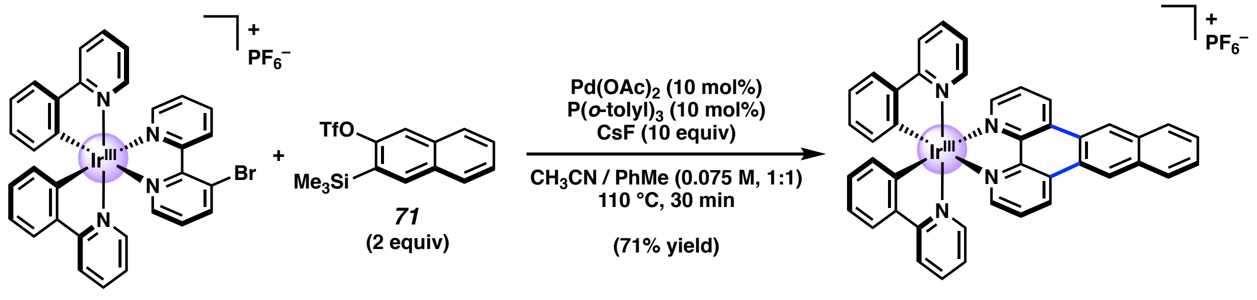

54 **27**

Ir-naphthalyne adduct 27. Followed representative procedure B. Purification by flash chromatography (100% EtOAc → 14:2:1 MeCN:H_2O:sat. aq. KNO_3) afforded naphthalyne adduct **27** as an orange solid (71% yield, average of two experiments). **Naphthalyne adduct 27**: mp >200 °C; R_f 0.70 (14:2:1 CH_3CN:H_2O:saturated aqueous KNO_3); ^{1}H NMR (500 MHz, CD_3CN): δ 9.41 (s, 2H), 9.36 (dd, J = 8.6, 1.2, 2H), 8.30–8.27 (m, 2H), 8.24 (dd, J = 5.4, 1.3, 2H), 8.07 (d, J = 8.3, 2H), 7.89–7.77 (m, 10H), 7.59 (ddd, J = 6.0, 1.5, 0.8, 2H), 7.09 (td, J = 7.2, 1.5, 2H), 6.97 (td, J = 7.7, 1.4, 2H), 6.91 (td, J = 6.8, 1.5, 2H), 6.38 (dd, J = 7.5, 0.8, 2H); ^{13}C NMR (125 MHz, CD_3CN): δ 167.8, 150.71, 150.65, 149.8, 148.1, 144.6, 138.8, 134.0, 133.4, 132.2, 132.0, 130.7, 128.7, 128.5, 128.0, 125.8, 125.2, 124.9, 123.7, 122.9, 120.2; IR (film): 3050, 2924, 2855, 1608, 1479 cm^{-1}; HR-ESI-MS (*m/z*) $[M – PF_6]^+$ calcd for $C_{42}H_{27}N_4Ir^+$, 780.1859; found 780.1835.

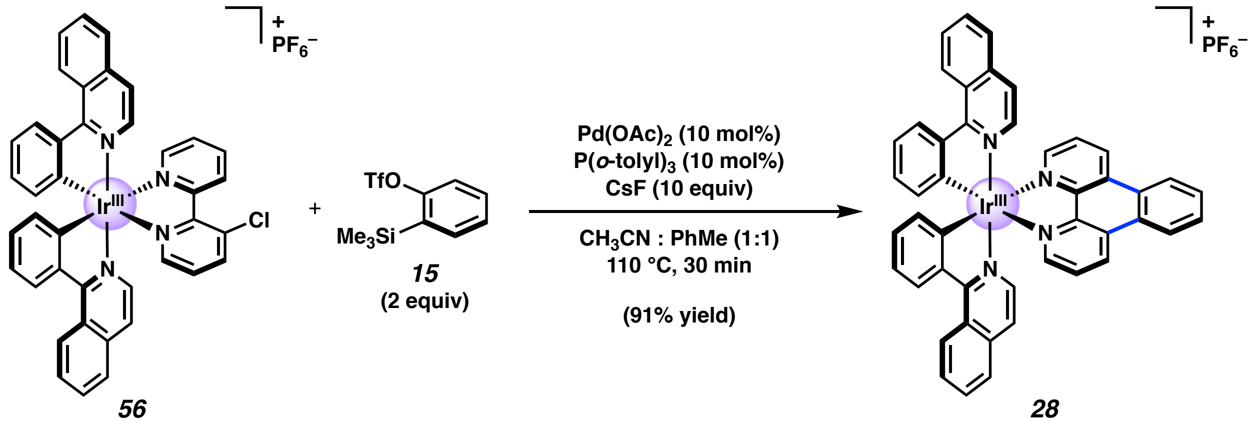

56 **28**

Isoquinolinyl adduct 28. Followed representative procedure A. Purification by flash chromatography (100% EtOAc → 14:1:1 MeCN:H_2O:sat. aq. KNO_3) afforded isoquinolinyl adduct **28** as a red solid (91% yield, average of two experiments). **Isoquinolinyl adduct 28**: mp >200 °C; R_f 0.63 (14:1:1 CH_3CN:H_2O:saturated aqueous KNO_3); ^{1}H NMR (500 MHz, CD_3CN): δ 9.30 (dd, J = 8.5, 1.3, 2H), 9.04 (d, J = 8.5, 2H), 8.92–8.84 (m, 2H), 8.42 (d, J = 8.0, 2H), 8.18 (dd, J = 5.1, 1.3, 2H), 8.01–7.97 (m, 2H), 7.93–7.90 (m, 2H), 7.87–7.78 (m, 6H), 7.40 (d, J = 6.5, 2H), 7.26 (d, J = 6.5, 2H), 7.18 (ddd, J = 8.1, 7.0, 1.3, 2H), 6.93 (td, J = 7.5, 1.2, 2H), 6.40 (dd, J = 7.6, 1.2, 2H); ^{13}C NMR (125 MHz, CD_3CN): δ 169.5, 154.6, 151.6, 147.8, 146.7, 141.9, 138.0, 134.8, 133.1, 132.8, 131.8, 131.4, 131.2, 129.9, 129.1, 128.4, 128.3, 127.7, 127.1, 125.5, 123.3, 122.7; IR (film): 3047, 1577, 1541, 1435, 841 cm^{-1}; HR-ESI-MS (*m/z*) [M – PF_6]$^+$ calcd for $C_{46}H_{30}N_4Ir^+$, 831.2100; found 831.2111.

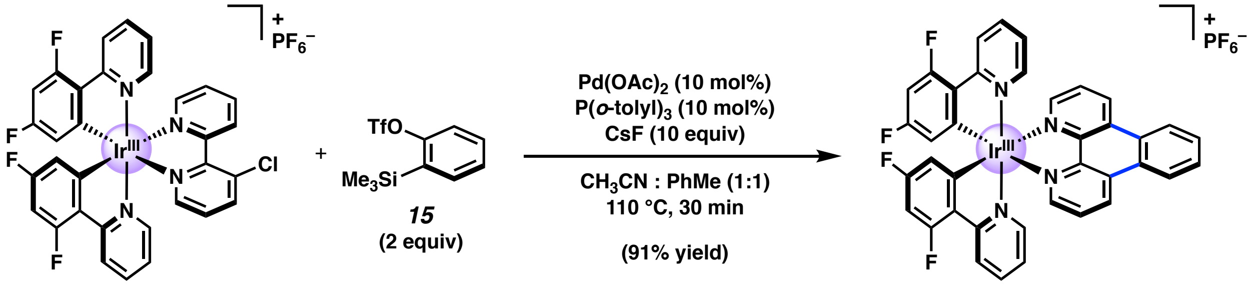

58 *29*

Fluorinated Ir adduct 29. Followed representative procedure A. Purification by flash chromatography (100% EtOAc → 14:1:1 MeCN:H_2O:sat. aq. KNO_3) afforded fluorinated Ir adduct **29** as a golden solid (91% yield, average of two experiments). **Fluorinated Ir adduct 29**: mp >200 °C; R_f 0.64 (14:1:1 CH_3CN:H_2O:saturated aqueous KNO_3); ^{1}H NMR (500 MHz, CD_3CN): δ 9.33 (dd, J = 8.6, 1.3, 2H), 8.94–8.82 (m, 2H), 8.35–8.30 (m, 4H), 8.01–7.97 (m, 2H), 7.92 (dd, J = 8.5, 5.1, 2H), 7.85 (td, J = 8.0, 1.3, 2H), 7.54 (ddd, J = 5.9, 1.6, 0.7, 2H), 6.93 (ddd, J = 7.8, 6.0, 1.4, 2H), 6.72 (ddd, J = 12.5, 9.5, 2.5, 2H), 5.84 (dd, J = 8.6, 2.4, 2H); ^{13}C NMR (125 MHz, CD_3CN): δ 165.5, 165.4, 164.7, 164.6, 163.5, 163.43, 163.40, 163.3, 161.4, 161.3, 155.33, 155.28, 151.9, 150.8, 147.6, 140.4, 135.3, 132.0, 131.3, 129.12, 129.11, 129.09, 129.05, 128.4, 125.5, 124.8, 124.7, 124.5, 114.91, 114.89, 114.77, 114.75, 99.9, 99.7, 99.5; IR (film): 3082, 1739, 1603, 1406, 1105, 840 cm^{-1}; HR-ESI-MS (*m/z*) [M – PF_6]$^+$ calcd for $C_{38}H_{22}F_4N_4Ir^+$, 803.1410; found 803.1416.

(Note: Complex splitting patterns observed due to presence of fluoride substituents; these data represent empirically observed chemical shifts from the ^{13}C NMR spectrum)

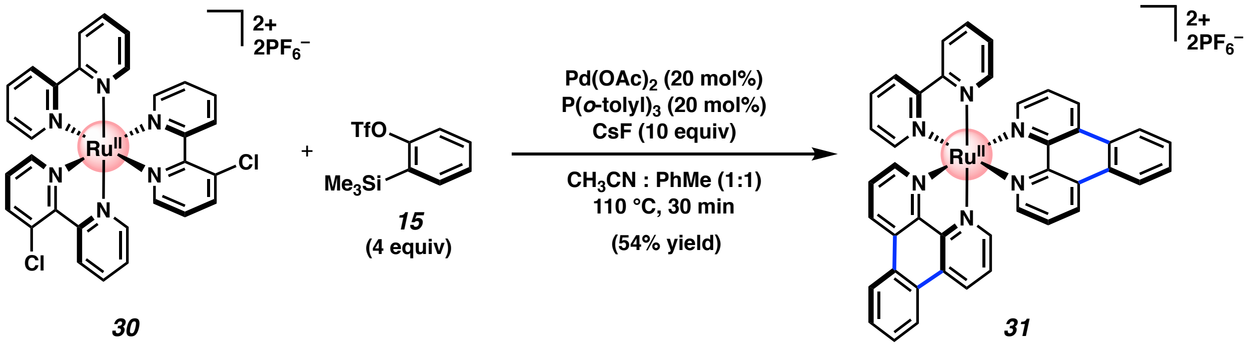

Bis(annulation) adduct 31. Followed representative procedure A. Purification by flash chromatography (100% EtOAc → 14:1:1 CH_3CN:H_2O:sat. aq. KNO_3) afforded adduct **31** (54% yield, average of two experiments) as a red solid. **Bis(annulation adduct) 31**: mp >250 °C; R*f* 0.77 (7:2:1 MeCN:H_2O:sat. aq. KNO_3); ^{1}H NMR (500 MHz, CD_3CN): δ 9.27 (d, *J* = 8.6, 1H), 9.16 (d, *J* = 8.6, 1H), 8.96–8.85 (m, 2H), 8.54 (d, *J* = 8.4, 1H), 8.17 (d, *J* = 5.2, 1H), 8.07–7.92 (m, 4H), 7.83 (dd, *J* = 8.5, 5.3, 1H), 7.74 (d, *J* = 5.3, 1H), 7.61 (dd, *J* = 8.2, 5.4, 1H), 7.28 (t, *J* = 6.4, 1H); ^{13}C NMR (125 MHz, CD_3CN, 19 of 21 signals observed) δ 158.1, 153.1, 152.9, 152.8, 149.0, 148.8, 138.7, 133.2, 133.0, 131.32, 131.26, 131.2, 129.14, 128.3, 127.4, 127.3, 125.64, 125.60, 125.1; IR (film): 1467, 1436, 1407, 840, 762 cm^{-1}; HR-ESI-MS (*m/z*) [M – PF_6]$^+$ calcd for $C_{42}H_{28}N_6F_6PRu^+$, 863.10662; found 863.1080.

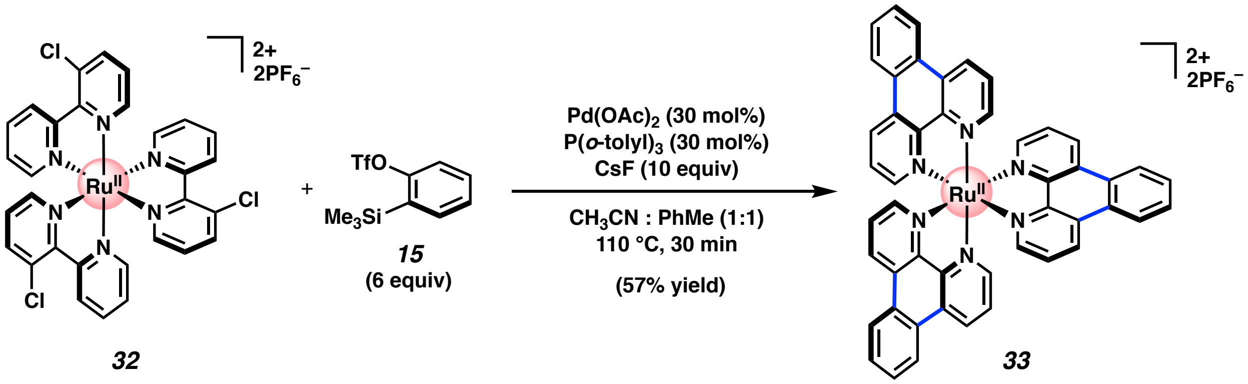

(Tris)annulation adduct 33. Followed representative procedure B. Purification by flash chromatography (100% EtOAc → 14:1:1 CH_3CN:H_2O:sat. aq. KNO_3) afforded adduct **33** (57%

yield, average of two experiments) as a red solid. (**Tris)annulation adduct 33**: mp >250 °C; R_f 0.78 (7:2:1 CH_3CN:H_2O:sat. aq. KNO_3); ^{1}H NMR (500 MHz, CD_3CN): δ 9.22 (dd, *J* = 8.7, 0.7, 6H), 8.93–8.90 (m, 6H), 8.08 (dd, *J* = 5.3, 1.0, 6H), 8.03–7.99 (m, 6H), 7.68 (dd, *J* = 8.3, 5.3, 6H); ^{13}C NMR (125 MHz, CD_3CN) δ 153.1, 149.0, 133.1, 131.3, 131.2, 129.2, 127.3, 125.6; IR (film): 2962, 1435, 1407, 1260, 1211, 1084, 1021, 840, 805, 758, 557; HR-ESI-MS (*m/z*) $[M - PF_6]^+$ calcd for $C_{48}H_{30}F_6N_6PRu^+$, 937.12227; found 937.1227.

F. Synthesis and Trapping Experiments of Ru-Centered Aryne

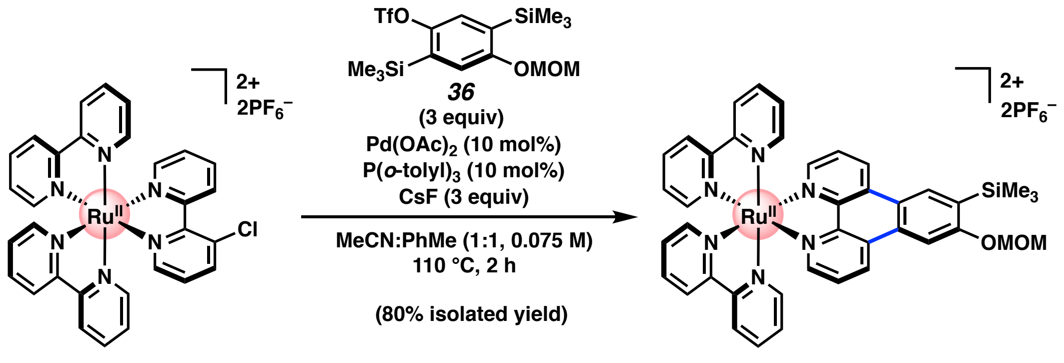

Methoxymethyl ether adduct 37. To a 20 mL scintillation vial was added $Pd(OAc)_2$ (10.1 mg, 44.8 µmol, 10 mol%), chloro-$Ru(bpy)_3[PF_6]_2$ **14b** (400.5 mg, 0.448 mmol, 1.0 equiv), P(*o*-tolyl)$_3$ (13.6 mg, 44.8 mmol, 10 mol%), MeCN (3.0 mL), PhMe (3.0 mL), silyl triflate **36** (564 mg, 1.31 mmol, 3.0 equiv), an oven-dried magnetic stir bar, and CsF (204 mg, 1.34 mmol, 3.0 equiv) sequentially. The reaction was then purged with N_2 for 5 min before being sealed with a Teflon-lined screw cap under a flow of N_2, sealed with Teflon tape and electrical tape, transferred to an Al-block, and stirred at 110 °C for 2 h. After cooling to 23 °C, the mixture was filtered through a plug of celite with MeCN (10 mL), and concentrated under reduced pressure. The crude residue was adsorbed onto SiO_2 and purified by flash chromatography (100% EtOAc → 14:1:1 MeCN:H_2O:sat. aq. KNO_3). To the concentrated aqueous mixture was added saturated aqueous KPF_6 (100 mL) to precipitate the desired product, and the resultant mixture was transferred to a separatory funnel with CH_2Cl_2 (50 mL). The layers were separated and the aqueous layer was extracted with CH_2Cl_2 (2 x 50 mL). The combined organic layers were then concentrated under reduced pressure before being redissolved in CH_3CN (10 mL). Activated charcoal (500 mg) was then added and the mixture was agitated for 10 seconds before being filtered over celite,

concentrated under reduced pressure, and dried under reduced pressure (<1 torr) at 100 °C for 12 h to afford methoxymethyl ether adduct **37** as a red solid (380 mg, 80% yield). **Methoxymethyl ether adduct 37**: mp >250 °C; R_f 0.79 (7:2:1 CH_3CN:H_2O:saturated aqueous KNO_3); ^{1}H NMR (500 MHz, CD_3CN): δ 9.25 (dd, *J* = 12.0, 8.5, 2H), 8.88 (s, 1H), 8.60 (dd, *J* = 8.2, 2H), 8.56 (dd, *J* = 8.2, 2H), 8.34 (s, 1H), 8.16–8.11 (m, 3H), 8.08 (d, *J* = 5.2, 1H), 8.03 (tt, *J* = 7.9, 1.7, 2H), 7.91 (d, *J* = 5.8, 2H), 7.82 (td, *J* = 8.4, 5.3, 2H), 7.69 (t, *J* = 6.8, 2H), 7.50 (td, *J* = 6.6, 1.0, 2H), 7.28 (dd, *J* = 12.9, 5.5, 2H), 5.58 (q, *J* = 5.5, 2H), 3.54 (s, 3H), 0.48 (s, 9H); ^{13}C NMR (100 MHz, CD_3CN) δ 163.8, 158.1, 157.9, 152.9, 152.84, 152.79, 152.8, 152.7, 151.6, 149.2, 147.7, 138.7, 138.6, 134.4, 133.2, 132.7, 132.6, 131.8, 131.2, 130.8, 128.5, 128.3, 128.3, 127.3, 127.1, 125.2, 125.1, 122.6, 106.7, 94.9, 57.0, 55.2, -1.02; IR (film): 3464, 3079, 1604, 1369, 1345 cm^{-1}; HR-ESI-MS (*m/z*) $[M - PF_6]^+$ calcd for $C_{41}H_{38}F_6N_6O_2PRuSi^+$, 921.15163; found 921.1541.

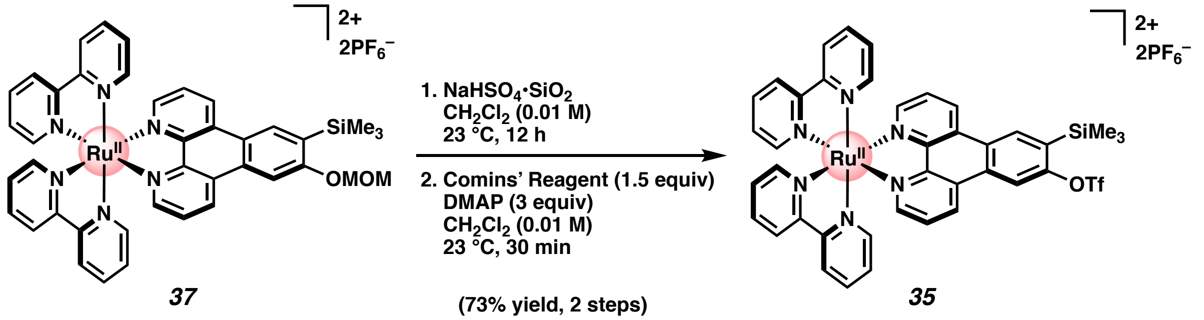

Silyl triflate 35. To a stirred solution of methoxymethyl ether **37** (259 mg, 0.243 mmol, 1.0 equiv) in CH_2Cl_2 (25 mL, 0.01 M) at 23 °C was added solid supported catalyst[5] $NaHSO_4$•SiO_2 (2.63 g, stored in the oven for >48 h and added while hot) in one portion. The reaction mixture was then stirred for 6 h at 23 °C. The mixture was then filtered through a plug of silica gel with 7:2:1 MeCN:H_2O:sat. aq. KNO_3 (100 mL). The eluate was concentrated under reduced pressure before being crashed out with sat. aq. KPF_6 (100 mL) and transferred to a separatory funnel with CH_2Cl_2 (100 mL). The layers were separated and the aqueous layer was extracted with CH_2Cl_2 (2 x 100 mL). The combined organic layers were then concentrated under reduced pressure to afford the intermediate silyl alcohol as a red solid.

The intermediate silyl alcohol was then dissolved in CH_2Cl_2 (25 mL, 0.01 M) and while stirring at 23 °C, DMAP (89.3 mg, 0.731 mmol, 3.0 equiv) and Comins' reagent (144 mg, 0.366 mmol, 1.5 equiv) were added sequentially, each in single portions. After stirring at 23 °C for 30 min, the reaction was concentrated under reduced pressure and then loaded onto a silica plug. The

silica plug was washed with EtOAc (200 mL; eluate discarded) before being eluted with 7:2:1 MeCN:H_2O:sat. aq. KNO_3 (100 mL). The red eluate was then concentrated under reduced pressure and transferred with CH_2Cl_2 (100 mL) to a separatory funnel containing aqueous HCl (2.0 M, 100 mL). The layers were separated and the organic layer was washed with aqueous HCl (2.0 M, 2 x 40 mL). The combined aqueous layers were then extracted with CH_2Cl_2 (2 x 100 mL). The organic layers were then combined, dried over Na_2SO_4, and concentrated under reduced pressure. The residue was then filtered over a pad of neutral alumina (200 mL CH_3CN eluent) and concentrated under reduced pressure before being dried under reduced pressure (<1 torr) at 100 °C for 1 h to afford silyl triflate **35** as a red solid (204 mg, 73% yield). **Silyl triflate 35**: mp: >250 °C; R_f 0.34 (14:1:1 MeCN:H_2O:sat. aq. KNO_3); ^{1}H NMR (500 MHz, CD_3CN): δ 9.29 (d, J = 8.6, 1H), 9.11 (d, J = 8.5, 1H), 9.02 (s, 1H), 8.69 (s, 1H), 8.53 (d, J = 8.2, 2H), 8.49 (d, J = 8.2, 2H), 8.13–8.08 (m, 4H), 8.00 (tt, J = 7.9, 1.7, 2H), 7.84–7.78 (m, 4H), 7.60 (t, J = 5.3, 2H), 7.45 (t, J = 6.8, 2H), 7.22 (qt, J = 6.6, 1.3, 2H), 0.55 (s, 9H); ^{13}C NMR (125 MHz, CD_3CN) δ 158.1, 157.9, 156.2, 153.7, 153.3, 153.00, 152.97, 152.9, 152.8, 149.5, 149.1, 138.9, 138.7, 136.7, 134.7, 133.6, 133.2, 131.8, 130.3, 130.1, 128.5, 128.4, 128.0, 127.7, 125.24, 125.16, 116.1; IR (film): 3359, 3310, 3193, 2920, 2851, 1632 cm^{-1}; HR-ESI-MS (m/z) [M – PF_6]$^+$ calcd for $C_{40}H_{33}F_9N_6O_3PRuSSi^+$, 1009.07470; found 1009.0704.

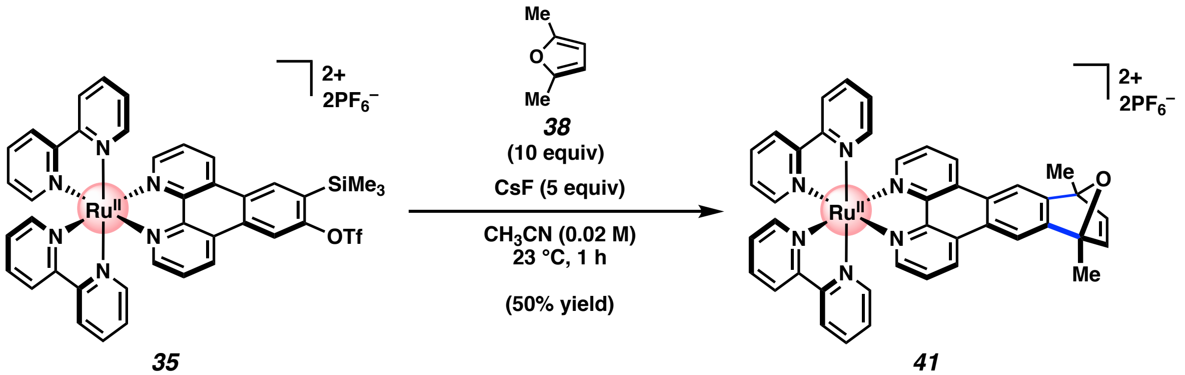

Cycloadduct 41. To a 1-dram vial was added silyl triflate **35** (19.5 mg, 16.9 μmol) and dissolved in MeCN (0.8 mL, 0.02 M). 2,5-dimethylfuran (**38**) (16.2 mg, 0.17 mmol, 10 equiv) was then added in one portion. While stirring, CsF (12.8 mg, 0.085 mmol, 5 equiv) was added in one portion and the reaction was stirred at 23 ^{a}C for 1 h. After 1 h, the reaction mixture was then filtered through a plug of celite with MeCN (10 mL), adsorbed onto silica gel (500 mg) under reduced pressure, and purified by flash chromatography (100% EtOAc → 14:1:1 MeCN:H_2O:sat. aq.

KNO_3). To the resultant concentrated aqueous mixture was added saturated aqueous KPF_6 (50 mL) to precipitate the desired product, and the resultant mixture was transferred to a separatory funnel with CH_2Cl_2 (50 mL). The layers were separated and the aqueous layer was extracted with CH_2Cl_2 (2 x 50 mL). The combined organic layers were then concentrated under reduced pressure, dried over sodium sulfate, and dried under reduced pressure (<1 torr) at 100 °C for 12 h to afford cycloadduct **41** as a red solid (4.1 mg, 25% yield). **Cycloadduct 41**: mp: >250 °C; R_f 0.31 (14:1:1 MeCN:H_2O:sat. aq. KNO_3); ^1H NMR (500 MHz, CD_3CN): δ 9.23 (dt, *J* = 8.6, 1.3, 2H), 8.59 (d, *J* = 1.4, 2H), 8.52 (dq, *J* = 8.3, 0.8, 2H), 8.49 (ddt, *J* = 8.3, 3.0, 1.1, 2H), 8.09 (td, *J* = 8.0, 1.4, 2H), 8.04 (ddd, *J* = 5.2, 4.3, 1.1, 2H), 8.01–7.94 (m, 2H), 7.83 (dddd, *J* = 5.6, 3.3, 1.5, 0.8, 2H), 7.76 (dd, *J* = 8.6, 5.2, 2H), 7.57 (dddd, *J* = 11.8, 5.7, 1.6, 0.7, 2H), 7.44 (ddt, *J* = 7.7, 5.6, 1.3, 2H), 7.19 (dddd, *J* = 11.1, 7.8, 5.8, 1.3, 2H), 6.92 (app dd, *J* = 5.4, 3.4, 2H), 2.044 (s, 3H), 2.037 (s, 3H); ^{13}C NMR (125 MHz, CD_3CN): δ 158.1, 157.90. 157.88, 156.2, 152.90, 152.89, 152.8, 152.3, 148.6, 147.41, 147.38, 138.8, 138.62, 138.59, 133.3, 131.3, 128.5, 128.33, 128.32, 127.2, 127.1, 125.2, 125.11, 125.09, 114.9, 114.8, 89.6; IR (film): 2931, 1467, 1447, 1390, 1141, 839, 762, 558 cm^{-1}; HR-ESI-MS (*m/z*) $[M – PF_6]^+$ calcd for $C_{42}H_{32}F_6N_6OPRu^+$, 883.13284; found 883.1326.

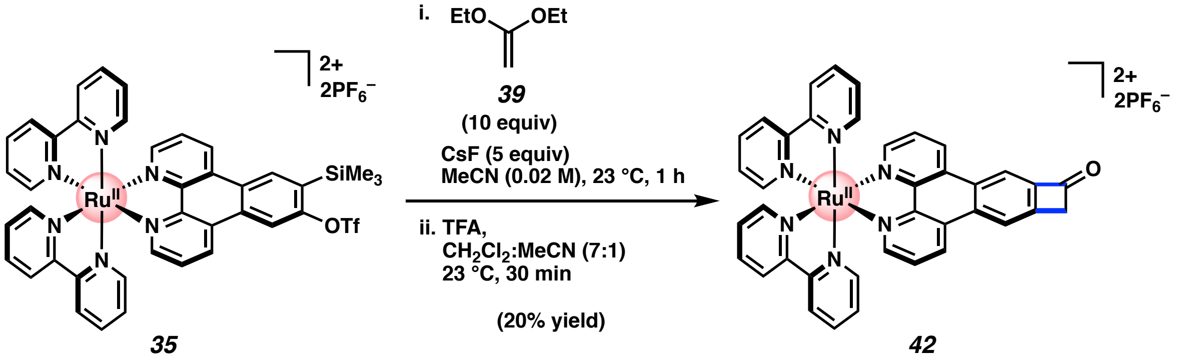

Cyclobutanone 42. To a stirred solution of silyl triflate **35** (20.5 mg, 17.8 µmol, 1.0 equiv) and 1,1-diethoxyethene (**39**) (24.9 µL, 178 µmol, 10.0 equiv) in MeCN (0.9 mL, 0.02 M) at 23 °C was added CsF (13.5 mg, 88.8 µmol, 5.0 equiv) in one portion. After stirring at 23 °C for 1 h, the reaction was filtered through a plug of celite with MeCN (10 mL) and then concentrated under reduced pressure. To the resulting residue was added CH_2Cl_2 (2.1 mL) and MeCN (0.3 mL) and while stirring at 23 °C, TFA (0.5 mL) was added dropwise over 20 seconds and the mixture was stirred at 23 °C for 30 min. The reaction was then quenched by slow addition of saturated aqueous

$NaHCO_3$ (2.5 mL) over 30 seconds. The reaction was then transferred to a separatory funnel with CH_2Cl_2 (25 mL) and saturated aqueous KPF_6 (20 mL). The layers were separated and the aqueous layer was extracted with CH_2Cl_2 (3 x 50 mL). The combined organic layers were then dried over Na_2SO_4, filtered, and concentrated under reduced pressure. Purification by preparative TLC (14:1:1 MeCN:H_2O:sat. aq. KNO_3) afforded cyclobutanone **42** as a red solid (3.4 mg, 20% yield).

Cyclobutanone 42: mp >250 °C; R_f 0.34 (14:1:1 MeCN:H_2O:sat. aq. KNO_3); ^{1}H NMR (500 MHz, CD_3CN): δ 9.29 (d, J = 8.4, 1H), 9.21 (d, J = 8.4, 1H), 9.07 (s, 1H), 8.89 (s, 1H), 8.53 (d, J = 8.4, 2H), 8.50 (d, J = 8.4, 2H), 8.14–8.05 (m, 4H), 8.00 (t, J = 8.2, 2H), 7.84–7.76 (m, 4H), 7.62 (d, J = 5.0, 2H), 7.45 (t, J = 6.4, 2H), 7.23 (t, J = 6.0, 2H), 4.37 (s, 2H); ^{13}C NMR (125 MHz, CD_3CN): δ 189.2, 158.1, 157.9, 153.7, 153.1, 152.99, 152.95, 152.83, 152.81, 151.7, 150.7, 149.7, 148.7, 138.9, 138.7, 134.7, 134.1, 133.3, 131.9, 131.1, 130.1, 128.5, 128.4, 127.63, 127.58, 125.23, 125.16, 120.5, 117.3; IR (film): 2923, 1772, 1618, 1469, 1449, 839, 558 cm^{-1}; HR-ESI-MS (m/z) $[M - PF_6]^+$ calcd for $C_{38}H_{26}F_6N_6OPRu^+$, 829.08589; found 829.0845.

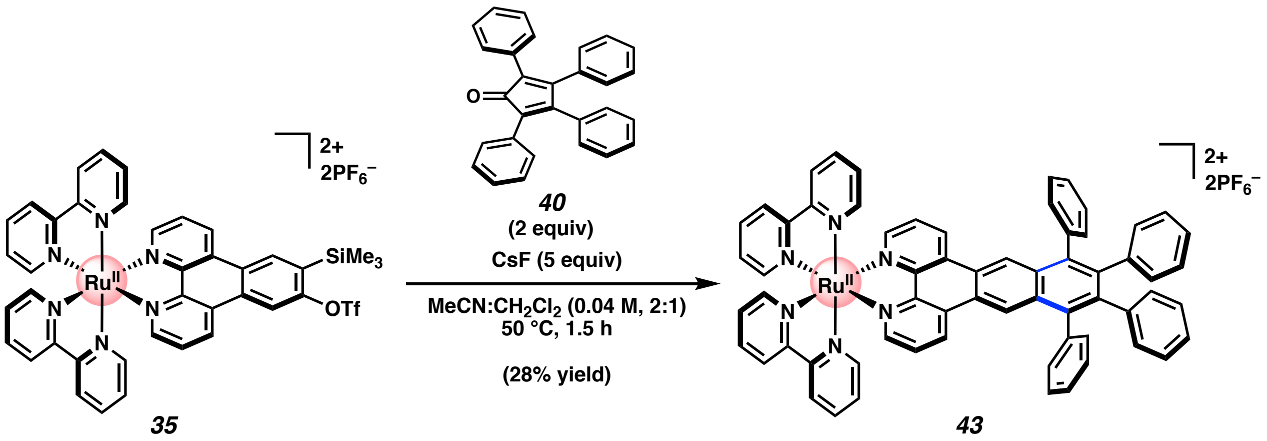

Cycloadduct 43. To a 1-dram vial was added silyl triflate **35** (99.7 mg, 86.4 µmol, 1.0 equiv), tetraphenylcyclopentadienone (**40**, 66.4 mg, 173 µmol, 2.0 equiv), MeCN (1.4 mL, 0.06 M), CH_2Cl_2 (0.7 mL, 0.12 M), and CsF (65.6 mg, 432 µmol, 5.0 equiv) were added sequentially. The reaction vessel was purged with N_2, sealed with a Teflon cap, and placed in a preheated, 50 °C aluminum heating block. After stirring for 1.5 h, the reaction was cooled to 23 °C. The reaction mixture was then filtered through a plug of celite with MeCN (10 mL), adsorbed onto silica gel (750 mg) under reduced pressure, and purified by flash chromatography (100% EtOAc → 14:1:1 MeCN:H_2O:sat. aq. KNO_3). To the concentrated aqueous mixture was added saturated aqueous

KPF_6 (50 mL) to precipitate the desired product, and the resultant mixture was transferred to a separatory funnel with CH_2Cl_2 (50 mL). The layers were separated and the aqueous layer was extracted with CH_2Cl_2 (1 x 50 mL). The combined organic layers were then dried over sodium sulfate, filtered, concentrated under reduced pressure, and dried under reduced pressure (<1 torr) at 100 °C for 12 h to afford cycloadduct **43** as a red solid (30.7 mg, 28% yield). **Cycloadduct 43**: mp >250 °C; R_f 0.84 (7:2:1 MeCN:H_2O:sat. aq. KNO_3); ^1H NMR (600 MHz, CD_3CN): δ 8.94 (s, 2H), 8.60 (dd, *J* = 8.6, 1.1, 2H), 8.51 (dt, *J* = 8.2, 1.0, 2H), 8.48 (dt, *J* = 8.2, 1.0, 2H), 8.08 (td, *J* = 8.0, 1.4, 2H), 8.00 (td, *J* = 7.9, 1.5, 2H), 7.94 (dd, *J* = 5.3, 1.1, 2H), 7.79 (ddd, *J* = 5.6, 1.4, 0.6, 2H), 7.64 (ddd, *J* = 5.7, 1.4, 0.6, 2H), 7.60 (dd, *J* = 8.3, 5.4, 2H), 7.44–7.34 (m, 12H), 7.23 (ddd, *J* = 7.7, 5.8, 1.3, 2H), 7.06–6.97 (m, 4H), 6.96–6.89 (m, 6H); ^{13}C NMR (125 MHz, CD_3CN): δ 158.0, 157.9, 152.8, 152.7, 152.4, 149.6, 142.2, 141.1, 140.0, 139.5, 138.8, 138.7, 133.0, 132.6, 132.24, 132.18, 132.00, 131.98, 131.8, 128.91, 128.88, 128.5, 128.4, 128.2, 127.8, 127.6, 126.8, 126.2, 125.2, 125.1, 124.2; IR (film): 2929, 1606, 1469, 1447, 840, 763, 701, 558 cm^{-1}; HR-ESI-MS (*m/z*) [M – PF_6]$^+$ calcd for $C_{64}H_{44}F_6N_6PRu^+$, 1143.23182; found 1443.2338.

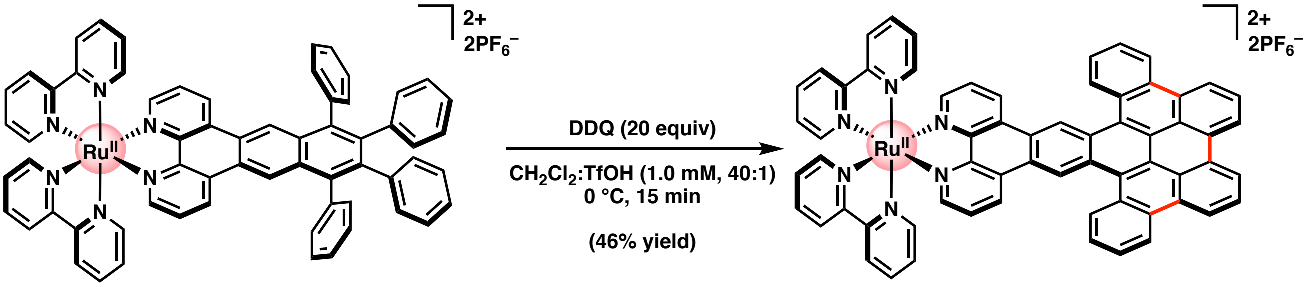

43 *44*

Scholl Product 44. To a stirred solution of **43** (6.3 mg, 4.9 µmol, 1.0 equiv), in CH_2Cl_2 (4.0 mL) at 0 °C was added DDQ (22 mg, 98 µmol, 20 equiv) in one portion. Then, TfOH (0.1 mL) was added dropwise over 1 min, whereupon the reaction turned deep green in color. The reaction was then stirred at 0 °C for 15 min, followed by addition of saturated aqueous $NaHCO_3$ (4.0 mL) slowly, over 10 seconds. The resulting mixture was then stirred at 0 °C for 5 min before being allowed to warm to 23 °C. It was then transferred with CH_2Cl_2 (30 mL) to a separatory funnel containing saturated aqueous KPF_6 (30 mL). The layers were separated and the aqueous layer was extracted with CH_2Cl_2 (2 x 30 mL). The combined organic layers were then concentrated under reduced pressure and loaded onto a silica plug, which was washed with EtOAc (25 mL) before being eluted with 7:2:1 MeCN:H_2O:sat. aq. KNO_3 (30 mL). The red eluate was then concentrated

under reduced pressure, saturated aqueous KPF_6 (30 mL) was added to precipitate the desired product, and the resultant mixture was transferred to a separatory funnel with CH_2Cl_2 (30 mL). The layers were separated and the aqueous layer was extracted with CH_2Cl_2 (1 x 30 mL). The combined organic layers were then concentrated under reduced pressure, dried over Na_2SO_4, filtered, and concentrated under reduced pressure to afford **Scholl Product 44** as a red solid (2.9 mg, 46% yield). **Scholl Product 44**: R_f 0.41 (14:1:1 MeCN:H_2O:sat. aq. KNO_3); ^{1}H NMR (500 MHz, CD_3CN): δ 10.03 (s, 2H), 9.05 (d, *J* = 8.3, 2H), 8.75 (d, *J* = 8.3, 2H), 8.71–8.64 (m, 4H), 8.61 (d, *J* = 8.2, 2H), 8.57 (d, *J* = 8.2, 2H), 8.50 (d, *J* = 7.8, 2H), 8.16 (td, *J* = 8.1, 1.4, 2H), 8.08 (dd, *J* = 5.3, 0.8, 2H), 8.05 (td, *J* = 8.0, 1.4, 2H), 7.91 (d, *J* = 5.5, 2H), 7.87–7.81 (m, 4H), 7.75 (t, *J* = 7.8, 2H), 7.69 (t, *J* = 7.6, 2H), 7.61 (t, *J* = 7.4, 2H), 7.51 (ddd, *J* = 7.8, 5.5, 1.3, 2H), 7.35 (t, *J* = 6.8, 2H); ^{13}C NMR (125 MHz, CD_3CN, 31 of 32 signals observed): δ 157.20, 157.19, 152.0, 151.9, 151.5, 148.6, 138.0, 137.9, 132.2, 131.0, 130.9, 129.9, 129.5, 129.1, 128.7, 127.9, 127.7, 127.6, 127.41, 127.37, 126.9, 124.6, 124.5, 124.42, 124.39, 124.3, 124.0, 123.3, 122.9, 122.2, 122.0; IR (film): 2925, 1468, 1447, 1425, 841, 761, 558 cm^{-1}; HR-ESI-MS (*m/z*) $[M - PF_6]^+$ calcd for $C_{64}H_{44}F_6N_6PRu^+$, 1143.23182; found 1143.2338.

*Note: **44** readily decomposes upon removal of solvent, and must therefore be carefully handled as a dilute solution. We surmise that its instability may be a consequence of its highly π-expansive nature, which introduces numerous sites for oxidation or dimerization.*

Photophysical Data

UV–vis absorption spectra were recorded on an Ocean Optics Flame-T spectrometer with the OceanView software package. Dynamic light scattering (DLS) data were collected using a Beckman-Coulter N4 Plus particle analyzer. Quartz cuvettes (1 cm) were used for absorbance and photoluminescence measurements. Relative quantum yields were determined in degassed acetonitrile relative to $Ru(bpy)_3[PF_6]_2$.

Nanosecond transient absorption experiments were performed using an Edinburgh Instruments LP920 laser flash photolysis spectrometer in conjunction with a Q-switched Nd:YAG Brilliant b laser from Quantel with a 266 nm output wavelength, a 5−8 ns pulse width, a 1 Hz repetition rate, and a 36−40 mJ pulse energy. Transient absorption detection is based on a 450 W pulsed xenon arc lamp, a Czerny-Turner TMS300 monochromator, a Hamamatsu R928 photomultiplier detector, and a Tektronix TDS3012C digital oscilloscope. Transient absorption data were collected and processed using the L900 software package provided by Edinburgh Instruments. Samples were prepared as solutions in acetonitrile and degassed by sparging with argon in a quartz cuvette. Reported transient absorption lifetime (τ_{TA}) values are reported as the average of three experiments. Phosphorescence spectra were recorded using an Edinburgh Instruments FLSP920 fluorimeter with a 450 W xenon lamp in CW (continuous wave) emission source.

A. Compiled Photophysical Data Table

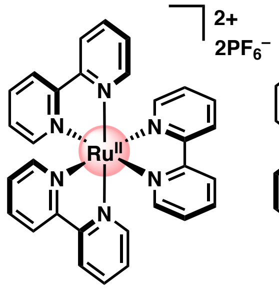

17

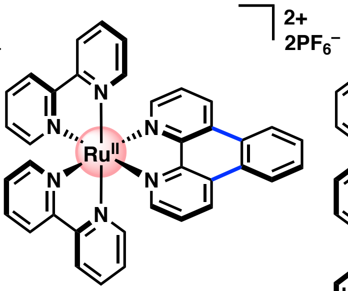

16

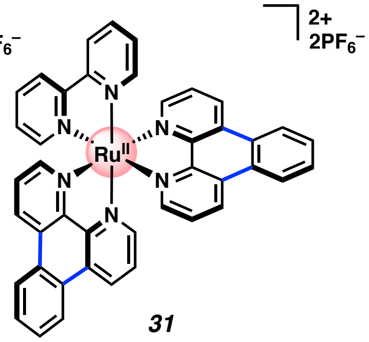

31

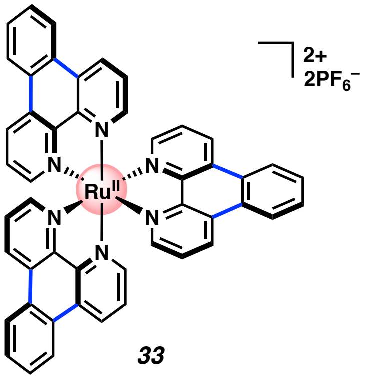

33

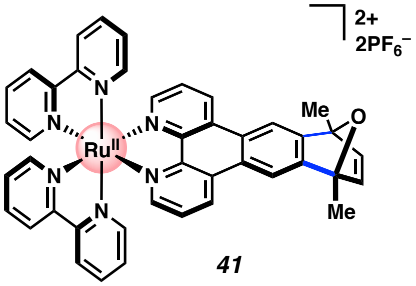

41

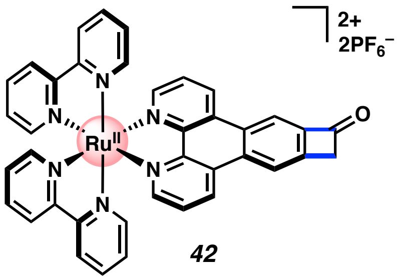

42

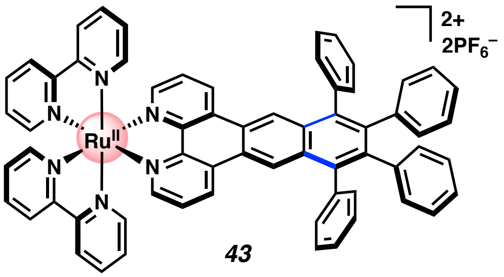

43

Ru Complex	$\lambda_{\text{max em.}}$ (nm)	$\varepsilon \times 10^3$ (mol^{-1} cm^{-1}; at 452 nm)	Φ_P (%)	τ_{TA} (ns)
17	593	18.1	9.5 (lit.)[6]	1115
16	592	7.7	15	1077
31	589	18.2	15	1382
33	586	23.5	24	1479
41	592	14.7	14	1100
42	594	11.5	14	1339
43	591	15.8	16	2006

Supplementary Table 3: Photophysical data for selected Ru complexes. Samples were prepared as solutions in acetonitrile and degassed by sparging with argon in a quartz cuvette.

B. UV–Vis Spectra

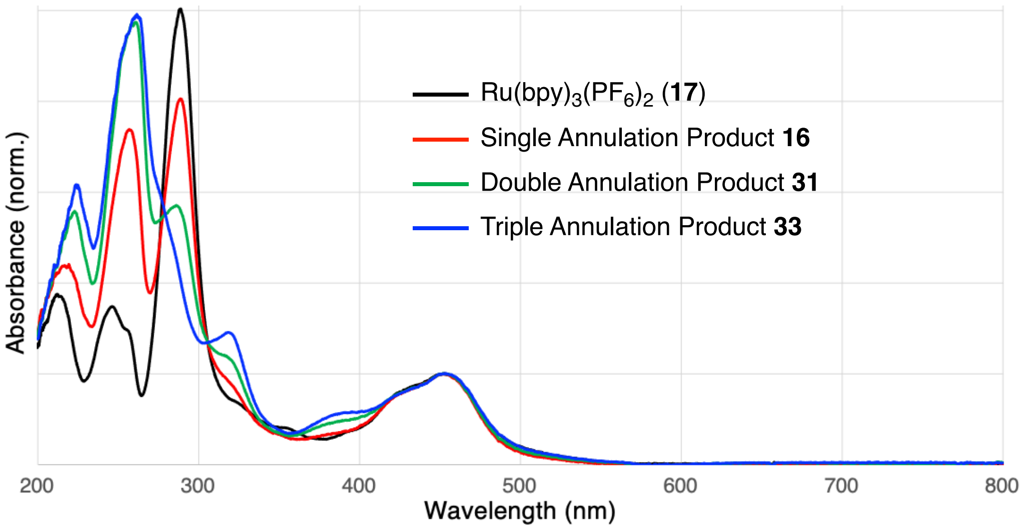

Supplementary Figure 3. UV−vis absorption spectra of compounds **17**, **16**, **31**, and **33**. Spectra are normalized to OD = 1 at the MLCT maximum (452 nm) to compare the band shapes.

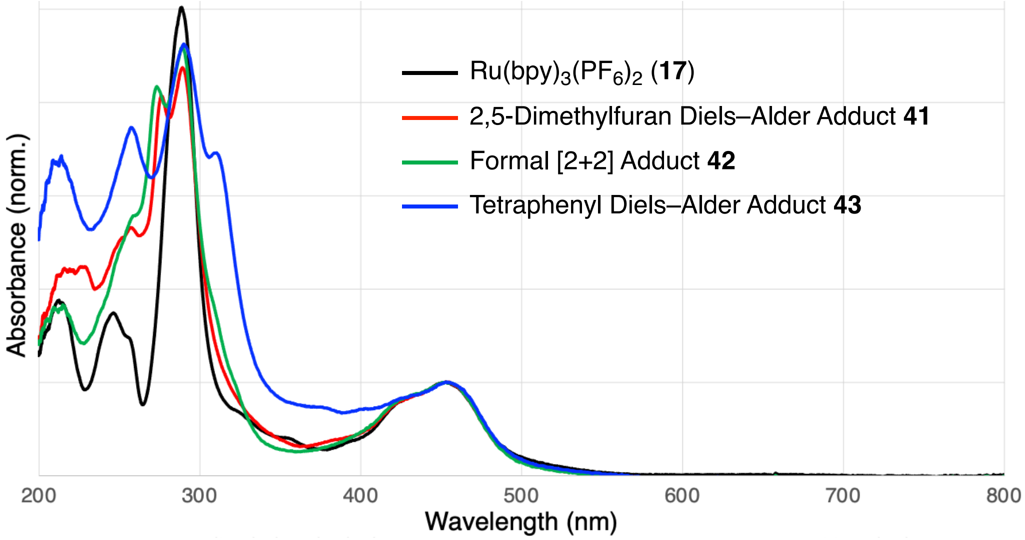

Supplementary Figure 4. UV−vis absorption spectra of compounds **17**, **41**, **42**, and **43**. Spectra are normalized to OD = 1 at the MLCT maximum (452 nm) to compare the band shapes.

C. Molar Extinction Coefficient Measurements

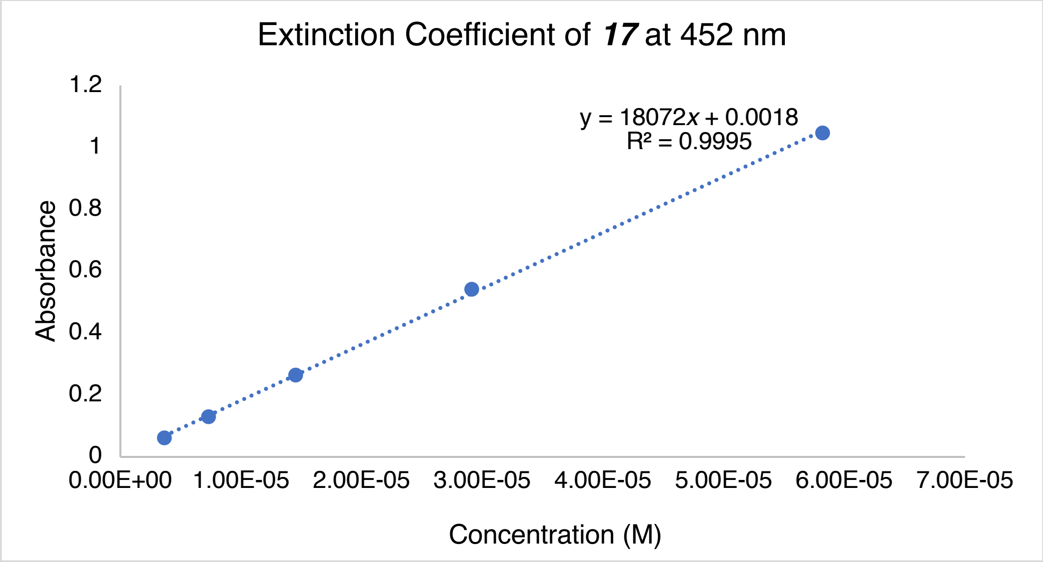

Supplementary Figure 5. Beer-Lambert plot of **17** at 452 nm. (ε = 18.1 x 10^4 M^{-1} cm^{-1}).

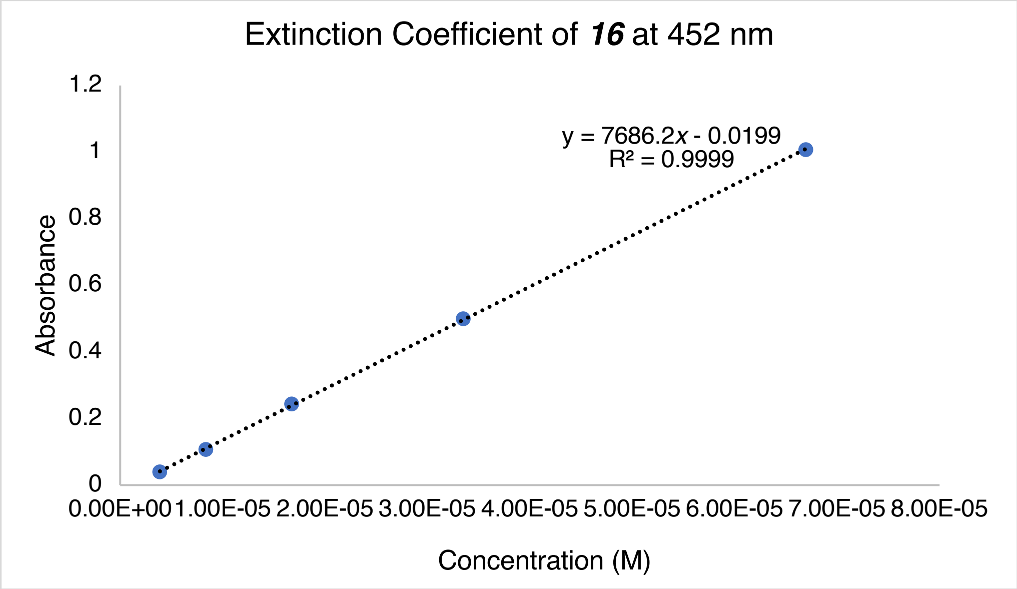

Supplementary Figure 6. Beer-Lambert plot of **16** at 452 nm. (ε = 7.69 x 10^3 M^{-1} cm^{-1}).

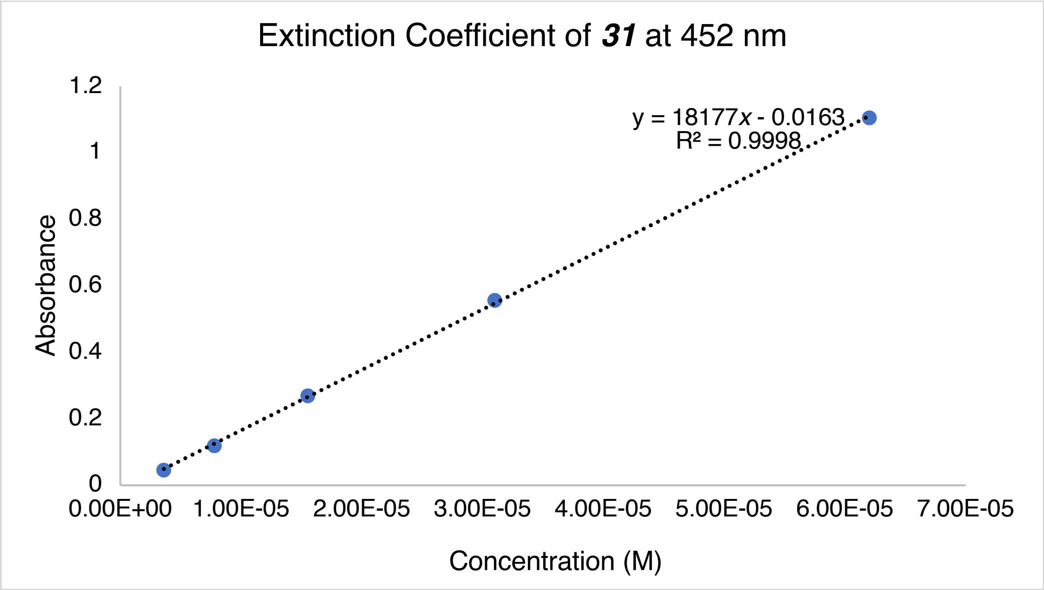

Supplementary Figure 7. Beer-Lambert plot of **31** at 452 nm. (ε = 18.2 x 10^4 M^{-1} cm^{-1}).

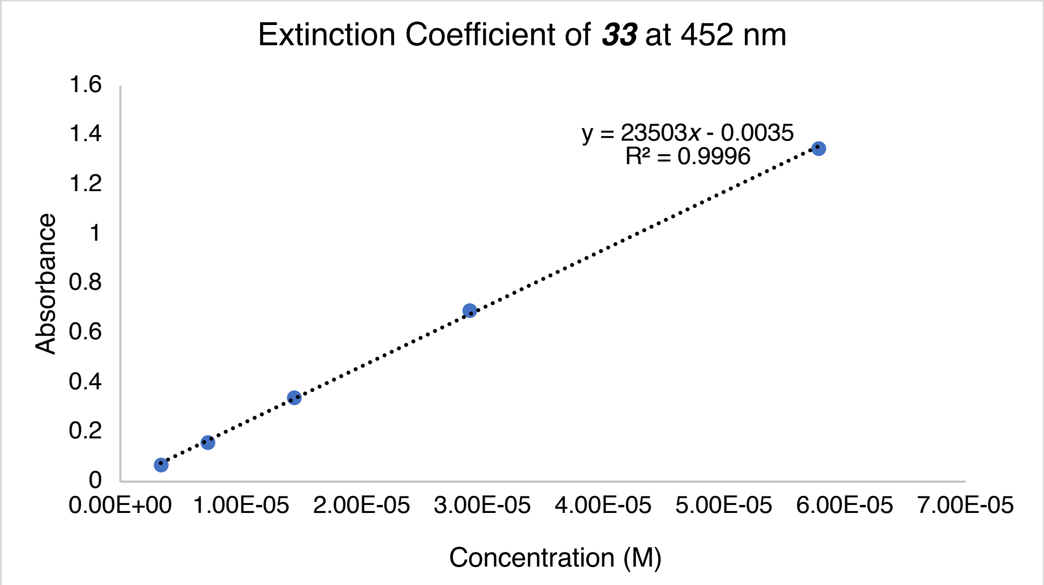

Supplementary Figure 8. Beer-Lambert plot of **33** at 452 nm. (ε = 23.5 x 10^4 M^{-1} cm^{-1}).

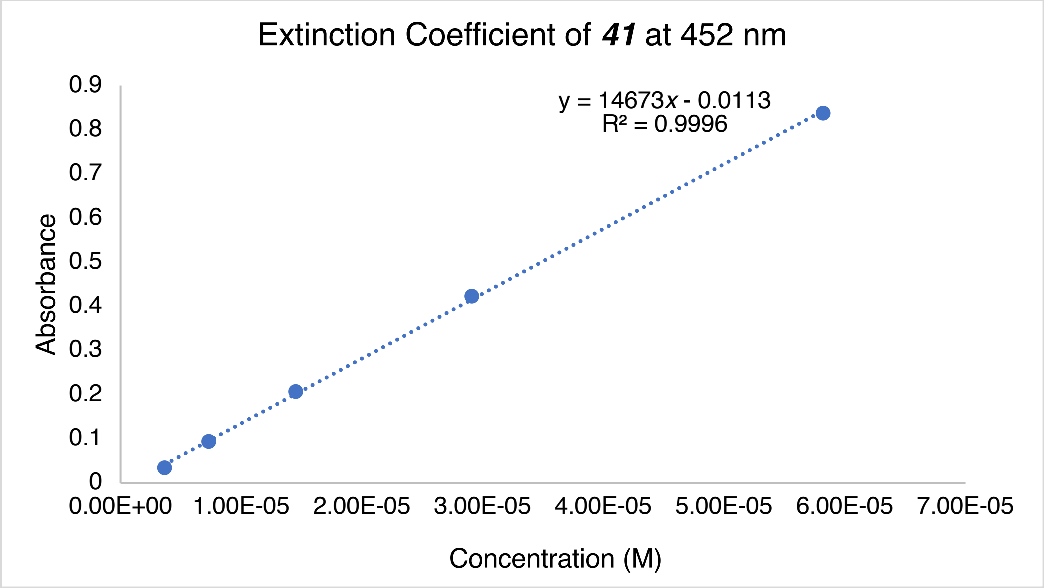

Supplementary Figure 9. Beer-Lambert plot of **41** at 452 nm. (ε = 14.7 x 10^4 M^{-1} cm^{-1}).

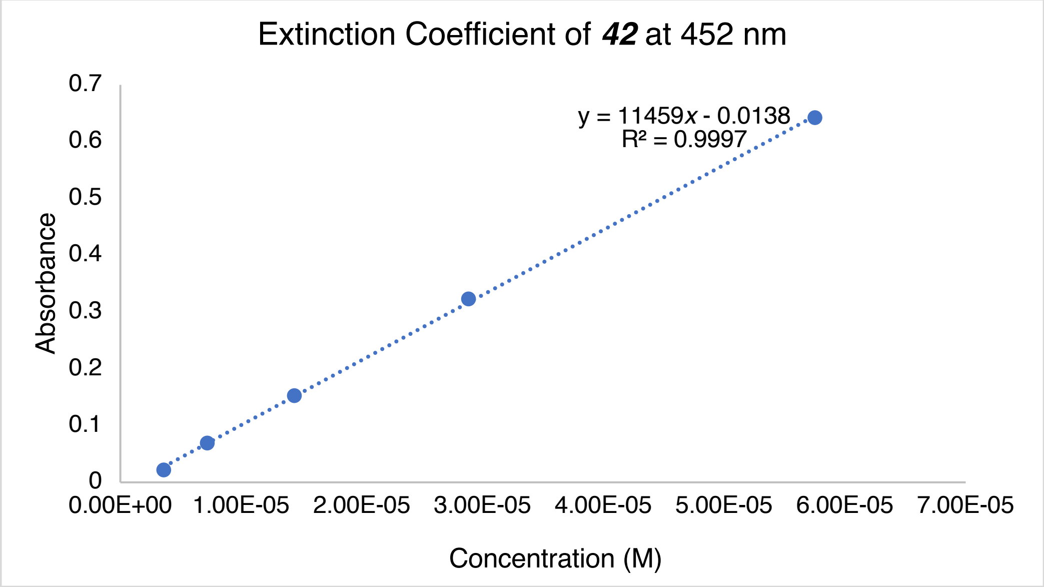

Supplementary Figure 10. Beer-Lambert plot of **42** at 452 nm. (ε = 11.5 x 10^3 M^{-1} cm^{-1}).

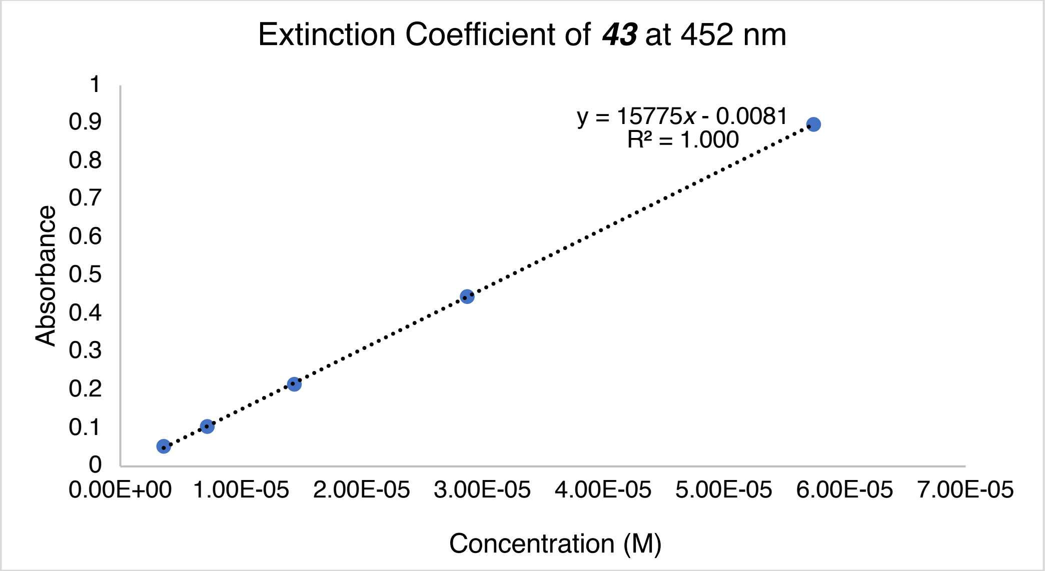

Supplementary Figure 11. Beer-Lambert plot of **43** at 452 nm. ($\varepsilon = 15.8 \times 10^4\ M^{-1}\ cm^{-1}$).

D. Quantum Yield Measurements

The phosphorescence quantum yield (Φ_P) of a molecule or material is defined as follows:

$$\Phi_P = \frac{PE}{PA}$$

Where PE and PA represent the number of photons emitted and absorbed, respectively. To determine the quantum yield, we used a relative method with $Ru(bpy)_3[PF_6]_2$ as a known standard in the same region of the electromagnetic spectrum.

To compare an unknown to a reference with a known quantum yield, the following relationship was used:

$$\Phi_{P,x} = \Phi_{P,r}\left(\frac{m_x}{m_r}\right)\left(\frac{n_x}{n_r}\right)$$

Where m represents the slope of the line (y = mx + b) obtained from graphing integrated phosphorescence intensity versus optical density across a series of samples, η is the refractive index of the solvent and the subscripts x and r represent values of the unknown and reference, respectively.

The $\Phi_{P,r}$ of $Ru(bpy)_3[PF_6]_2$ in degassed acetonitrile was taken to be 9.5%, as previously determined.[6]

To obtain a plot of integrated phosphorescence intensity versus absorbance for the reference and unknown, five solutions and a solvent blank were prepared with absorbance at 452 nm between

0.02 and 0.30 au. Absorbance and emission spectra (with an excitation wavelength of 450 nm) were acquired for all samples. $Ru(bpy)_3[PF_6]_2$ and the unknown Ru complexes were diluted in acetonitrile to concentrations with optical densities at 452 nm of less than 0.3 to minimize effects of reabsorption. The phosphorescence traces were integrated, and the raw integrals were corrected by subtracting the integral over an identical range from phosphorescence traces of the blank solvent. The integrated phosphorescence intensities were then plotted against the baseline corrected absorbance values at the relevant wavelength (450 nm), and the slope and error in slope were obtained ($R^2 > 0.98$ for all traces).

Computational Data

Calculations were carried out with the Gaussian 16 package. Geometry optimization was performed with B3LYP with LANL2DZ basis set for Ru atom and 6-31G(d) basis set for all other atoms (C, H, N); CPCM (MeCN) solvation model was utilized.[7] Computed structures are illustrated using CYLView.[8]

A. Complete Citation of Gaussian 16

M. J. Frisch, G. W. Trucks, H. B. Schlegel, G. E. Scuseria, M. A. Robb, J. R. Cheeseman, G. Scalmani, V. Barone, G. A. Petersson, H. Nakatsuji, X. Li, M. Caricato, A. V. Marenich, J. Bloino, B. G. Janesko, R. Gomperts, B. Mennucci, H. P. Hratchian, J. V. Ortiz, A. F. Izmaylov, J. L. Sonnenberg, D. Williams-Young, F. Ding, F. Lipparini, F. Egidi, J. Goings, B. Peng, A. Petrone, T. Henderson, D. Ranasinghe, V. G. Zakrzewski, J. Gao, N. Rega, G. Zheng, W. Liang, M. Hada, M. Ehara, K. Toyota, R. Fukuda, J. Hasegawa, M. Ishida, T. Nakajima, Y. Honda, O. Kitao, H. Nakai, T. Vreven, K. Throssell, J. A. Montgomery, Jr., J. E. Peralta, F. Ogliaro, M. J. Bearpark, J. J. Heyd, E. N. Brothers, K. N. Kudin, V. N. Staroverov, T. A. Keith, R. Kobayashi, J. Normand, K. Raghavachari, A. P. Rendell, J. C. Burant, S. S. Iyengar, J. Tomasi, M. Cossi, J. M. Millam, M. Klene, C. Adamo, R. Cammi, J. W. Ochterski, R. L. Martin, K. Morokuma, O. Farkas, J. B. Foresman, and D. J. Fox, Gaussian, Inc., Wallingford CT, **2016**.

B. Energy and Cartesian Coordinates for Optimized Structure

Diels–Alder Product **39**:

Ru	4.40809	-0.00012	-0.00007
C	6.22913	2.03055	-1.16572
C	6.57862	1.88488	1.13838
C	7.22284	3.00866	-1.27044
C	7.57559	2.85342	1.09803
H	6.28918	1.4164	2.07072
C	7.90368	3.42517	-0.13021
H	7.46637	3.44296	-2.23182
H	8.0771	3.1454	2.01373
H	8.67631	4.18324	-0.20307

C	3.79384	0.04896	-3.03046
C	5.45287	1.52931	-2.31722
C	3.91518	0.47135	-4.34978
H	3.08641	-0.72173	-2.75111
C	5.62078	1.99568	-3.62464
C	4.8463	1.46386	-4.65154
H	3.28956	0.02634	-5.11515
H	6.34779	2.76764	-3.84278
H	4.97009	1.82056	-5.66857
N	5.91592	1.4774	0.03975
N	4.54011	0.55823	-2.03255
C	2.82646	2.63885	0.39094
C	1.54248	0.71709	0.10359
C	1.67673	3.42417	0.49829
H	3.81553	3.07389	0.46351
C	0.33237	1.43315	0.2052
C	0.432	2.82417	0.40613
H	1.77165	4.49286	0.65347
H	-0.45666	3.43751	0.48978
C	1.54243	-0.71674	-0.10598
C	2.82622	-2.63856	-0.39372
C	0.33226	-1.43263	-0.20813
C	1.67643	-3.42364	-0.50206
H	3.81525	-3.07373	-0.46595
C	0.43176	-2.82352	-0.40997
H	1.77126	-4.49225	-0.65788
H	-0.45695	-3.43667	-0.49444
N	2.76551	1.31566	0.19906
N	2.76539	-1.31546	-0.20122
C	6.57918	-1.8862	-1.13547
C	6.22731	-2.03073	1.16837
C	7.57612	-2.85472	-1.09362
H	6.29059	-1.41823	-2.06831
C	7.22103	-3.00865	1.2746
C	7.90306	-3.4257	0.13528
H	8.07852	-3.14723	-2.00866
H	7.46367	-3.44245	2.23641
H	8.67569	-4.18365	0.20936
C	3.78911	-0.04915	3.02935
C	5.44945	-1.52931	2.31874
C	3.90846	-0.4715	4.34886
H	3.0819	0.72136	2.74891
C	5.61538	-1.99563	3.62644
C	4.83922	-1.46387	4.65211
H	3.28151	-0.02659	5.1132
H	6.34199	-2.76762	3.8458

H	4.96142	-1.82058	5.66933
N	4.53702	-0.55834	2.03263
N	5.91542	-1.47812	-0.0377
C	-0.94068	-0.7112	-0.10227
C	-2.15871	-1.37413	-0.19101
C	-0.94063	0.71175	0.09983
C	-3.40002	-0.71029	-0.0907
H	-2.17855	-2.44455	-0.34277
C	-2.15863	1.37467	0.18934
C	-3.40002	0.71086	0.08954
H	-2.17834	2.44503	0.34148
C	-4.64711	-1.41775	-0.17557
C	-4.64718	1.41819	0.17521
C	-5.84149	0.71582	0.08873
C	-5.84144	-0.71559	-0.0883
C	-4.63266	2.90284	0.38001
C	-4.86062	3.45042	1.6518
C	-4.36913	3.77147	-0.69099
C	-4.8351	4.83305	1.84646
H	-5.05974	2.78886	2.49018
C	-4.34524	5.15448	-0.49708
H	-4.18771	3.36037	-1.68052
C	-4.57877	5.68918	0.77241
H	-5.01456	5.24032	2.83779
H	-4.14515	5.81256	-1.3383
H	-4.56001	6.76491	0.9238
C	-7.14919	1.4443	0.18504
C	-7.91079	1.39474	1.36243
C	-7.63273	2.1922	-0.89888
C	-9.12462	2.07862	1.45487
H	-7.54854	0.82046	2.21048
C	-8.84906	2.87265	-0.80915
H	-7.05386	2.23849	-1.81699
C	-9.59862	2.81872	0.36851
H	-9.69914	2.03299	2.37618
H	-9.20965	3.44464	-1.65989
H	-10.54421	3.34923	0.43948
C	-7.14903	-1.44432	-0.18437
C	-7.63259	-2.19184	0.89979
C	-7.9104	-1.39549	-1.36194
C	-8.84875	-2.87261	0.81015
H	-7.05387	-2.23758	1.81802
C	-9.12407	-2.07969	-1.45429
H	-7.54813	-0.82149	-2.21017
C	-9.59811	-2.81939	-0.36768
H	-9.20936	-3.4443	1.66108

H	-9.69843	-2.03462	-2.37573
H	-10.54355	-3.35015	-0.43858
C	-4.6325	-2.9024	-0.38056
C	-4.85892	-3.44969	-1.65275
C	-4.37033	-3.77125	0.69058
C	-4.83324	-4.83229	-1.84767
H	-5.057	-2.78793	-2.49123
C	-4.34628	-5.15422	0.49641
H	-4.19003	-3.36036	1.6804
C	-4.57825	-5.68864	-0.77348
H	-5.01154	-5.23934	-2.83929
H	-4.14723	-5.81249	1.33773
H	-4.55935	-6.76434	-0.92507

^{1}H NMR Spectra

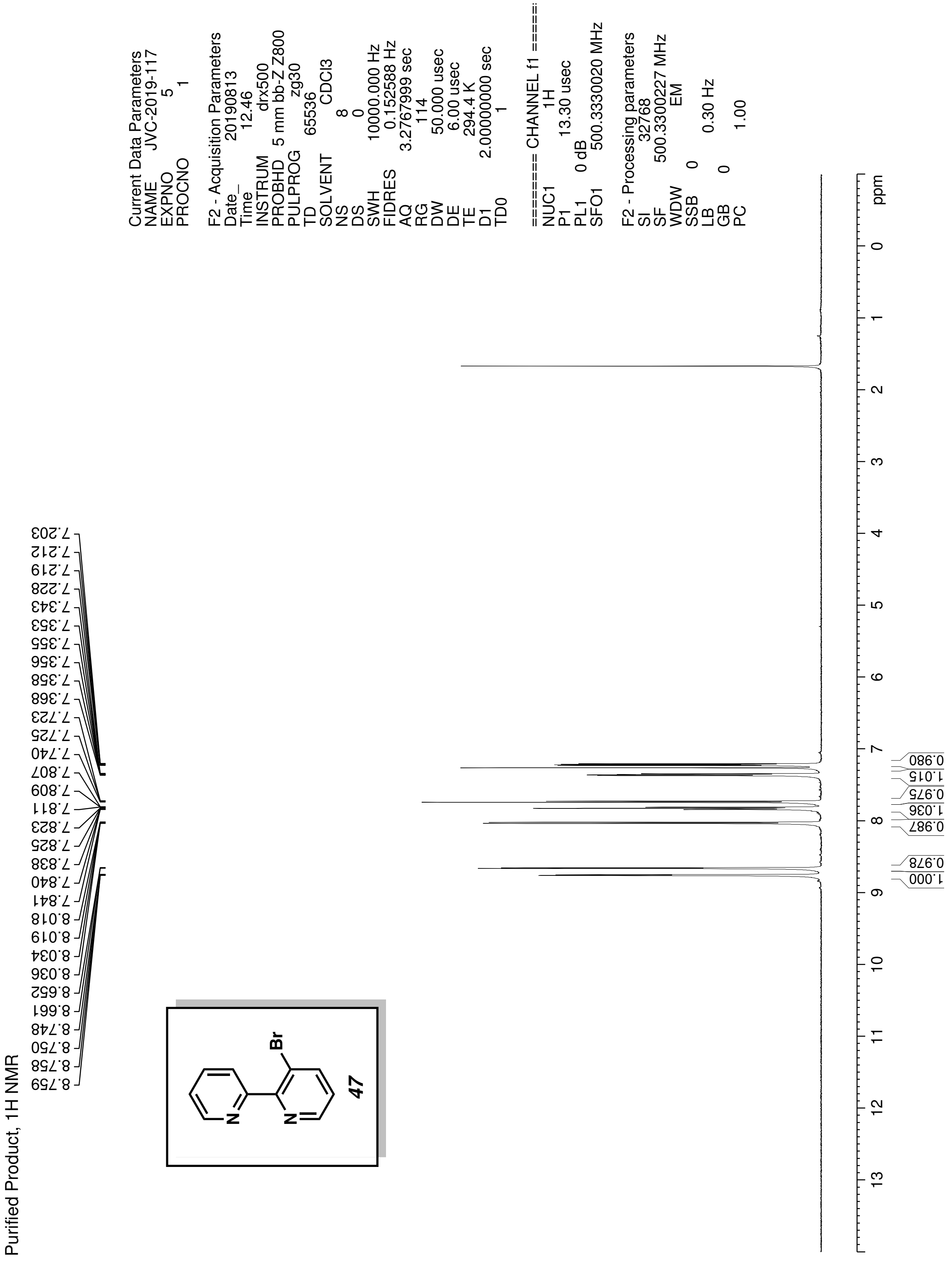

Supplementary Figure 12. ^{1}H NMR (500 MHz, $CDCl_3$) of 47.

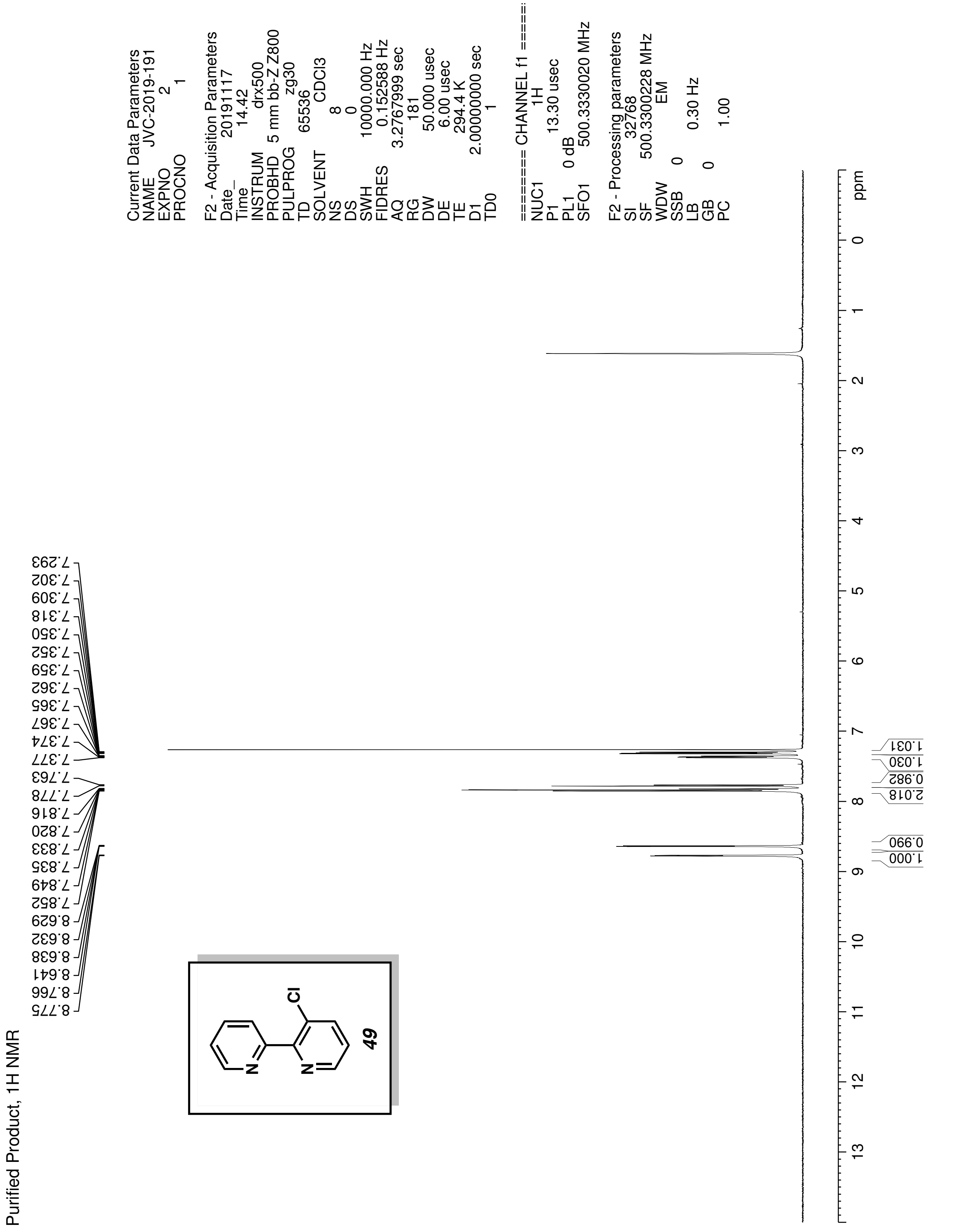

Supplementary Figure 13. ^{1}H NMR (500 MHz, $CDCl_3$) of 49.

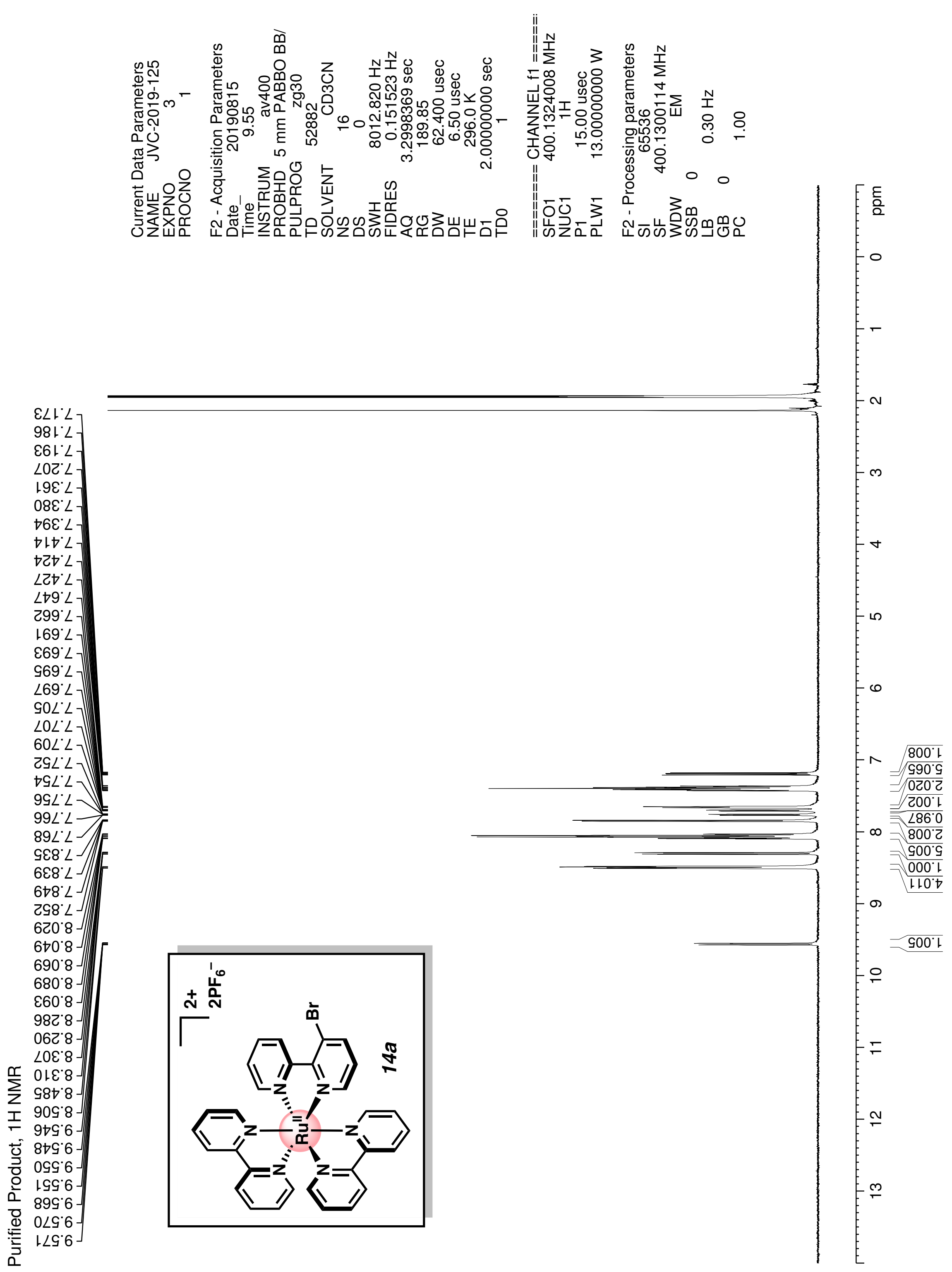

Supplementary Figure 14. ^{1}H NMR (400 MHz, CD_3CN) of 14a.

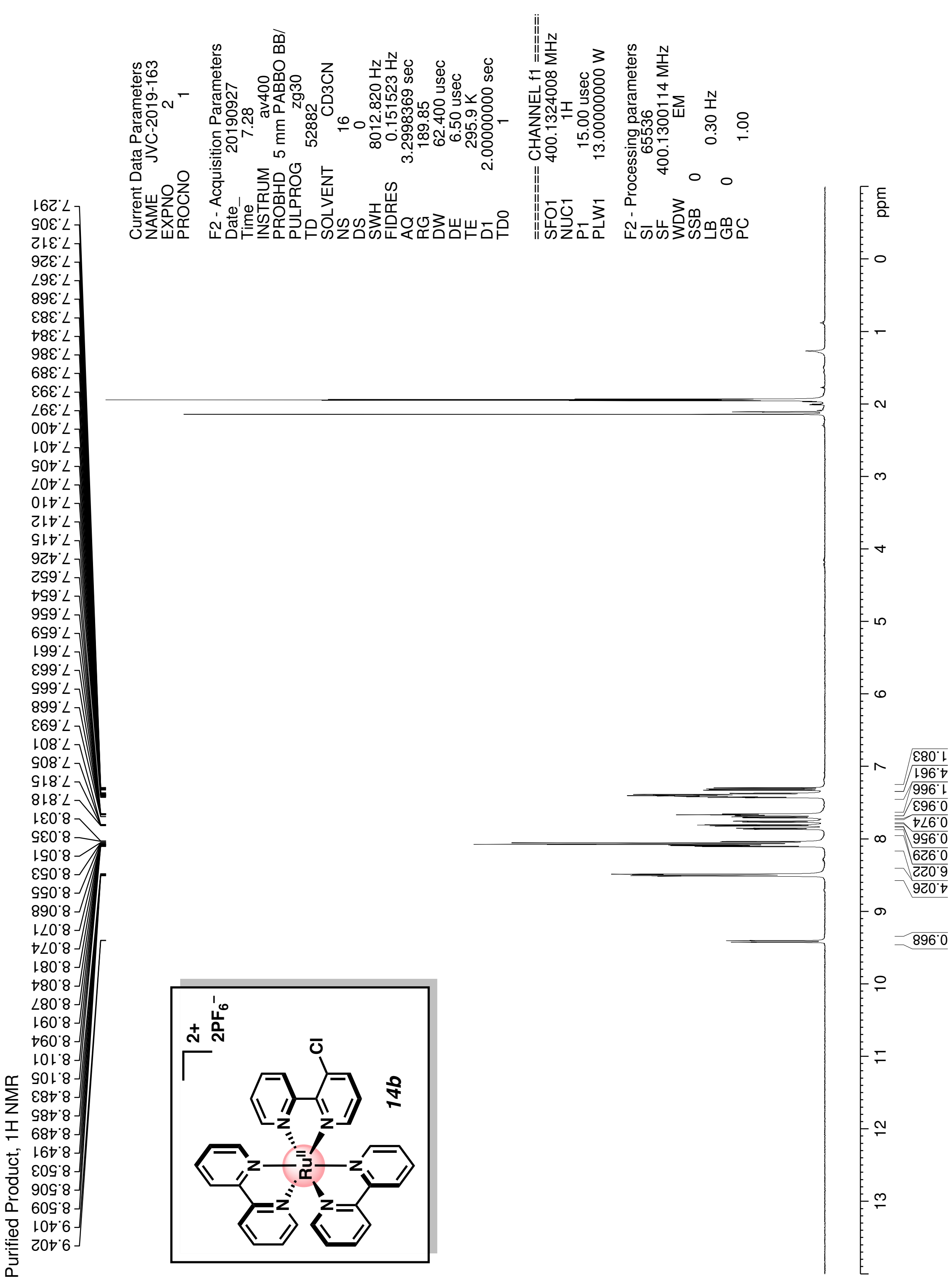

Supplementary Figure 15. ^{1}H NMR (400 MHz, CD_3CN) of 14b.

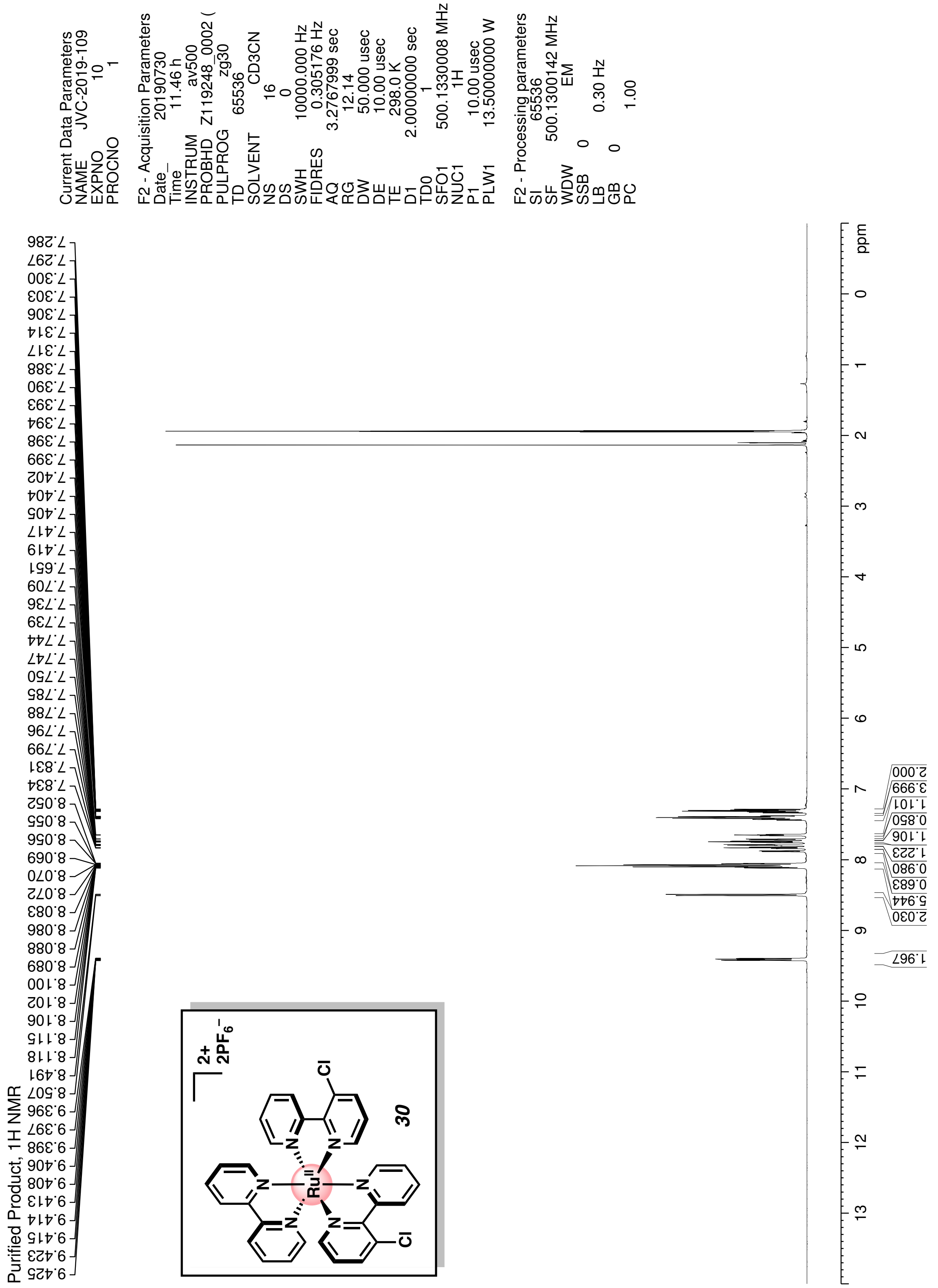

Supplementary Figure 16. ^{1}H NMR (500 MHz, CD_3CN) of 30.

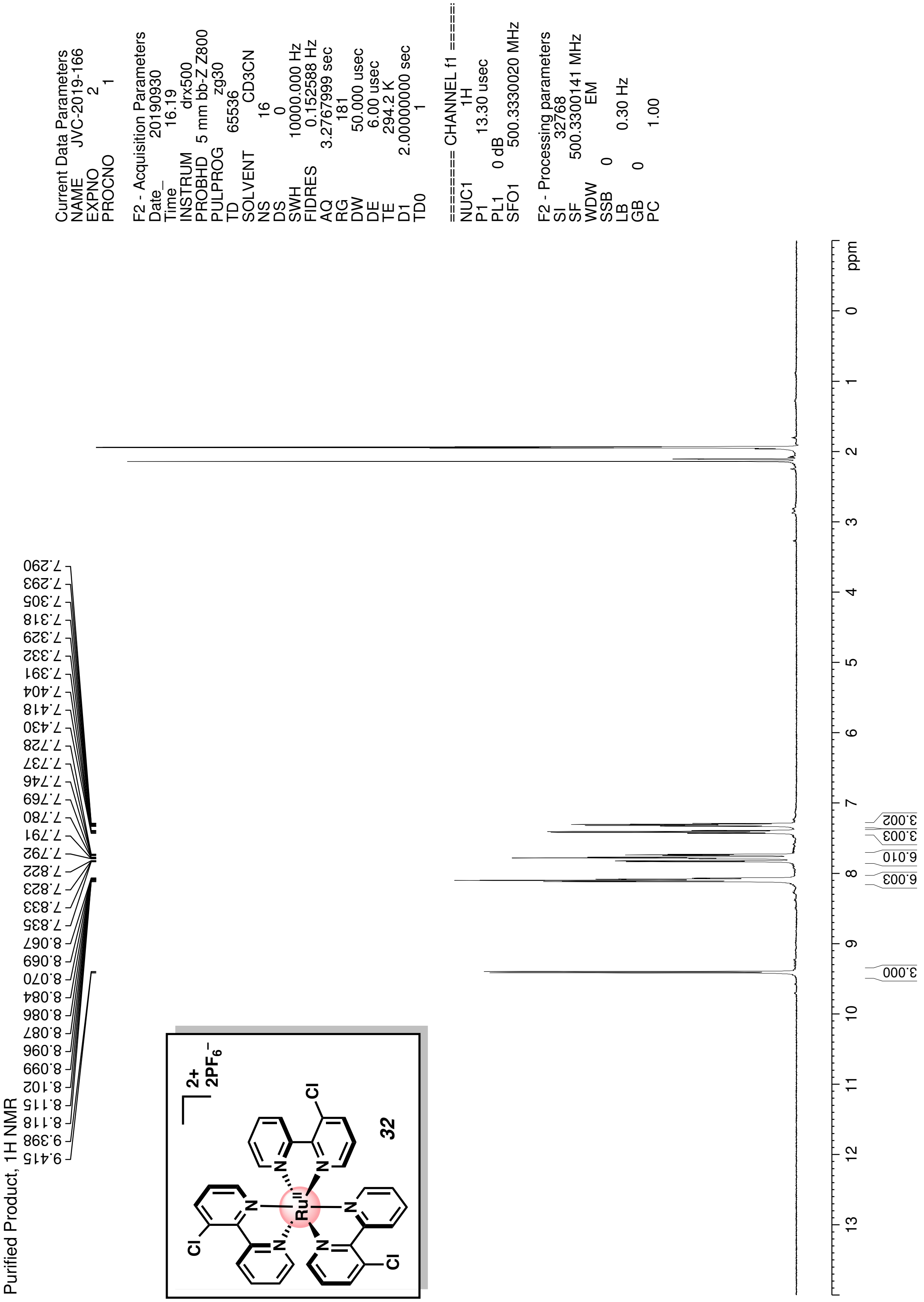

Supplementary Figure 17. ¹H NMR (500 MHz, CD_3CN) of 32.

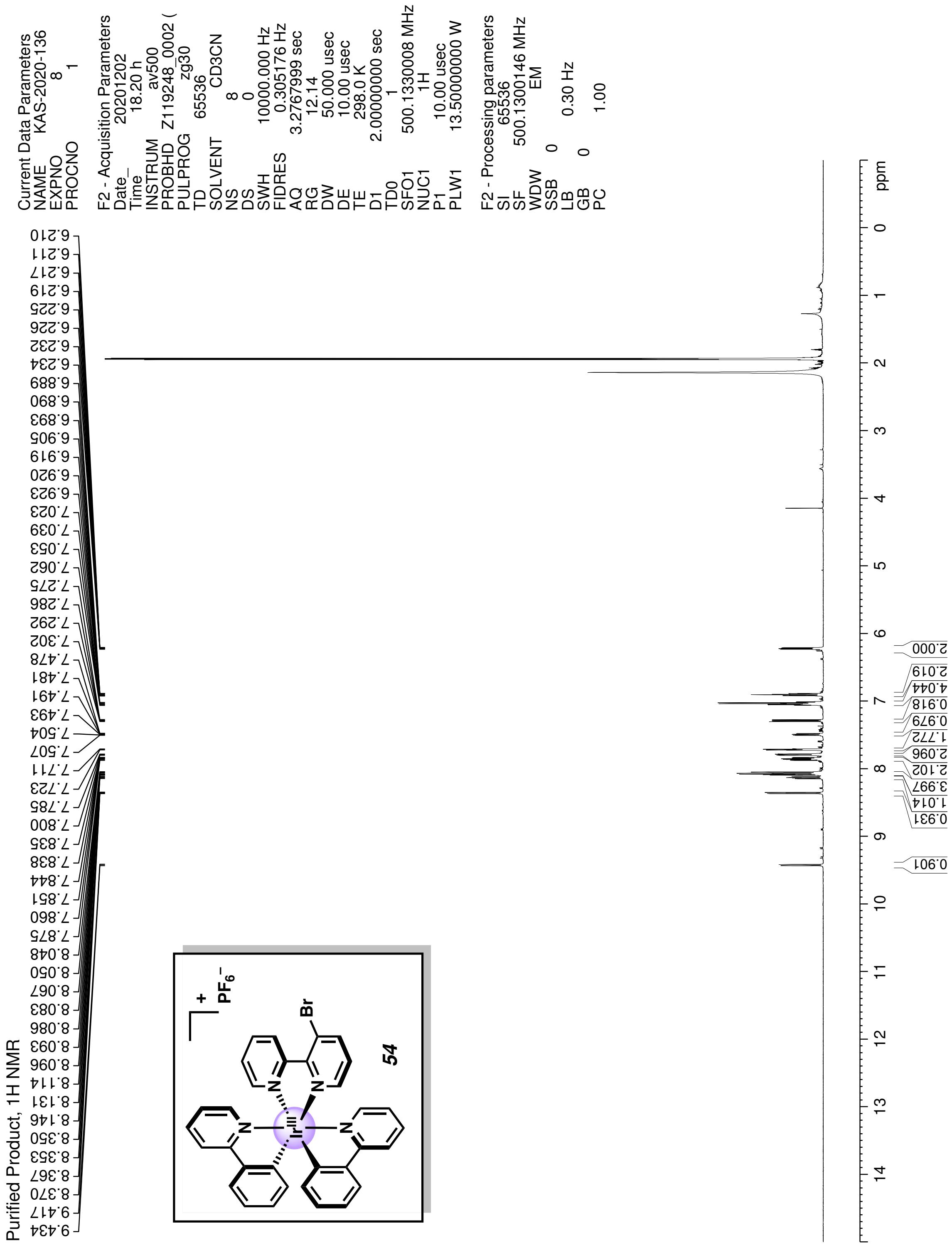

Supplementary Figure 18. ^{1}H NMR (500 MHz, CD_3CN) of 54.

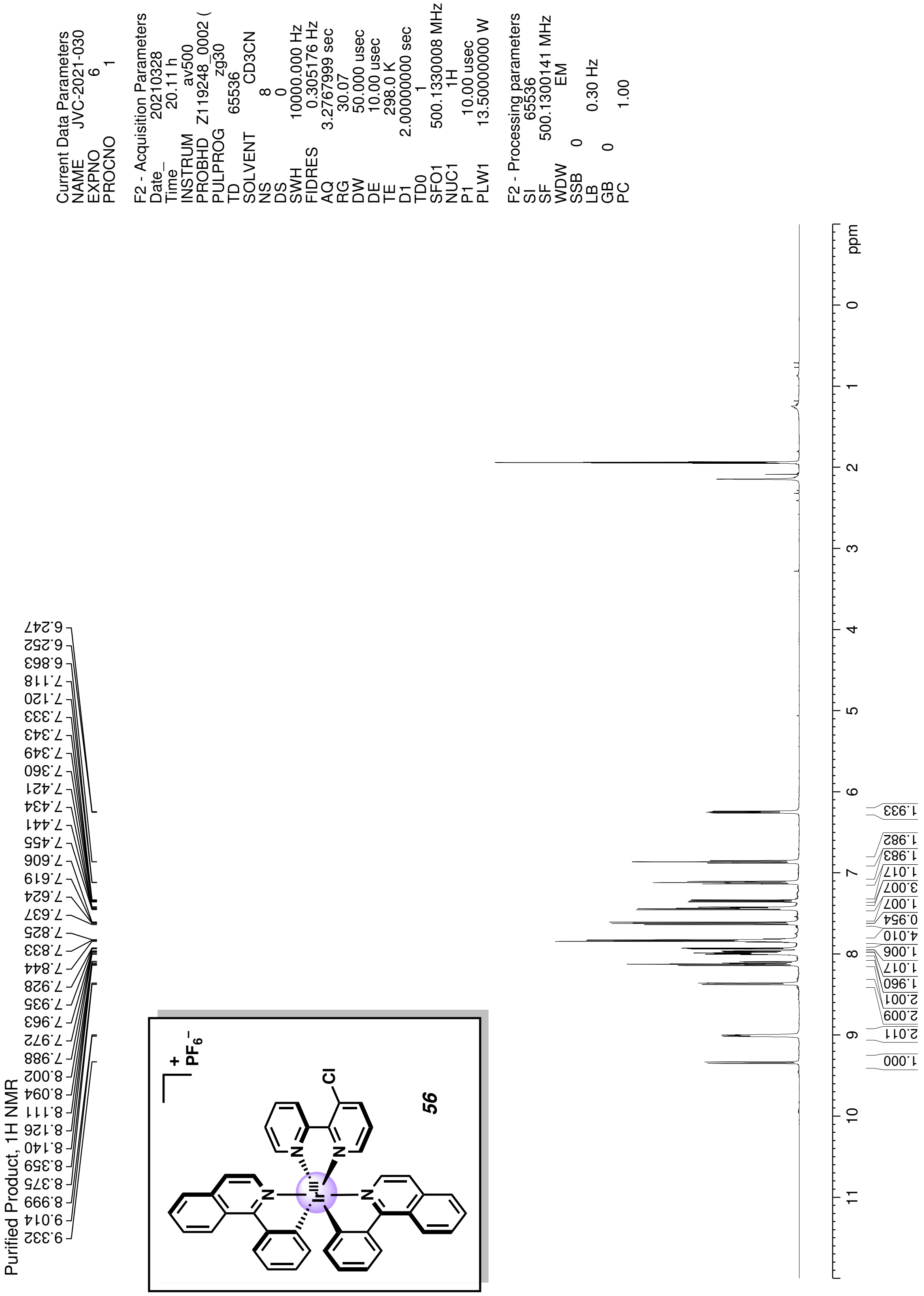

Supplementary Figure 19. ^{1}H NMR (500 MHz, CD_3CN) of 56.

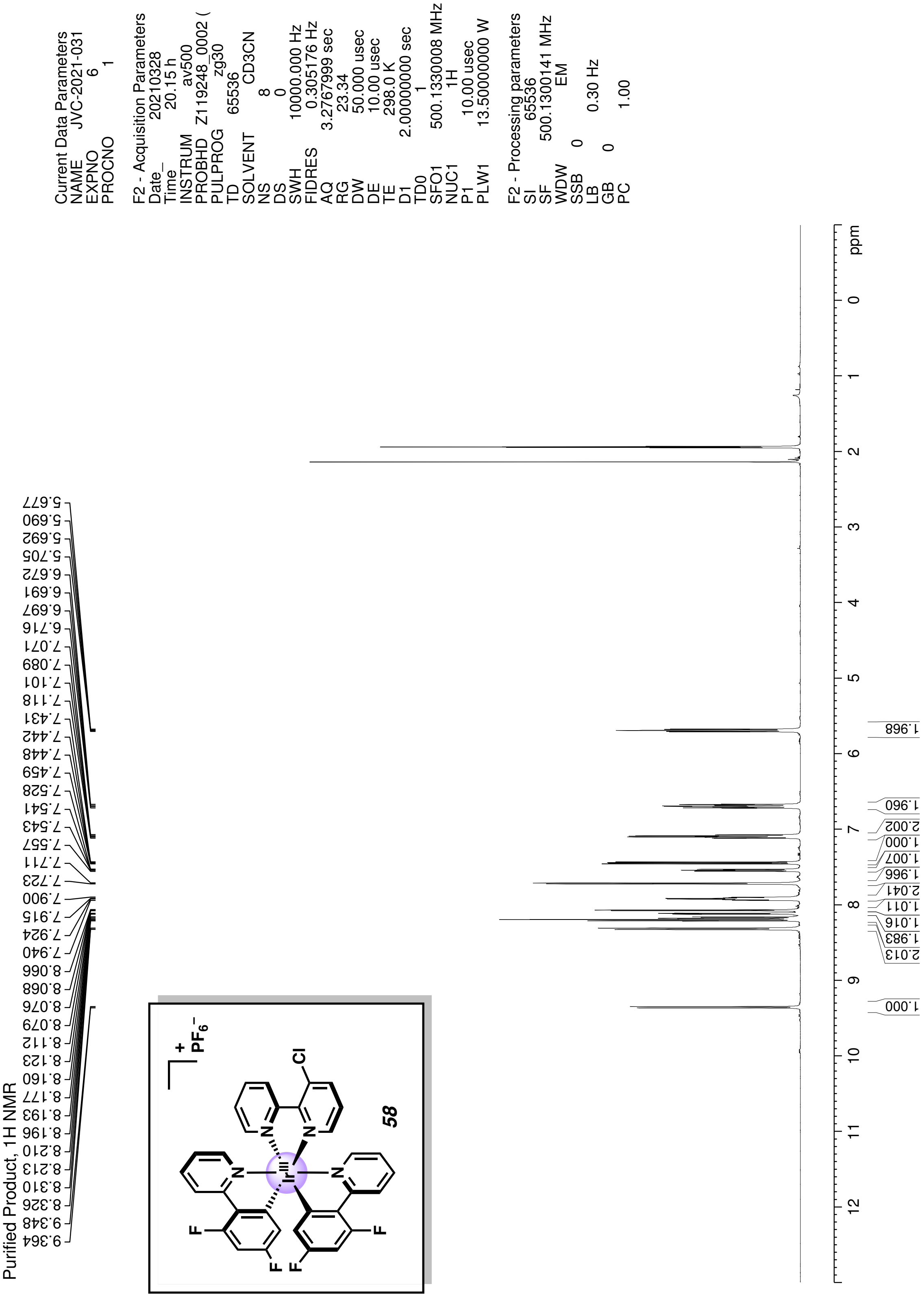

Supplementary Figure 20. ^{1}H NMR (500 MHz, CD_3CN) of 58.

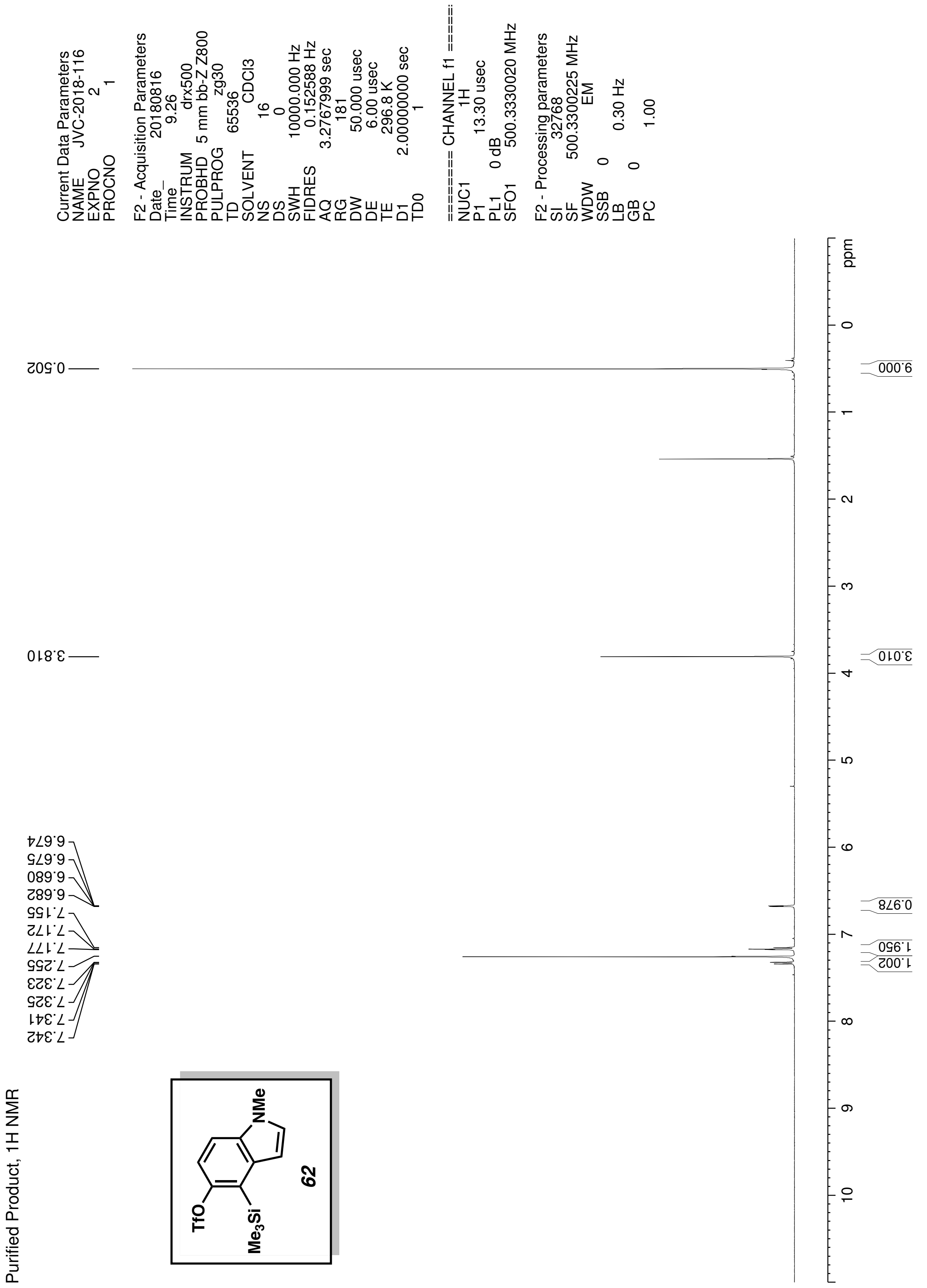

Supplementary Figure 21. ^{1}H NMR (500 MHz, CDCl$_3$) of 62.

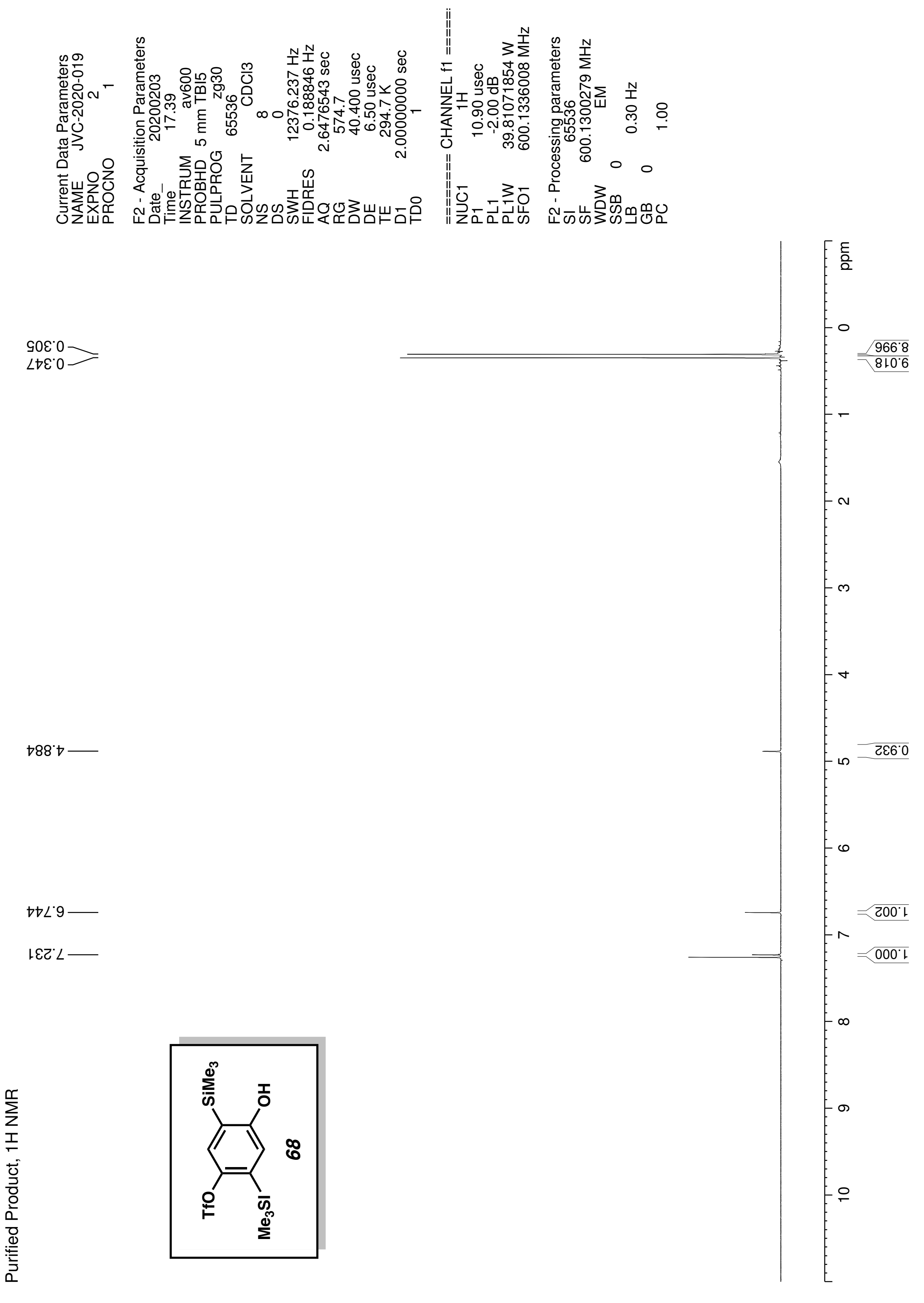

Supplementary Figure 22. ^{1}H NMR (600 MHz, $CDCl_3$) of 68.

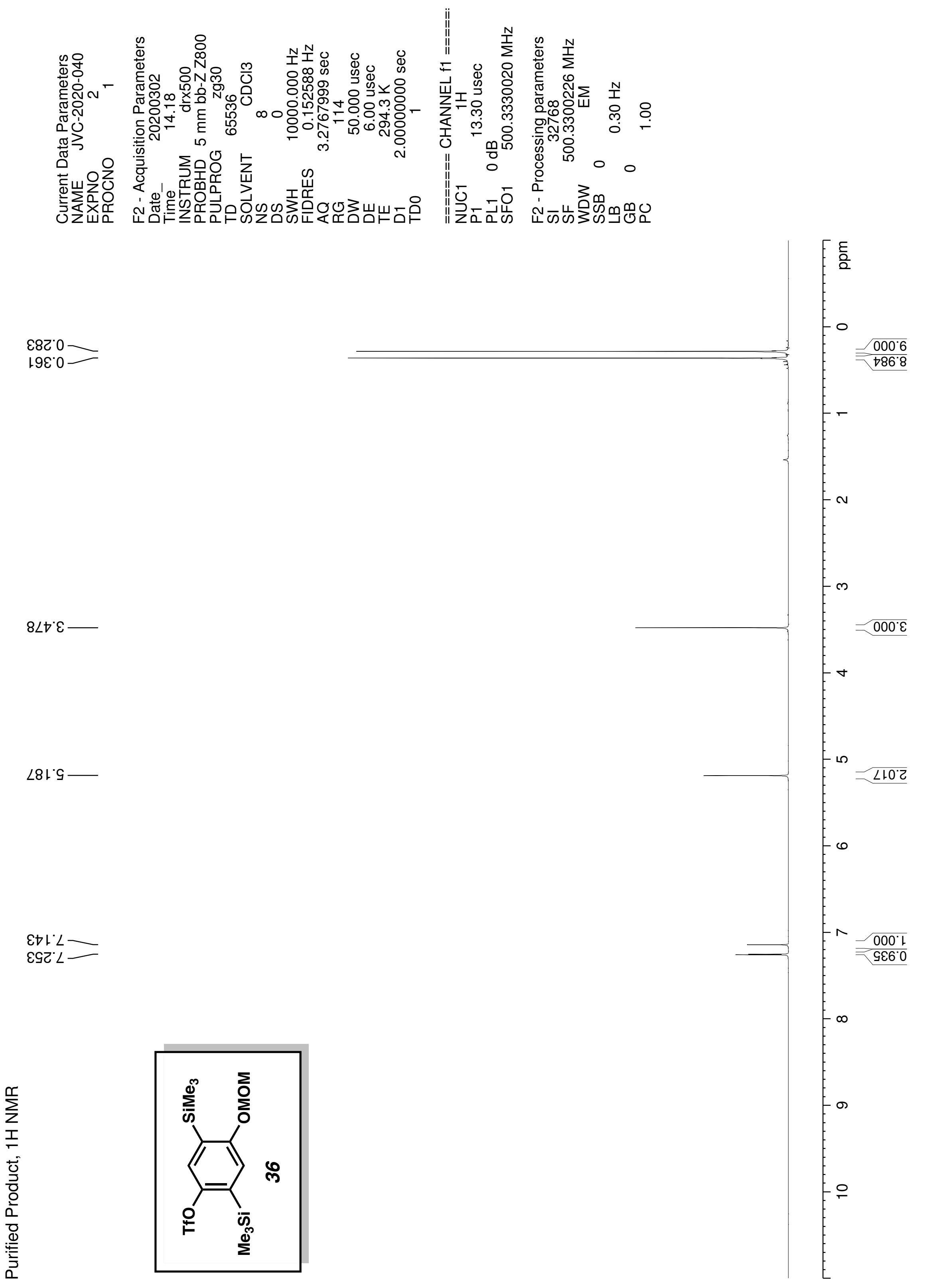

Supplementary Figure 23. ^{1}H NMR (500 MHz, CDCl$_3$) of 36.

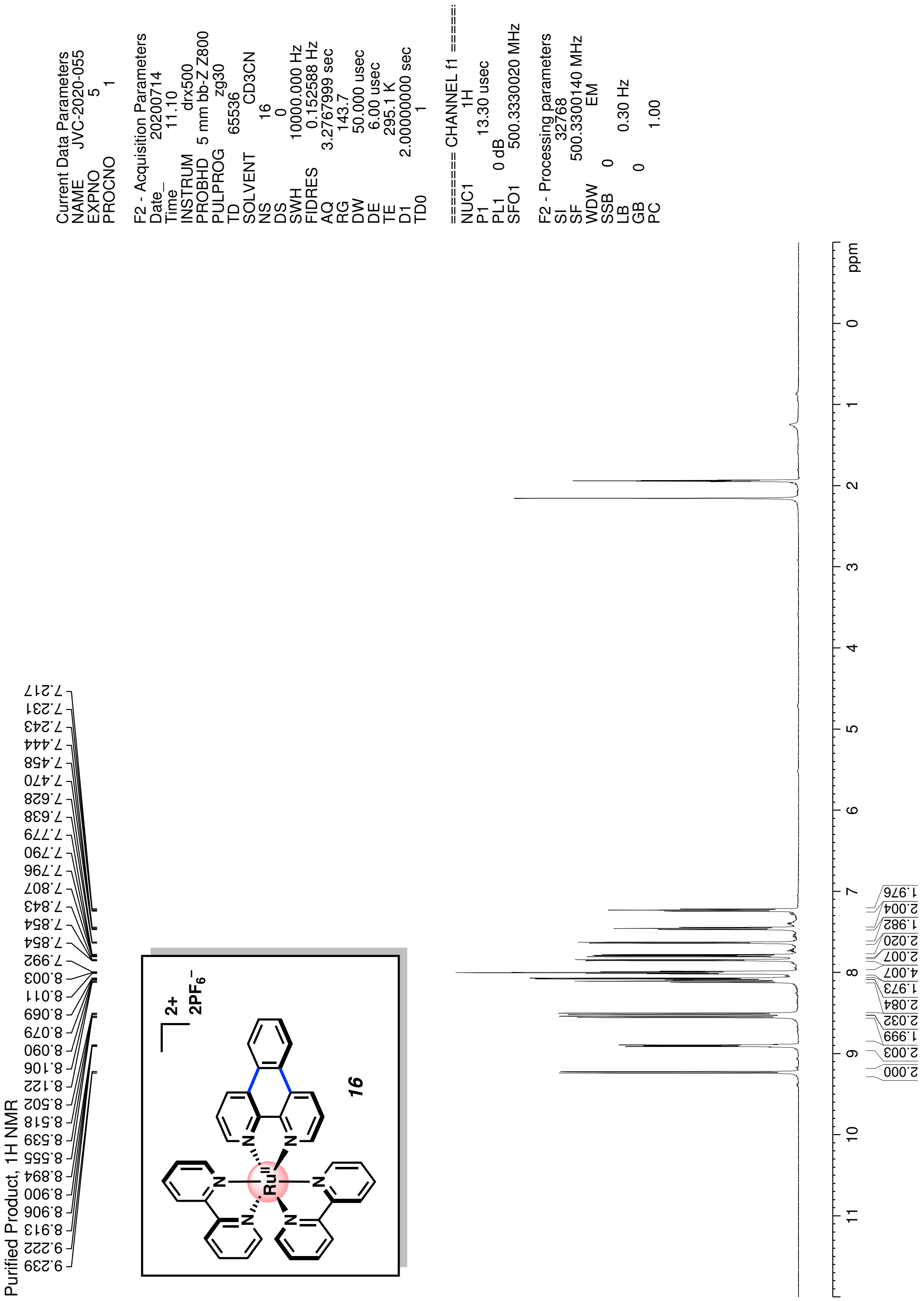

Supplementary Figure 24. ^{1}H NMR (500 MHz, CD_3CN) of 16.

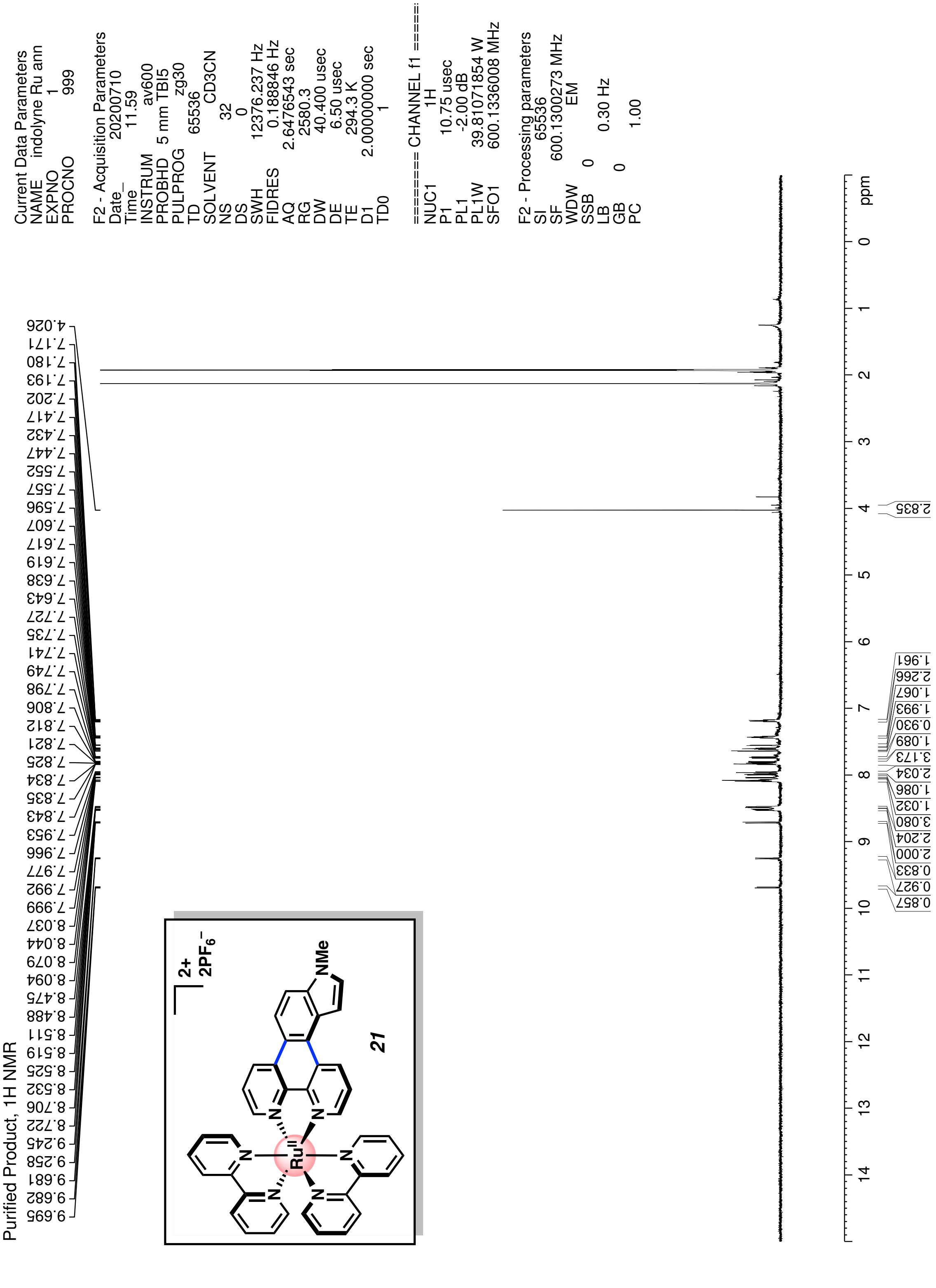

Supplementary Figure 25. ^{1}**H NMR (600 MHz, CD$_3$CN) of 21.**

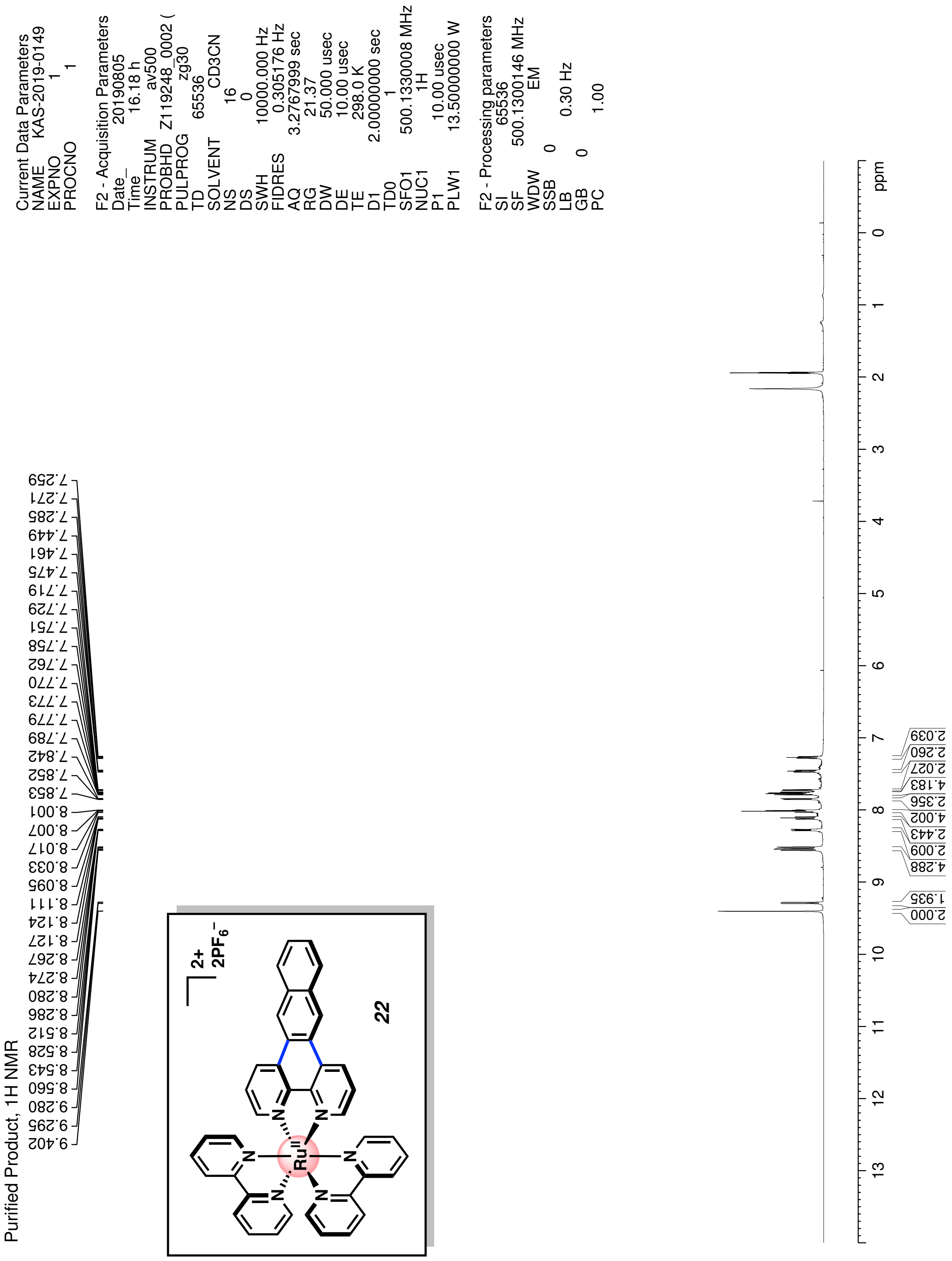

Supplementary Figure 26. ^{1}H NMR (500 MHz, CD_3CN) of 22.

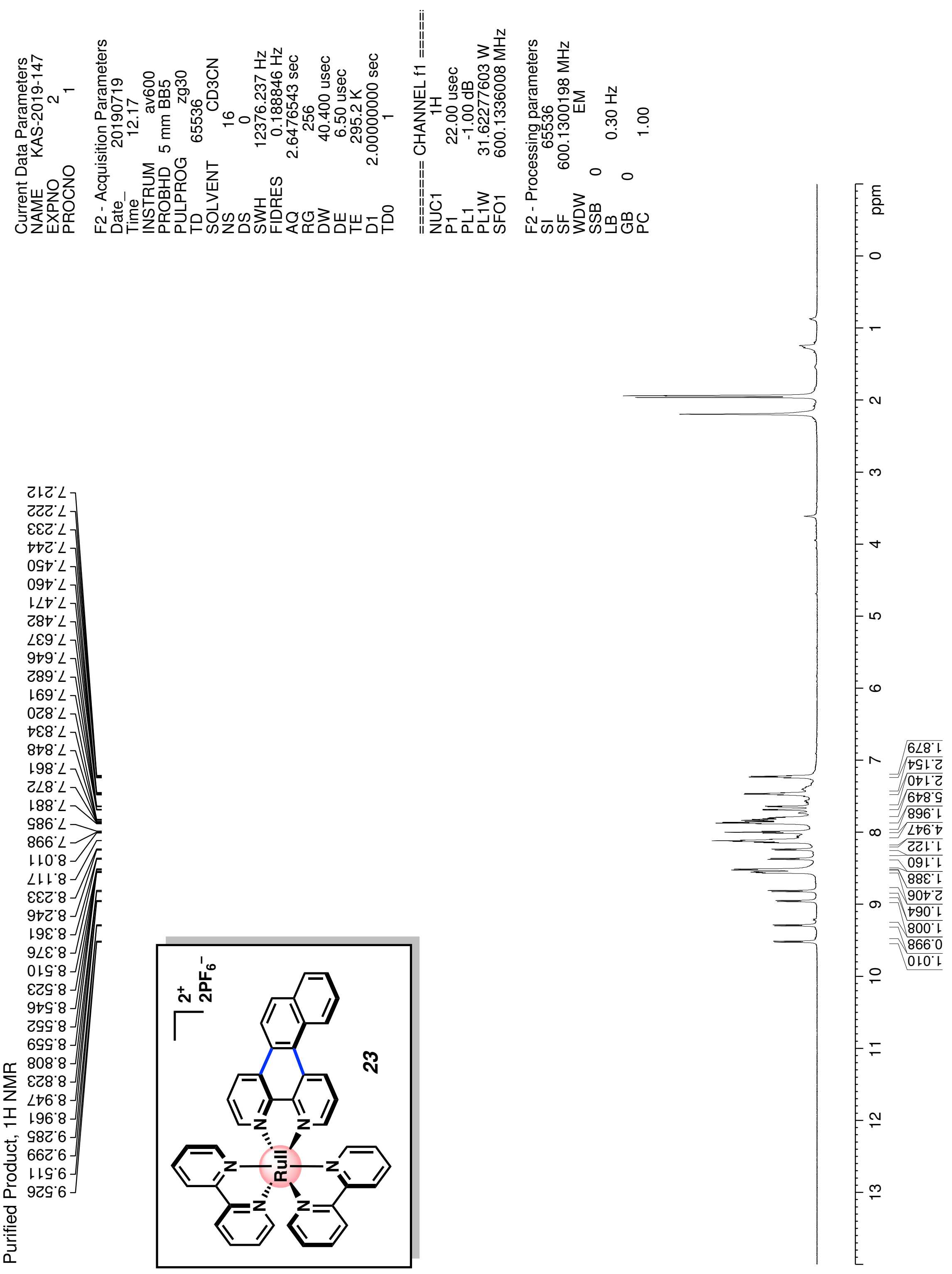

Supplementary Figure 27. ^{1}H NMR (600 MHz, CD_3CN) of 23.

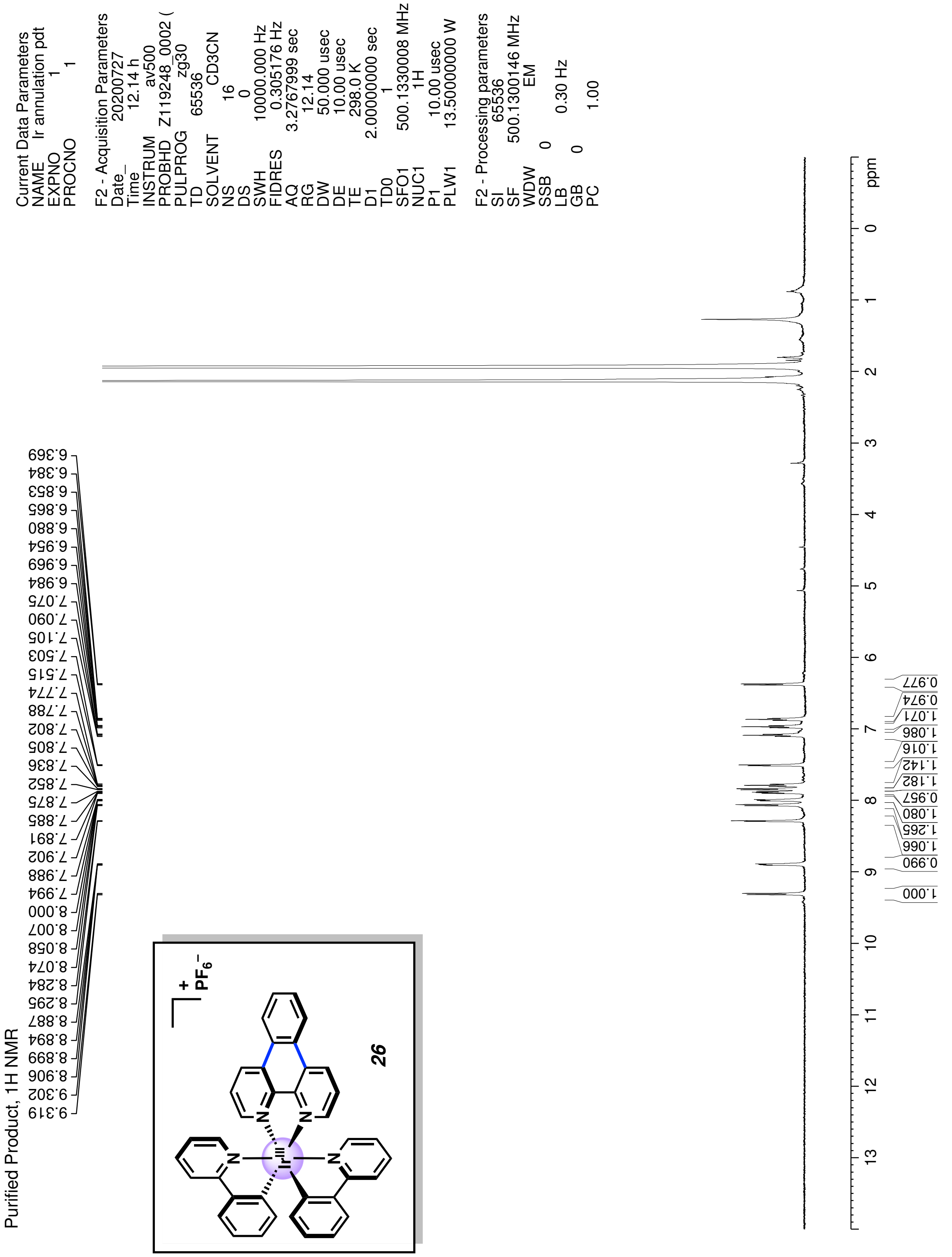

Supplementary Figure 28. ^{1}H NMR (500 MHz, CD_3CN) of 26.

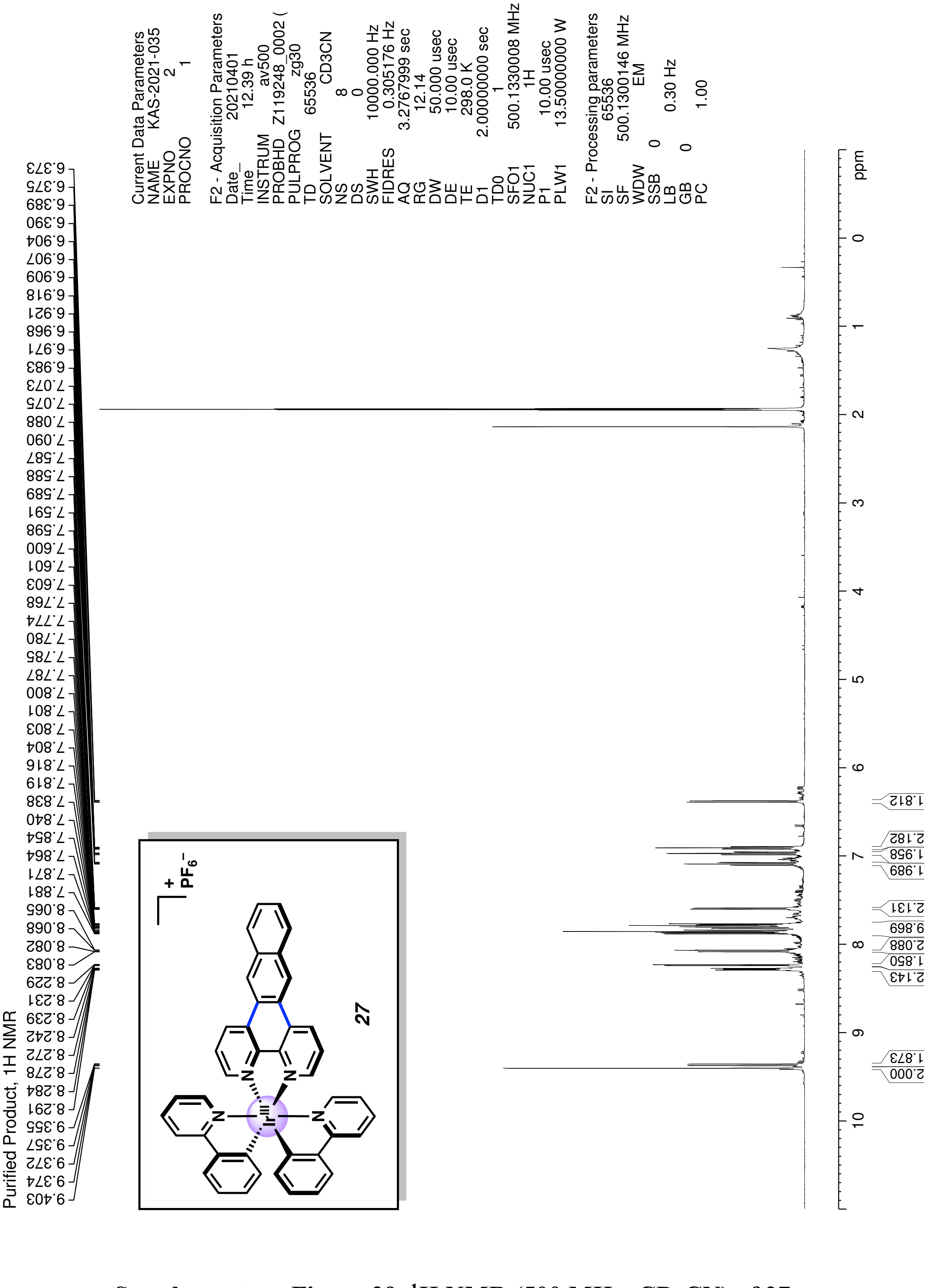

Supplementary Figure 29. ^{1}H NMR (500 MHz, CD_3CN) of 27.

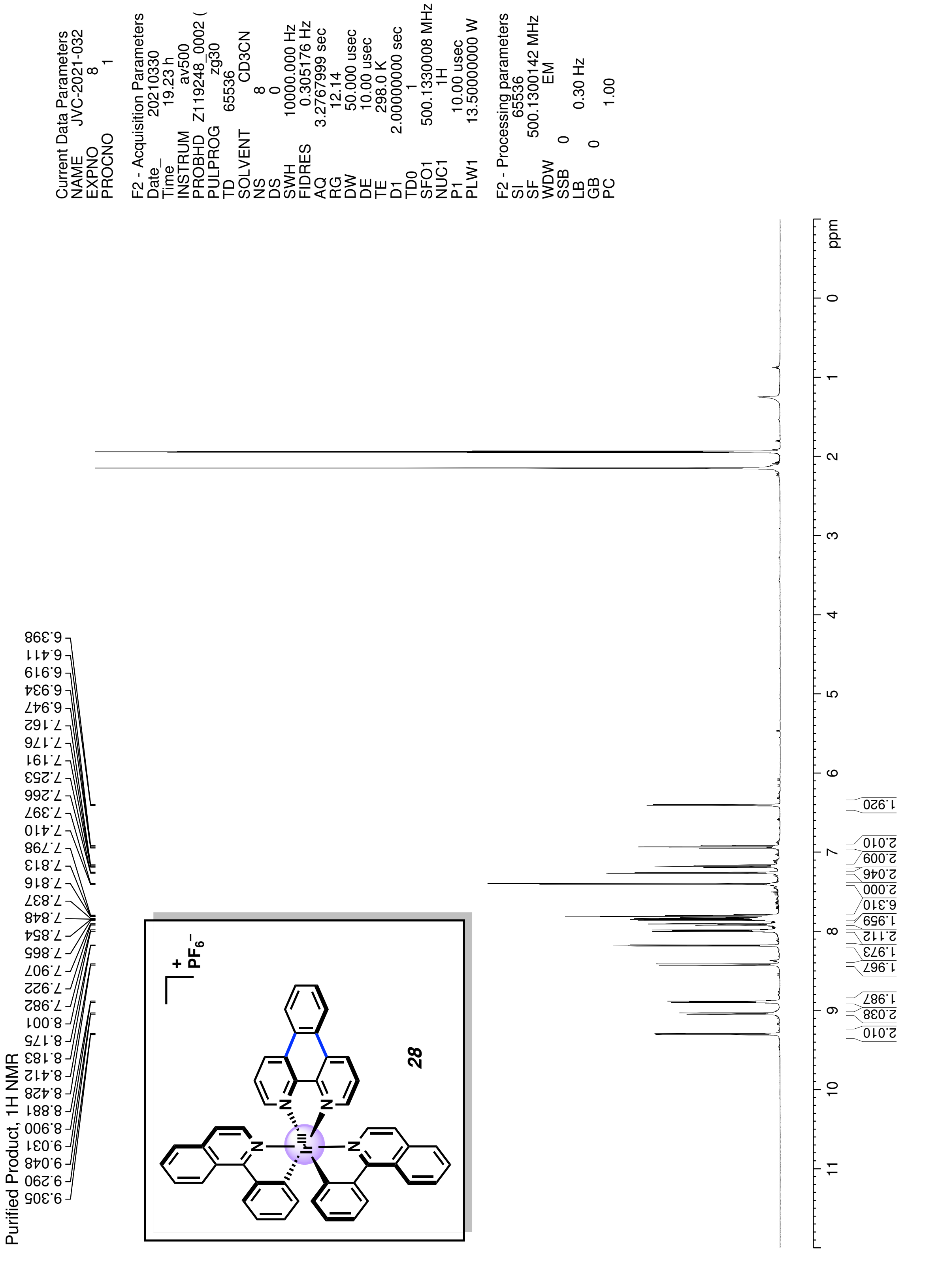

Supplementary Figure 30. ^{1}H NMR (500 MHz, CD_3CN) of 28.

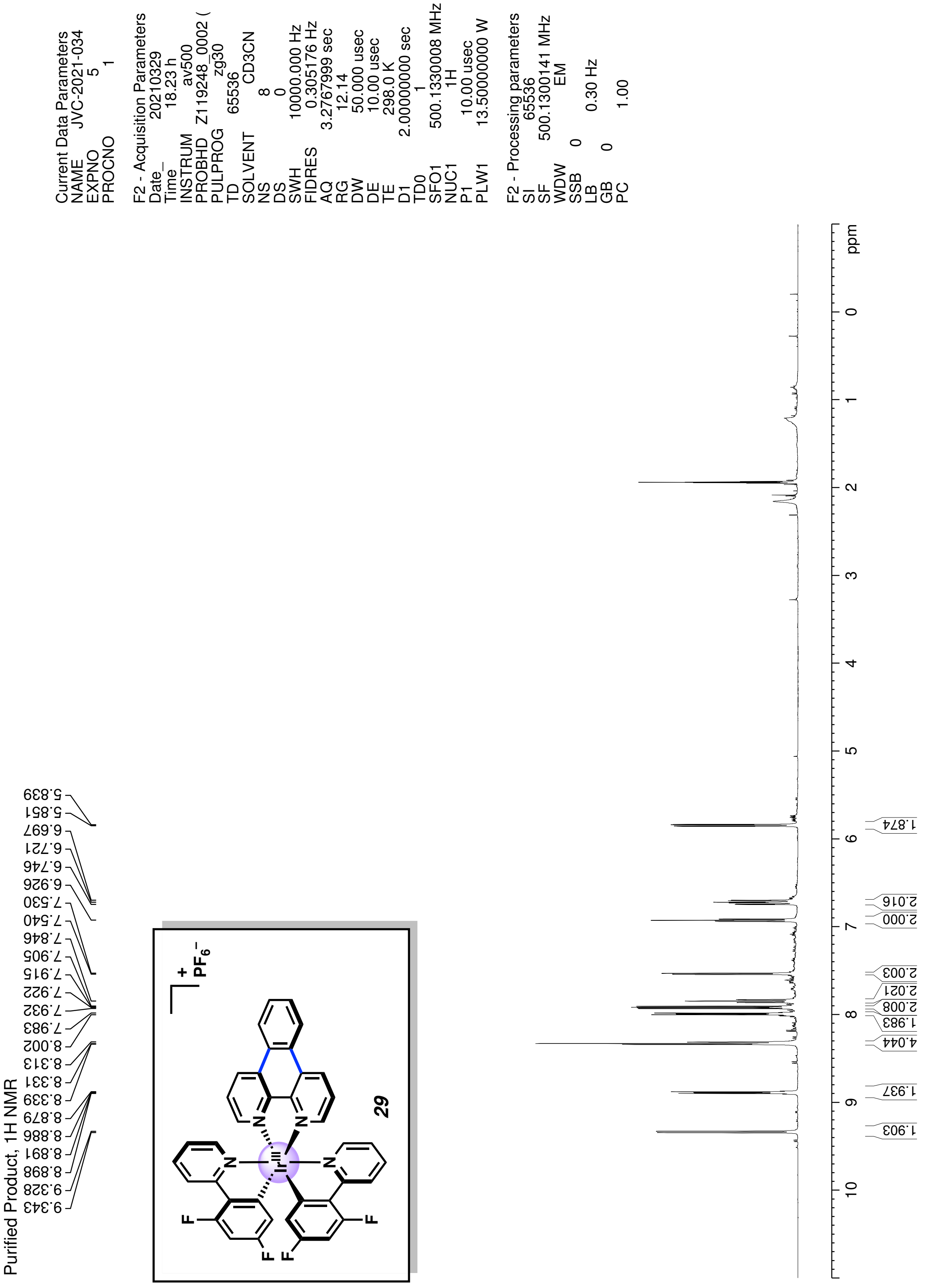

Supplementary Figure 31. ^{1}H NMR (500 MHz, CD_3CN) of 29.

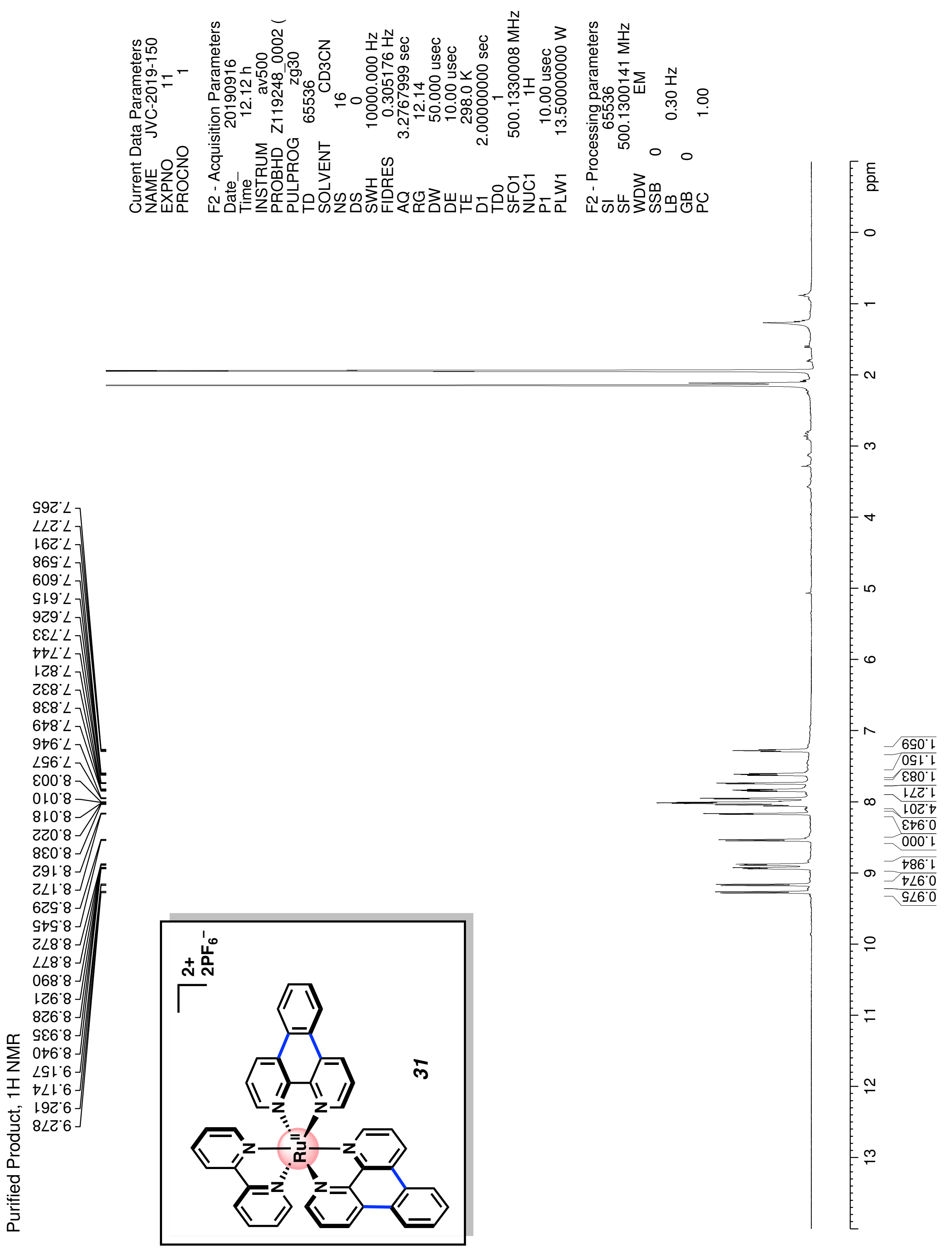

Supplementary Figure 32. ^{1}H NMR (500 MHz, CD_3CN) of 31.

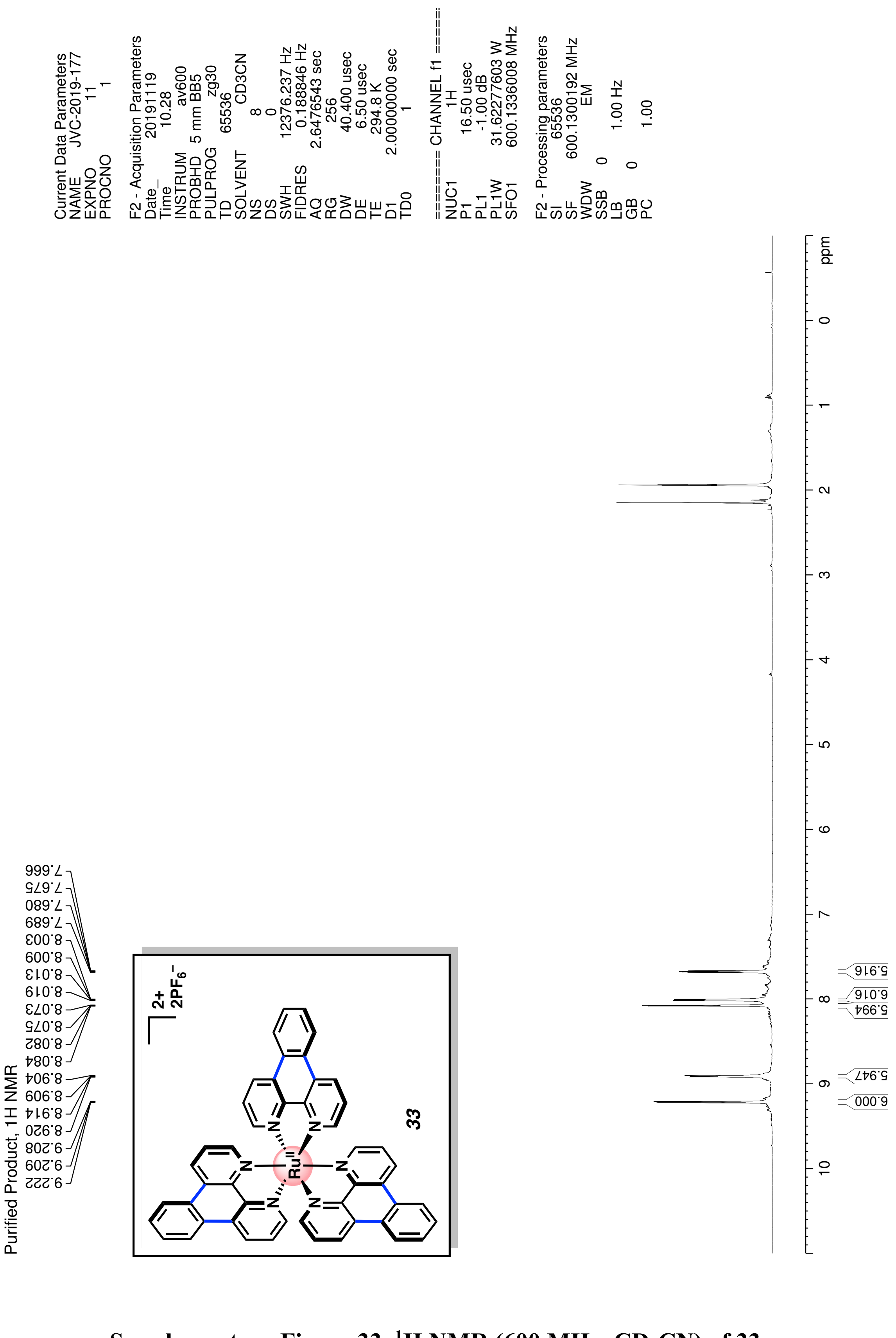

Supplementary Figure 33. ^{1}H NMR (600 MHz, CD_3CN) of 33.

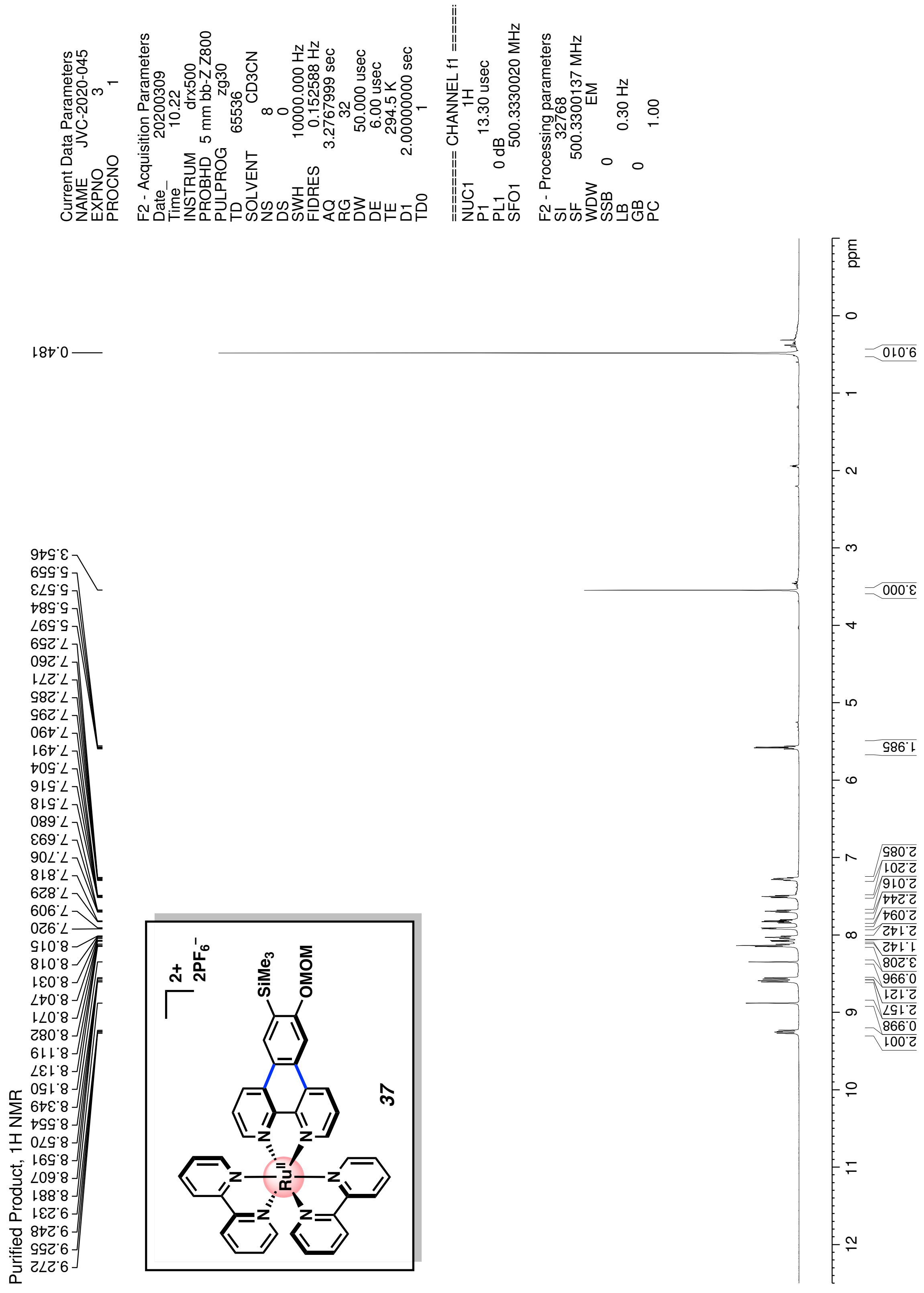

Supplementary Figure 34. ^{1}H NMR (500 MHz, CD_3CN) of 37.

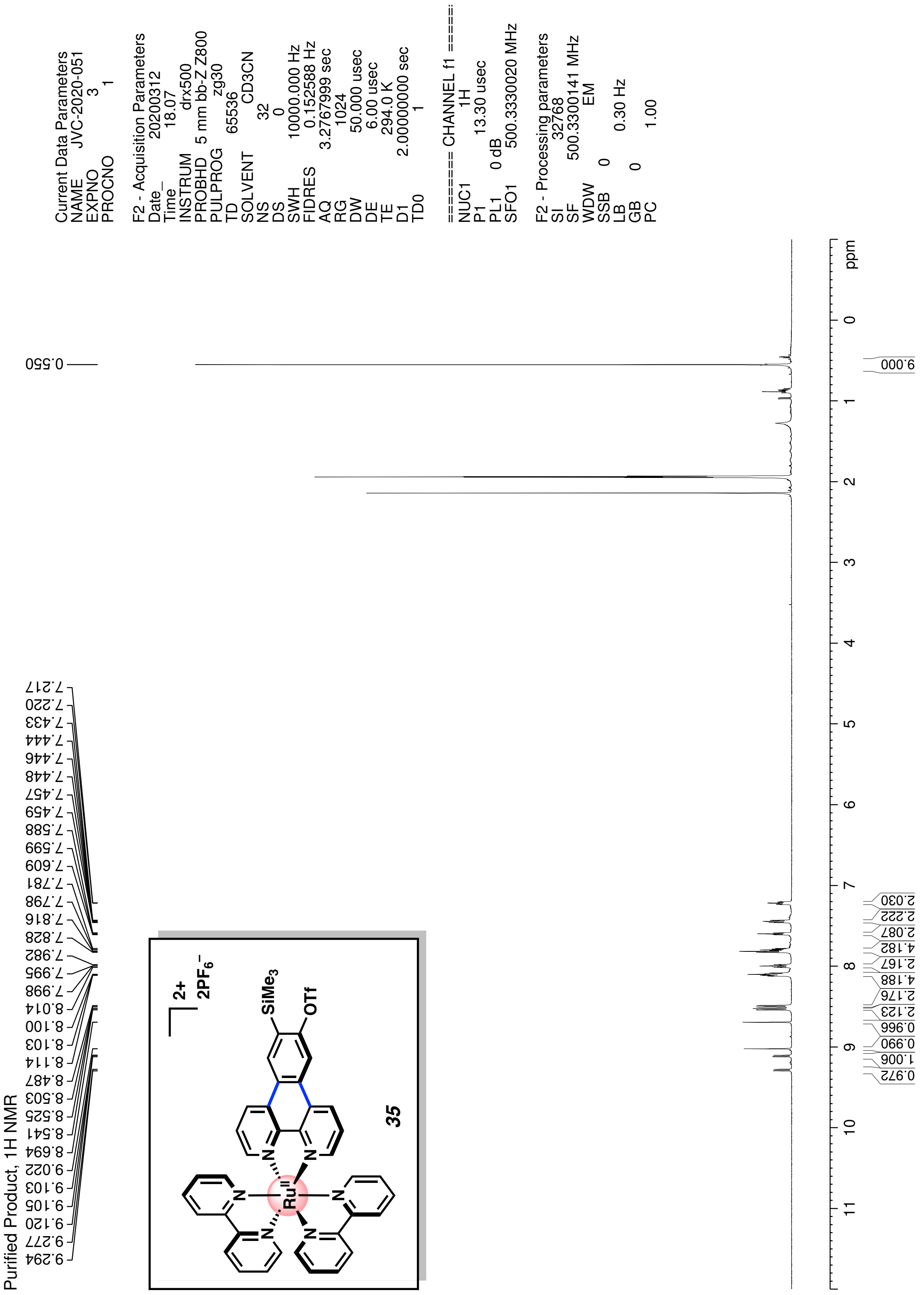

Supplementary Figure 35. ^{1}H NMR (500 MHz, CD_3CN) of 35.

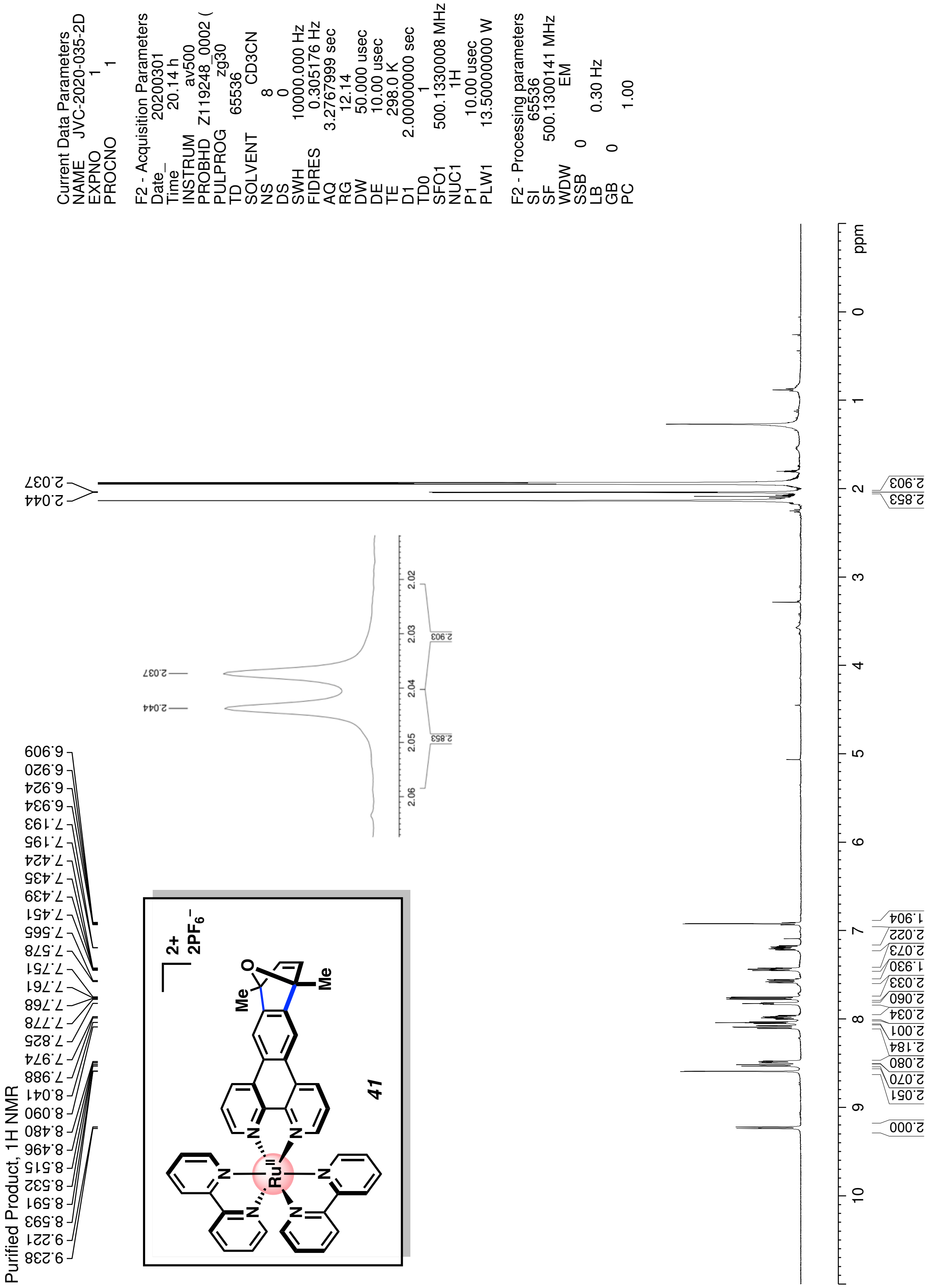

Supplementary Figure 36. ^{1}H NMR (500 MHz, CD_3CN) of 41.

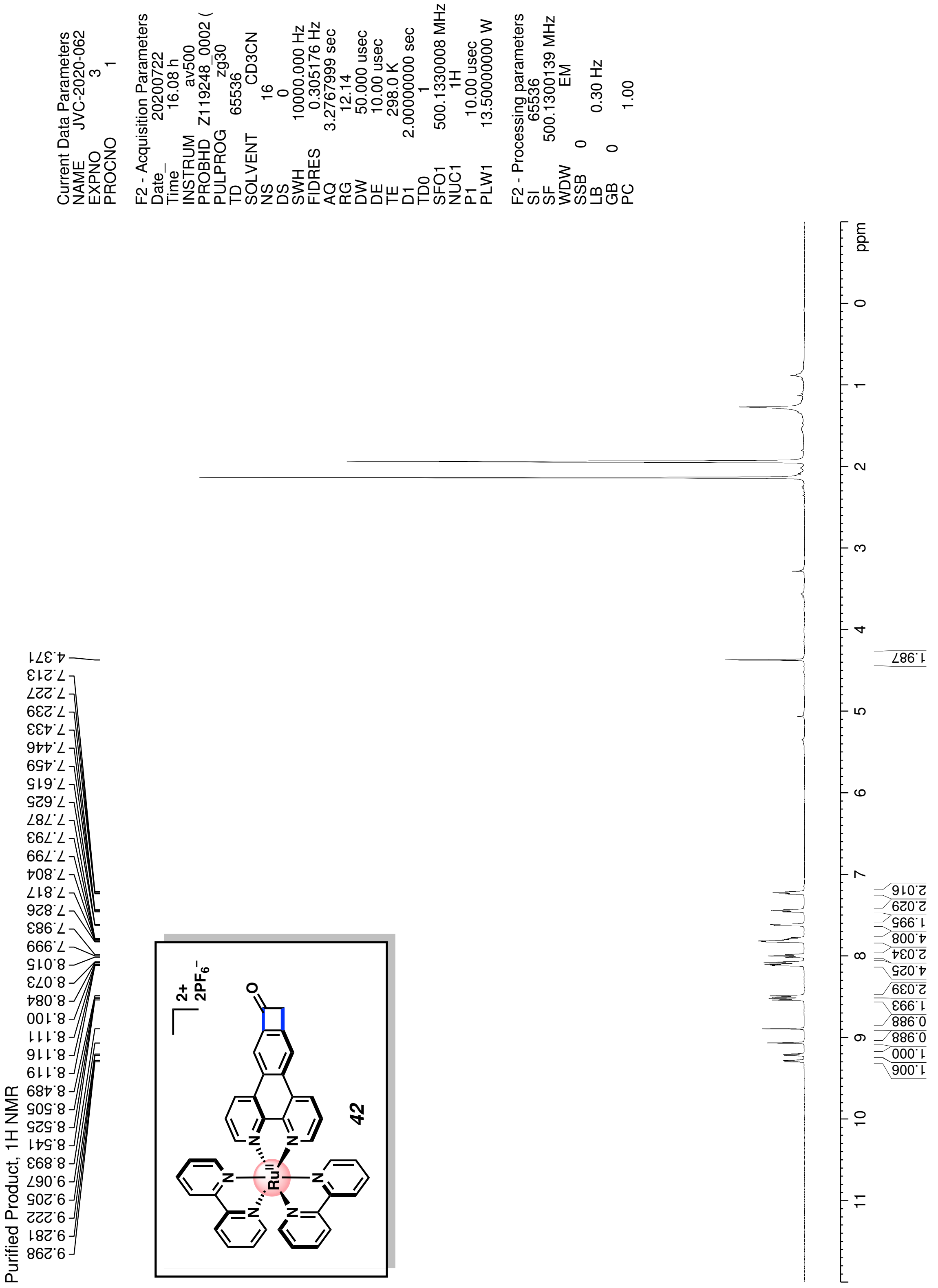

Supplementary Figure 37. ^{1}H NMR (500 MHz, CD_3CN) of 42.

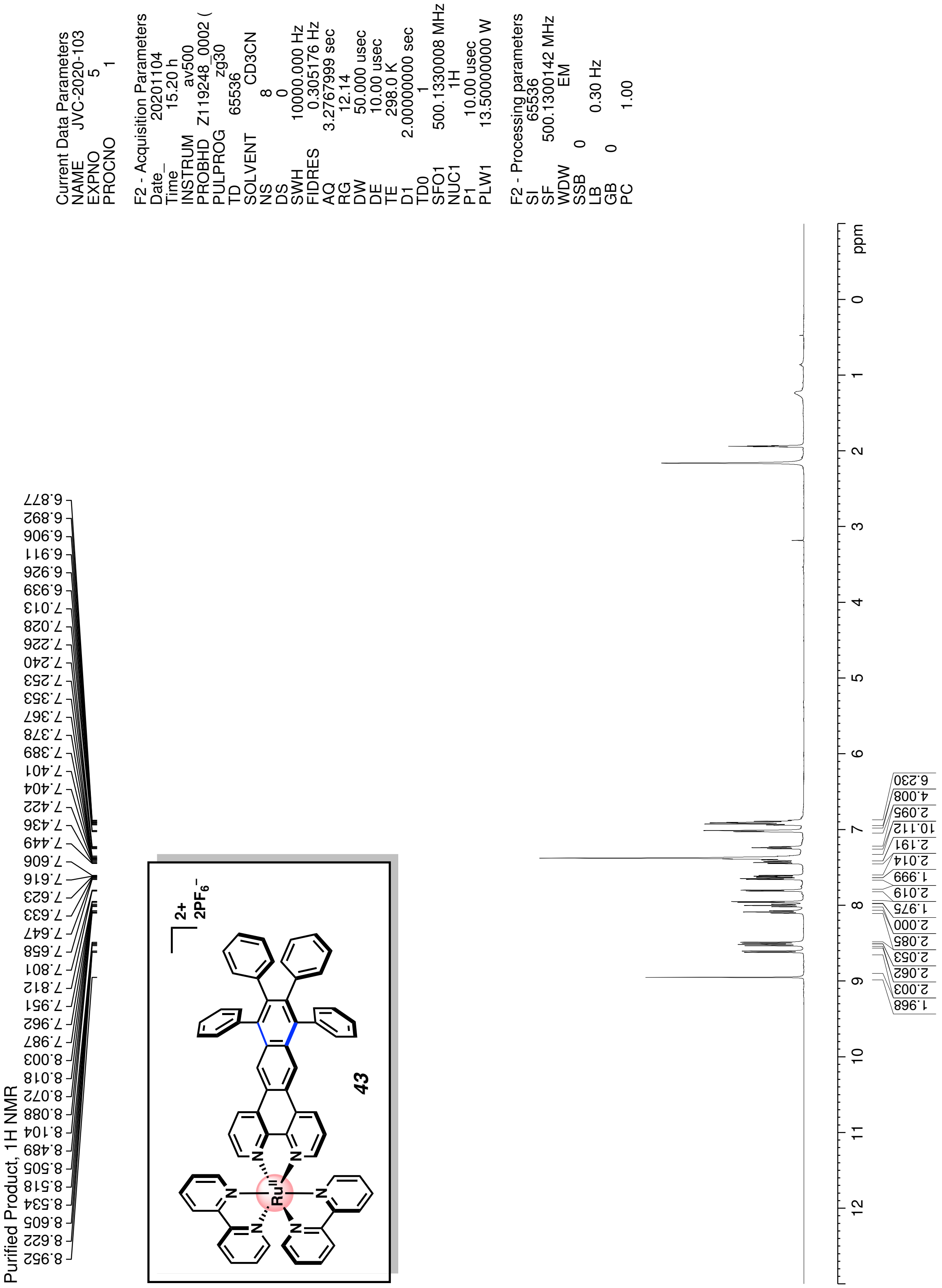

Supplementary Figure 38. ^{1}H NMR (500 MHz, CD_3CN) of 43.

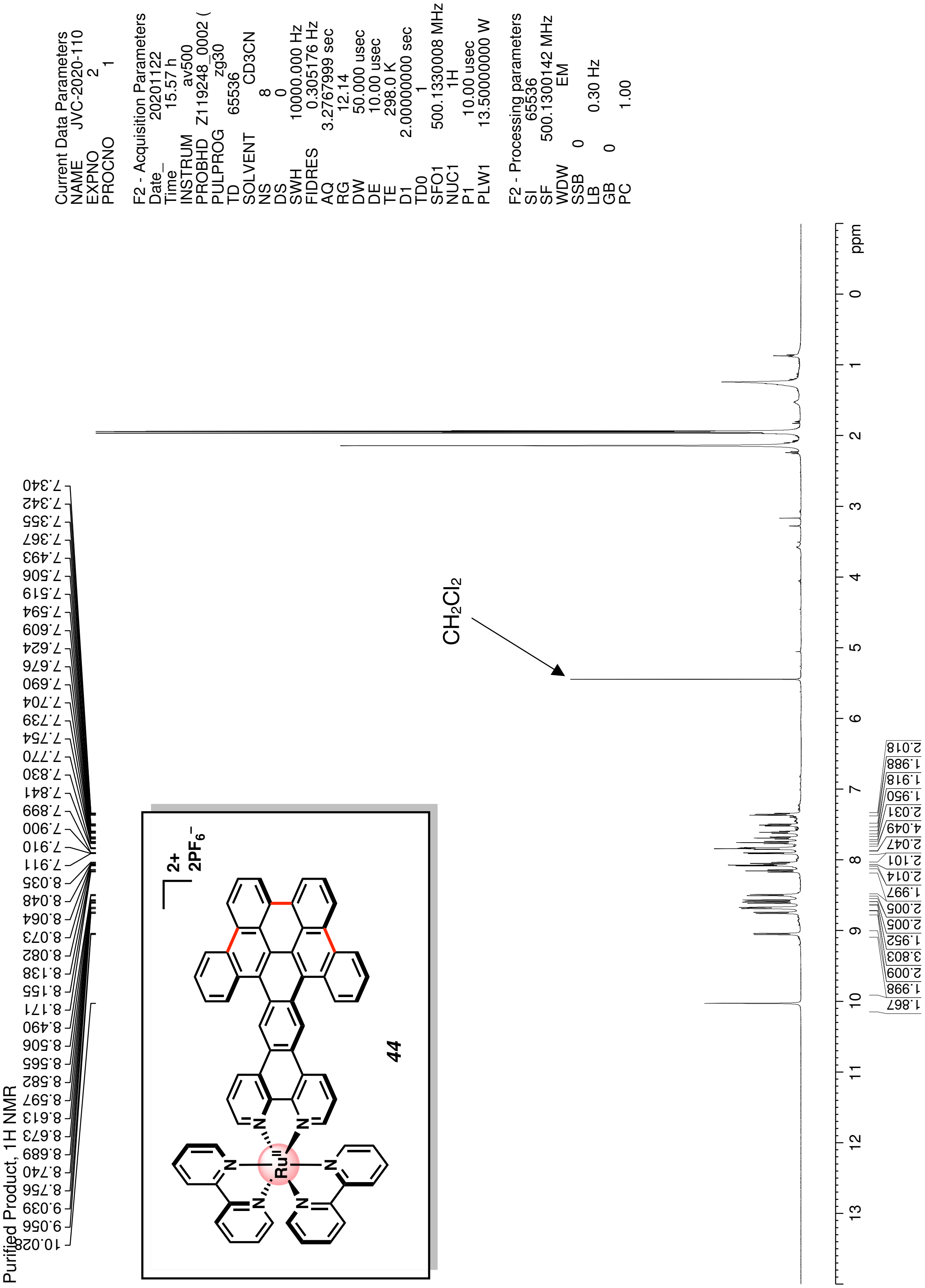

Supplementary Figure 39. ^{1}**H NMR (500 MHz,** CD_3CN**) of 44.**

^{13}C NMR Spectra

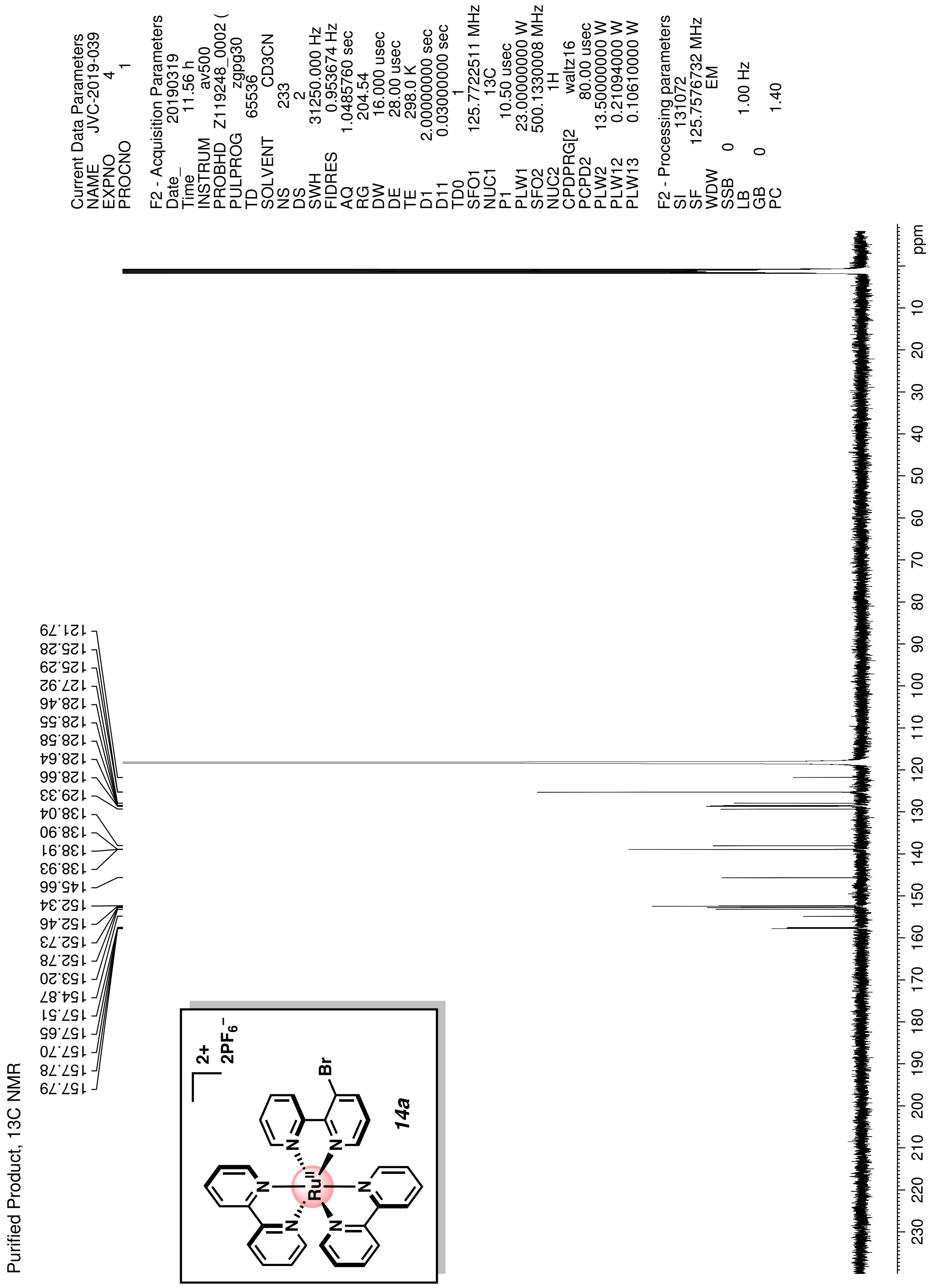

Supplementary Figure 40. **^{13}C NMR (125 MHz, CD_3CN) of 14a.**

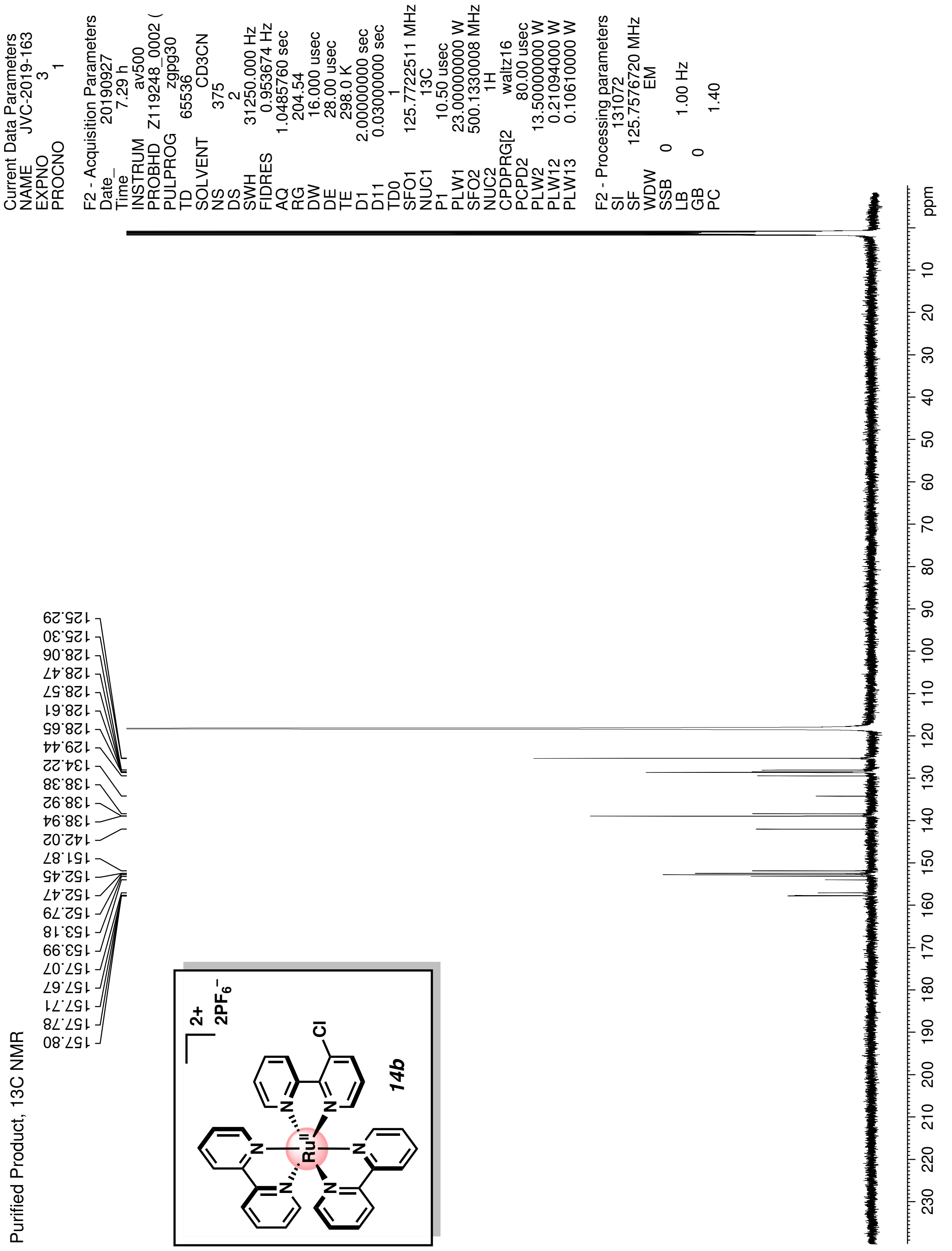

Supplementary Figure 41. ^{13}**C NMR (125 MHz, CD_3CN) of 14b.**

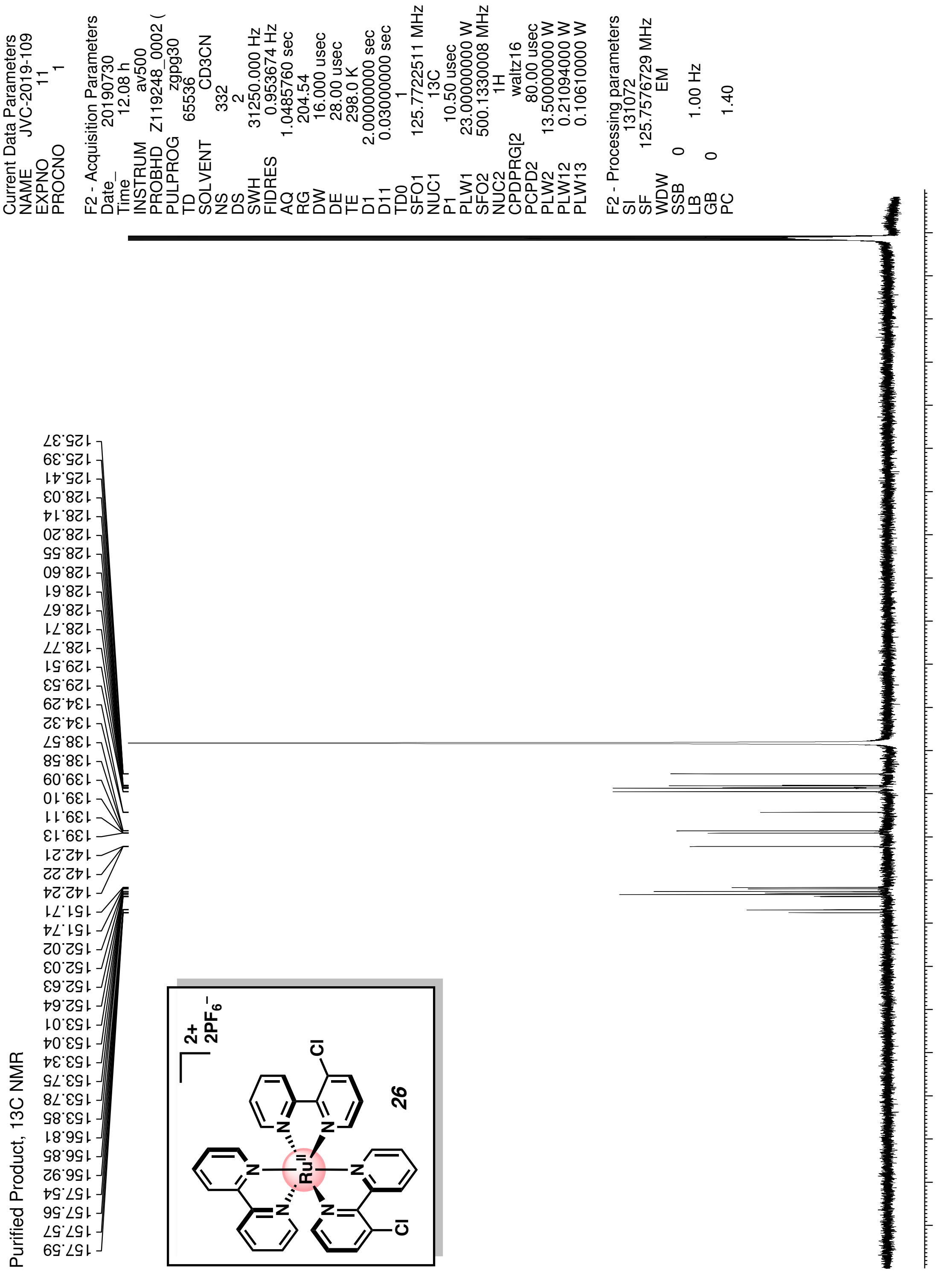

Supplementary Figure 42. ^{13}**C NMR (125 MHz, CD$_3$CN) of 26.**

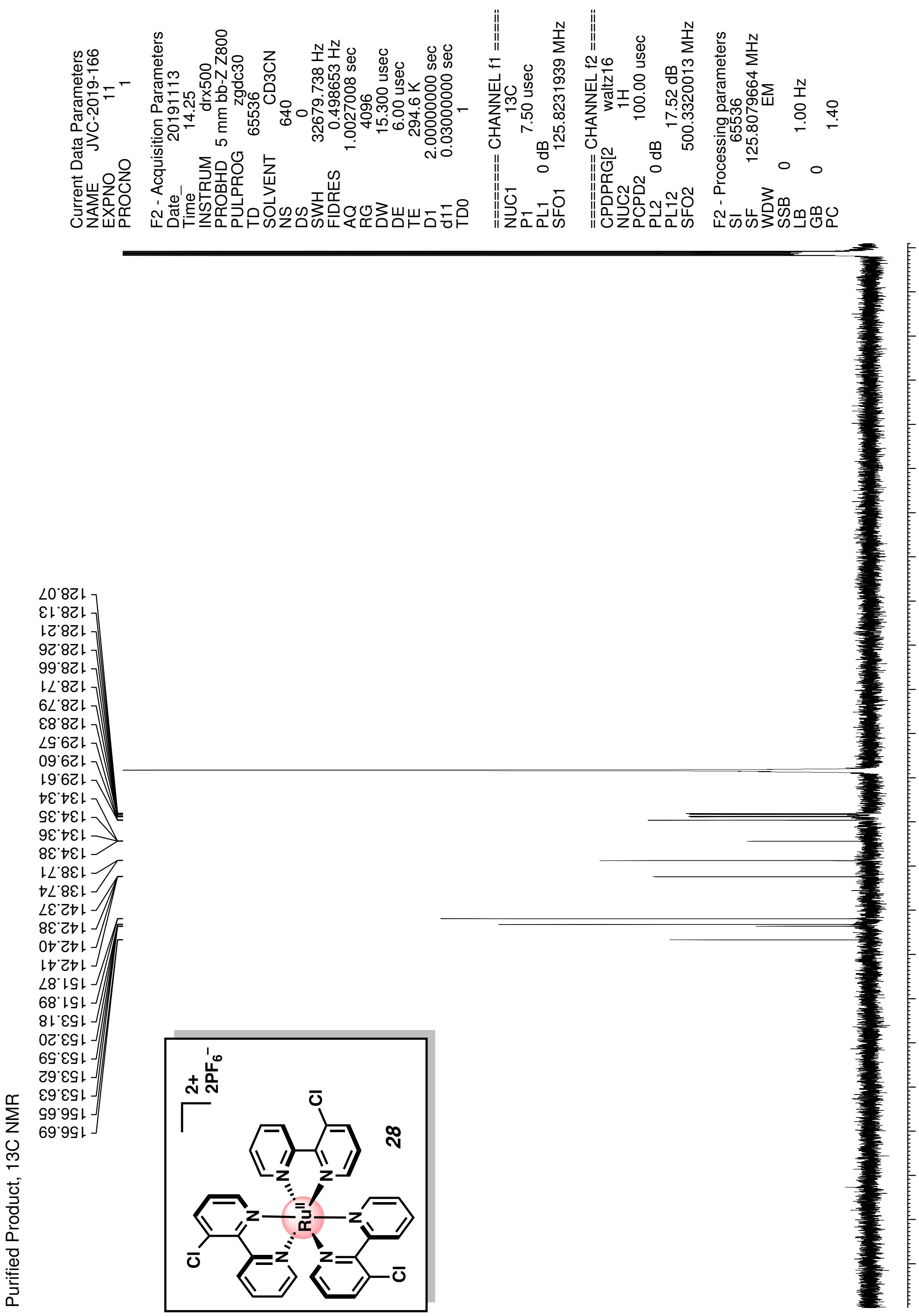

Supplementary Figure 43. ^{13}**C NMR (125 MHz, CD**$_3$**CN) of 28.**

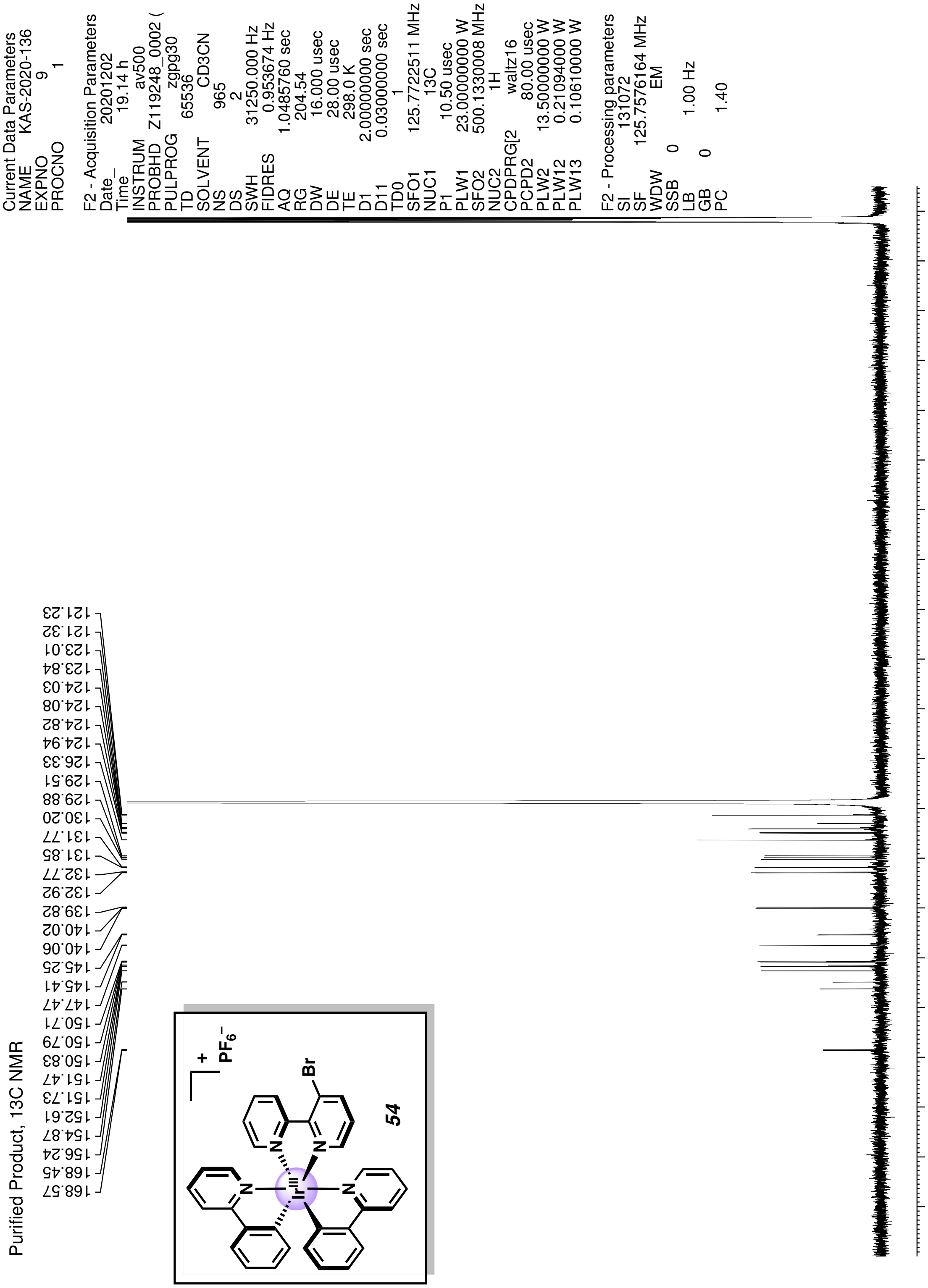

Supplementary Figure 44. ^{13}C NMR (125 MHz, CD_3CN) of 54.

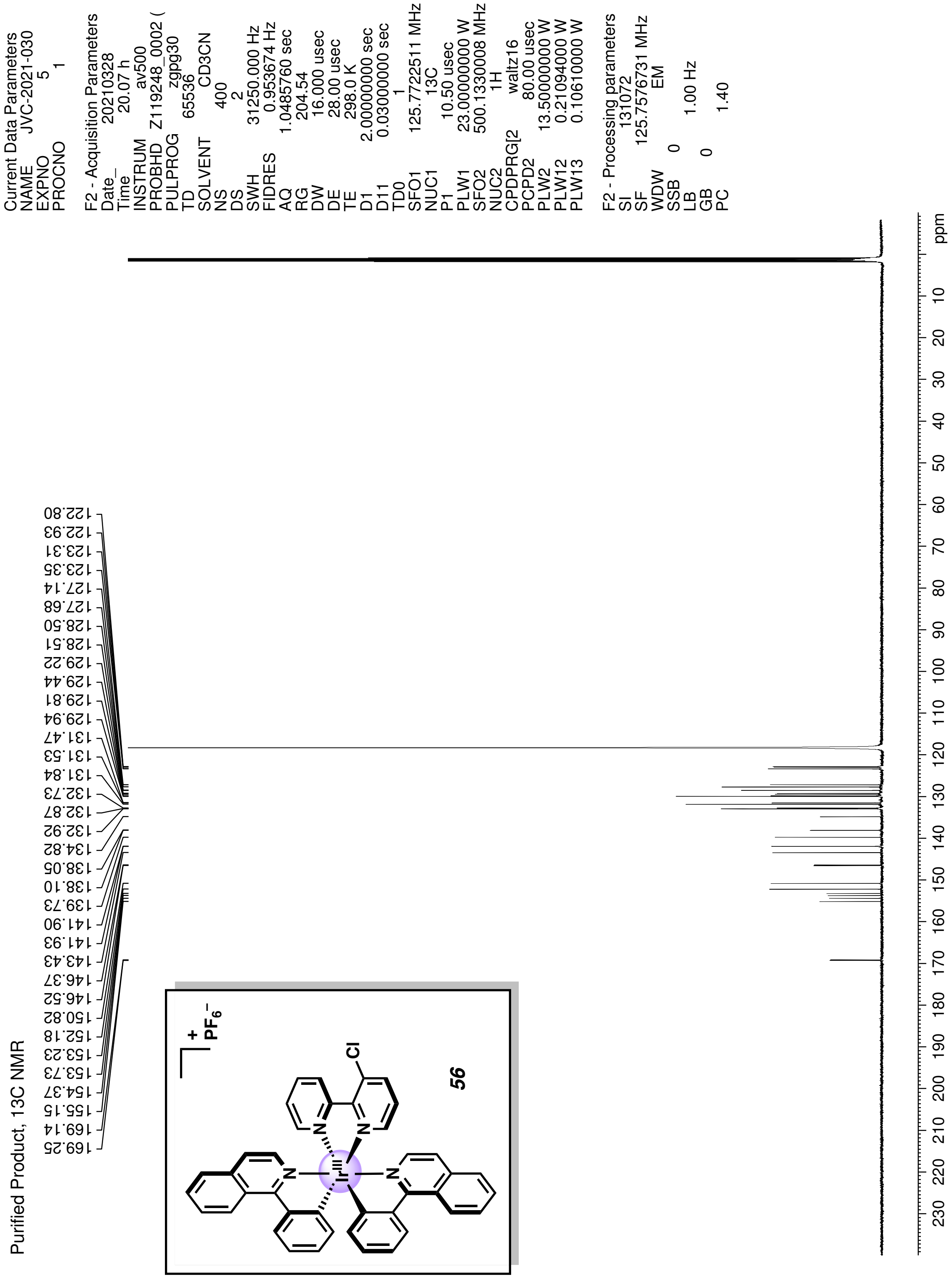

Supplementary Figure 45. ^{13}C NMR (125 MHz, CD_3CN) of 56.

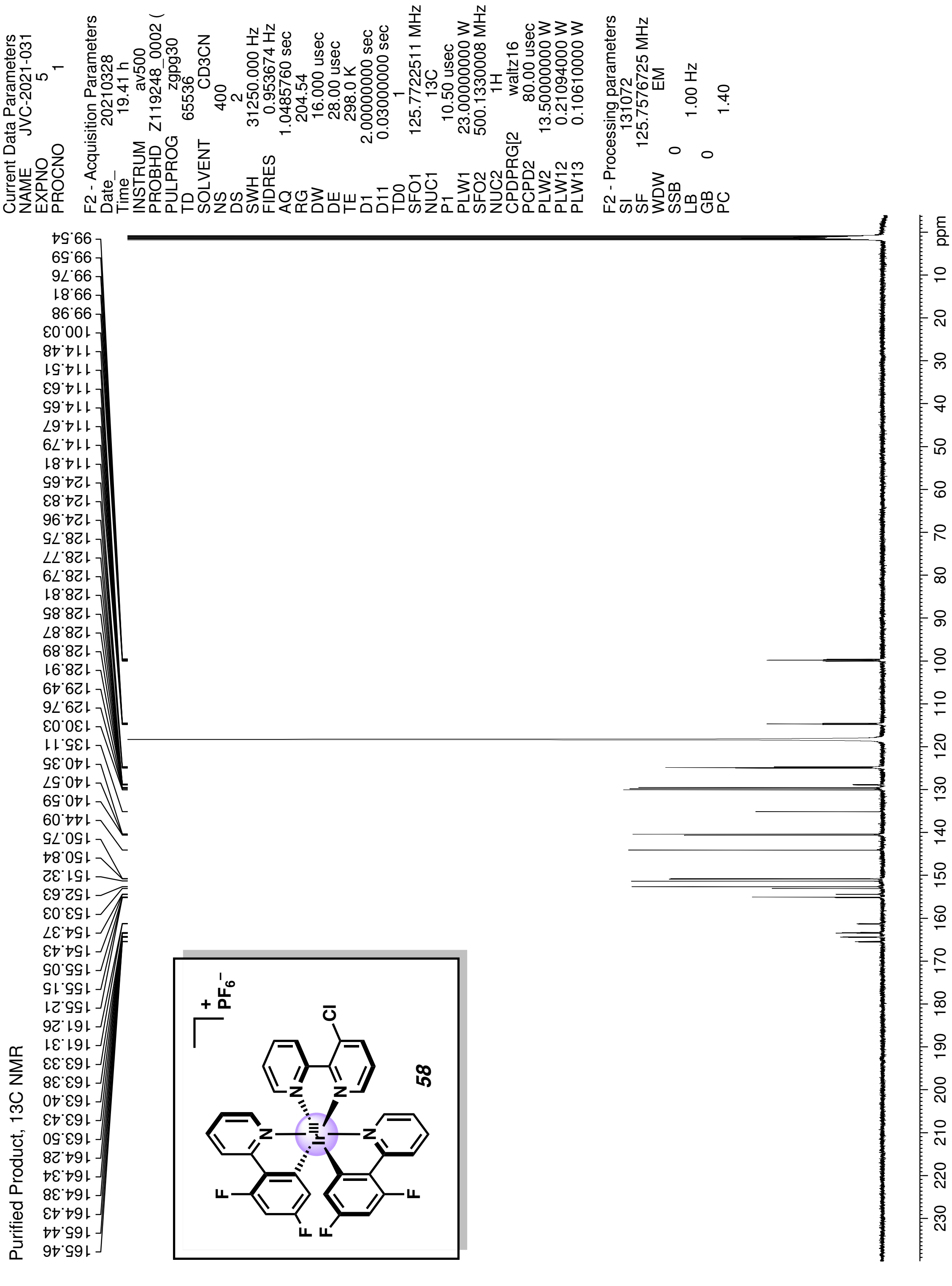

Supplementary Figure 46. ^{13}C NMR (125 MHz, CD_3CN) of 58.

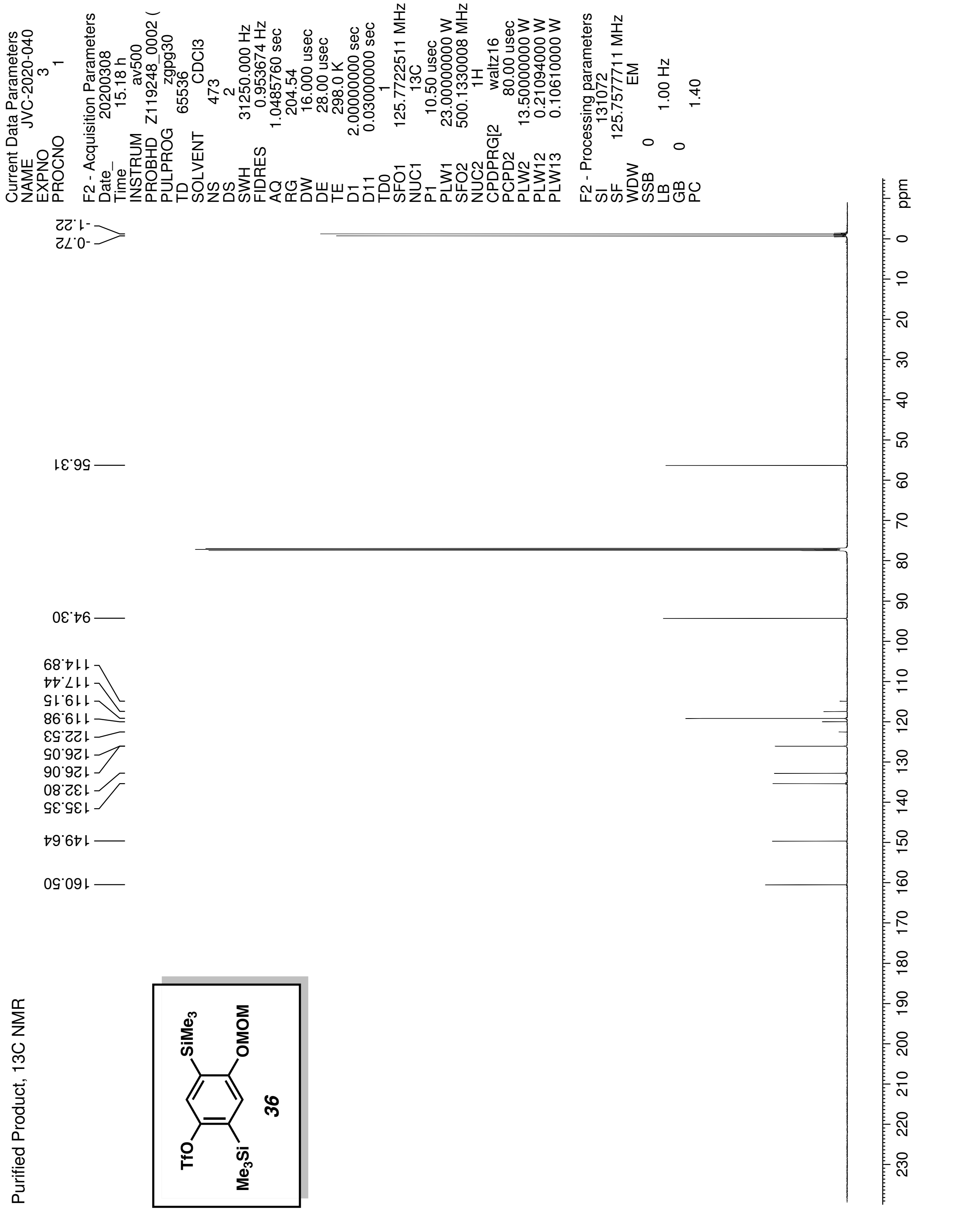

Supplementary Figure 47. ^{13}**C NMR (125 MHz, CDCl$_3$) of 36.**

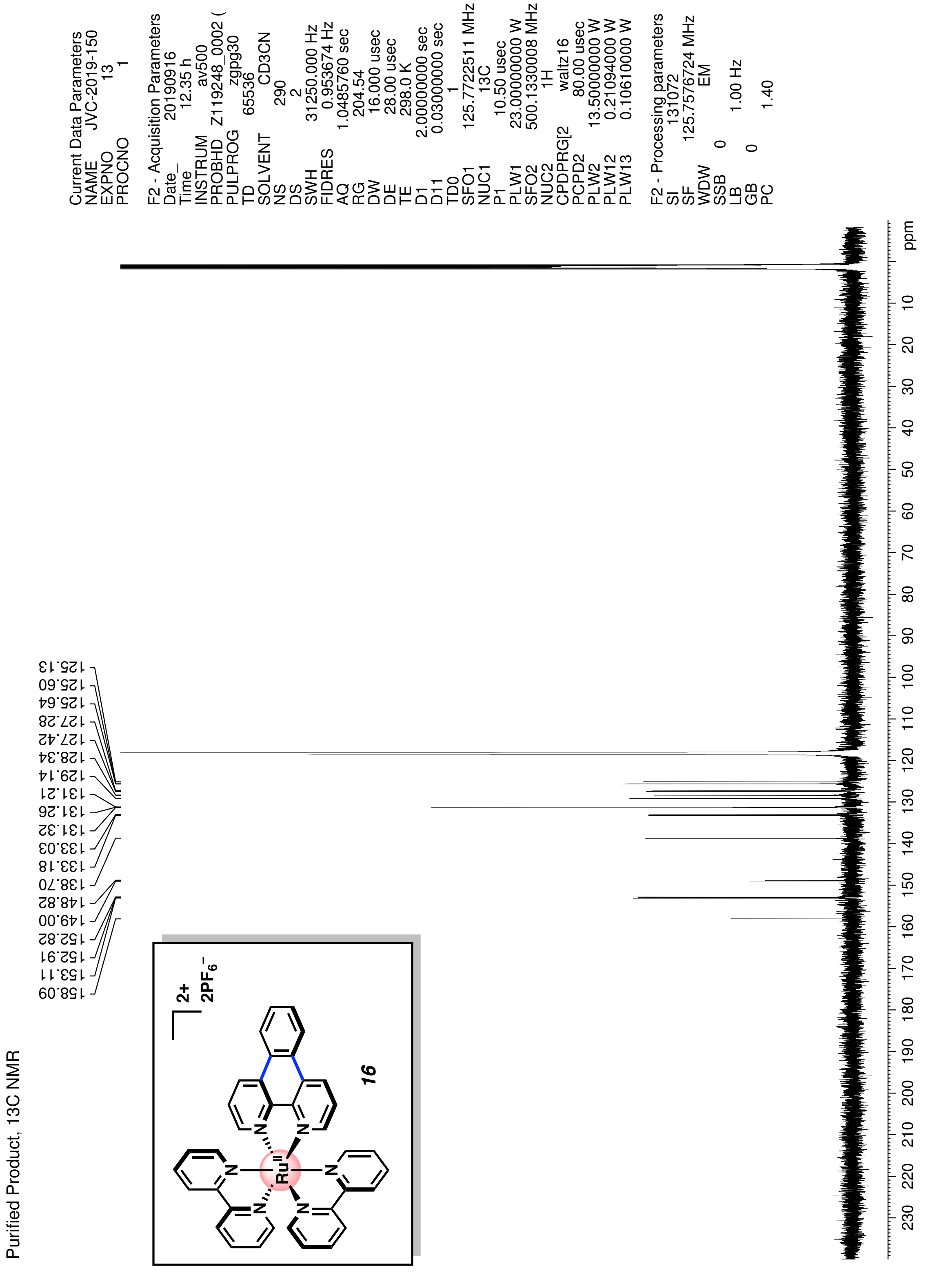

Supplementary Figure 48. **^{13}C NMR (125 MHz, CD_3CN) of 16.**

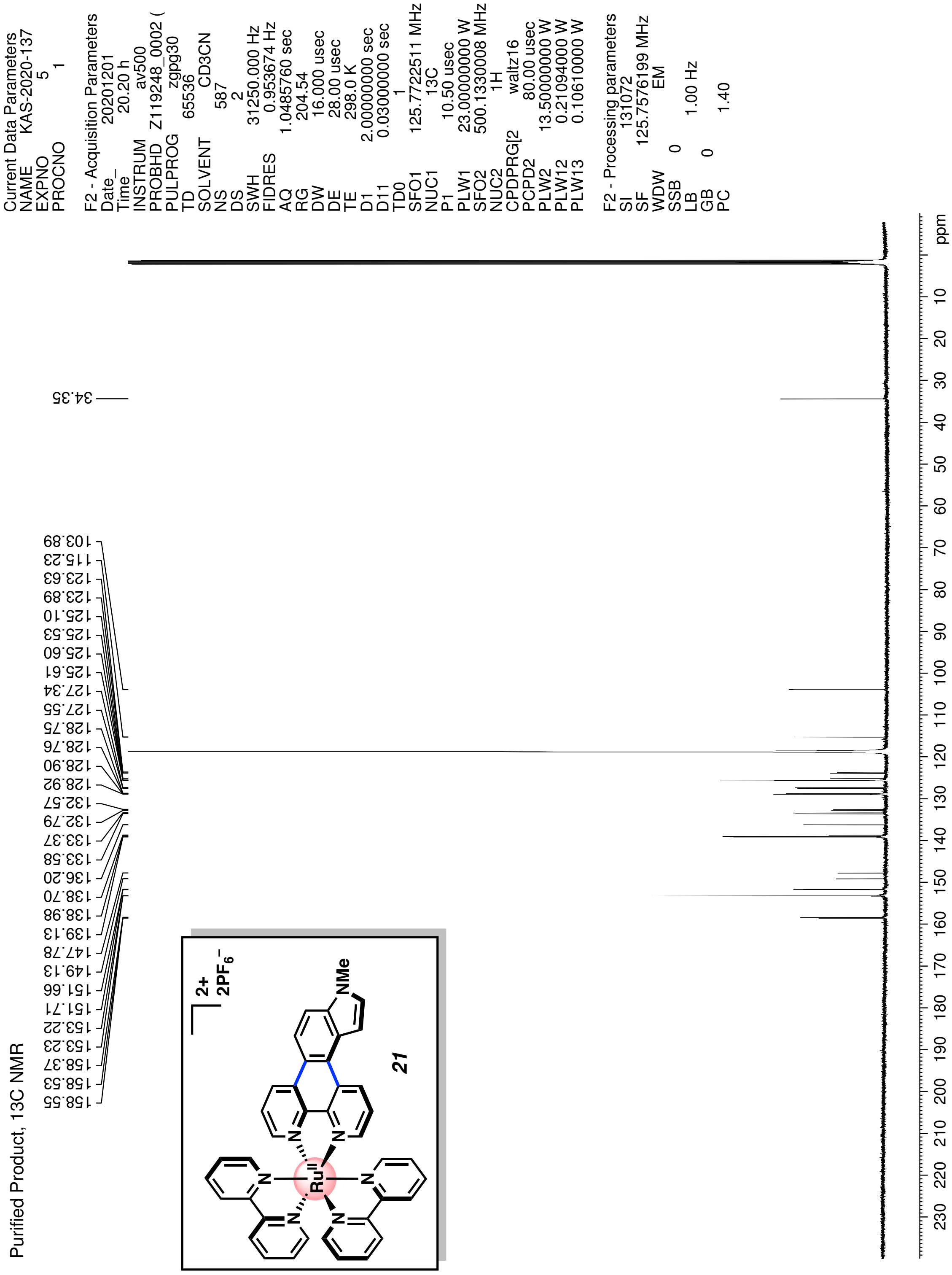

Supplementary Figure 49. ^{13}C NMR (125 MHz, CD_3CN) of 21.

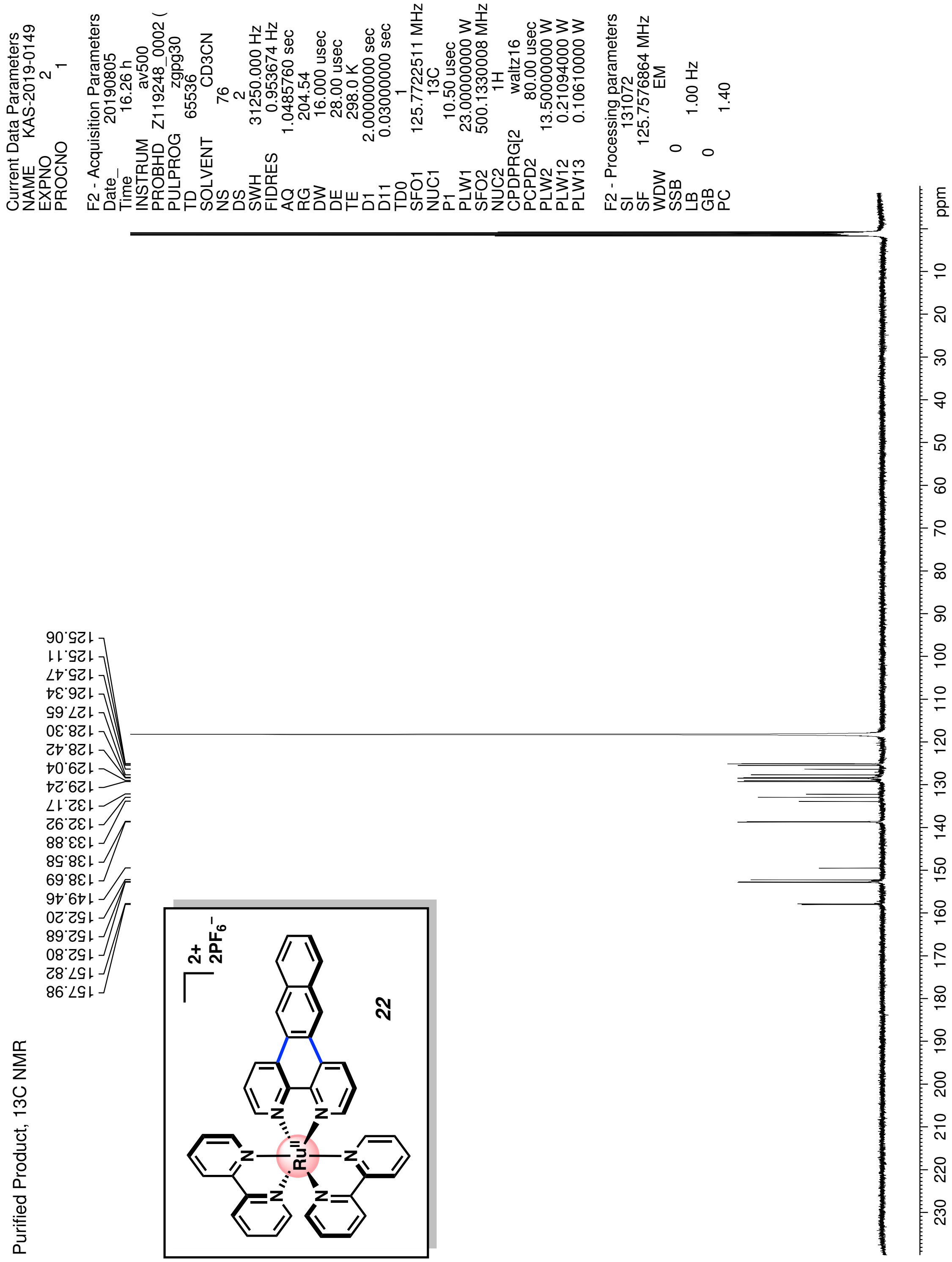

Supplementary Figure 50. ^{13}C NMR (125 MHz, CD_3CN) of 22.

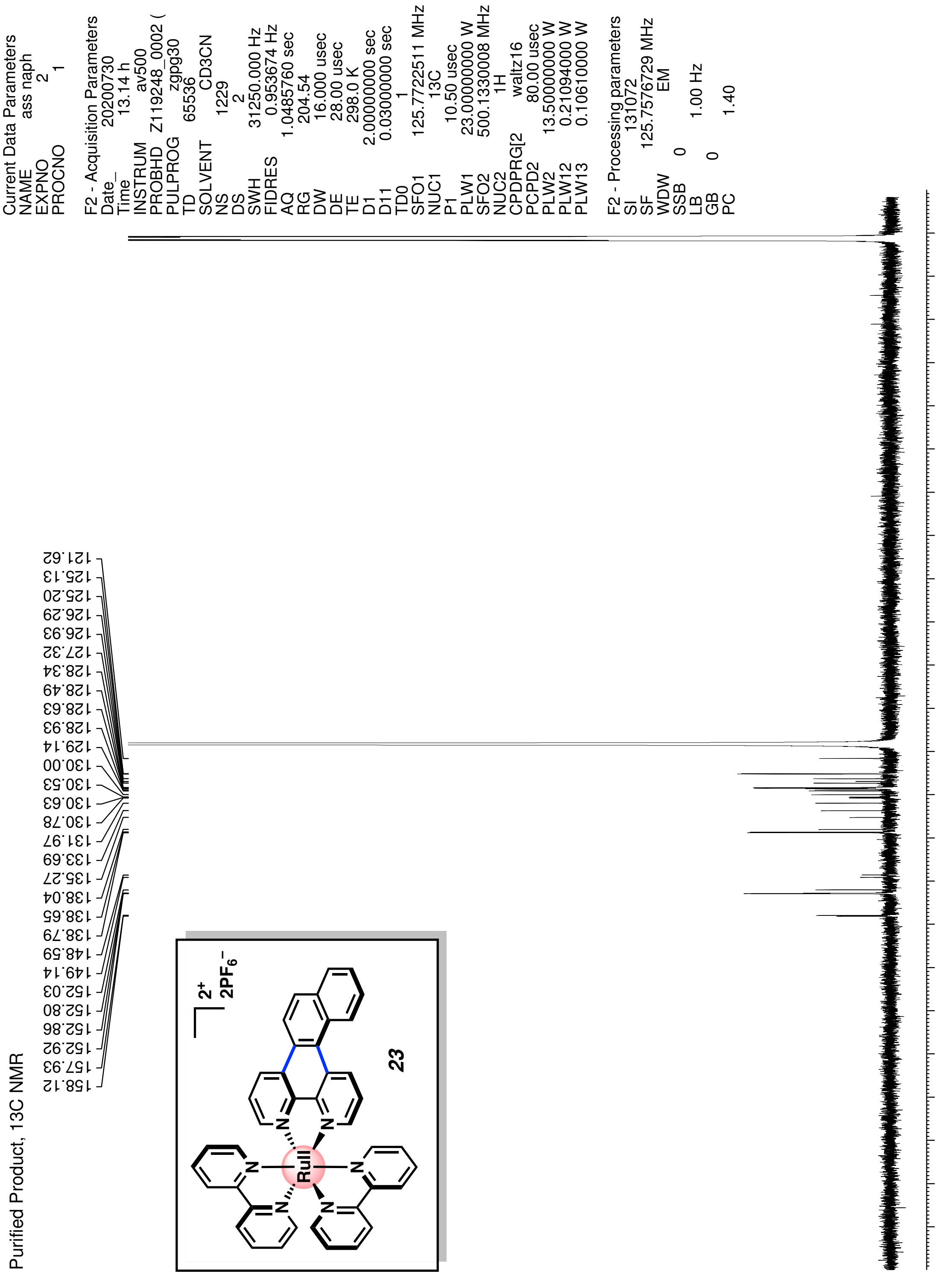

Supplementary Figure 51. ^{13}C NMR (125 MHz, CD_3CN) of 23.

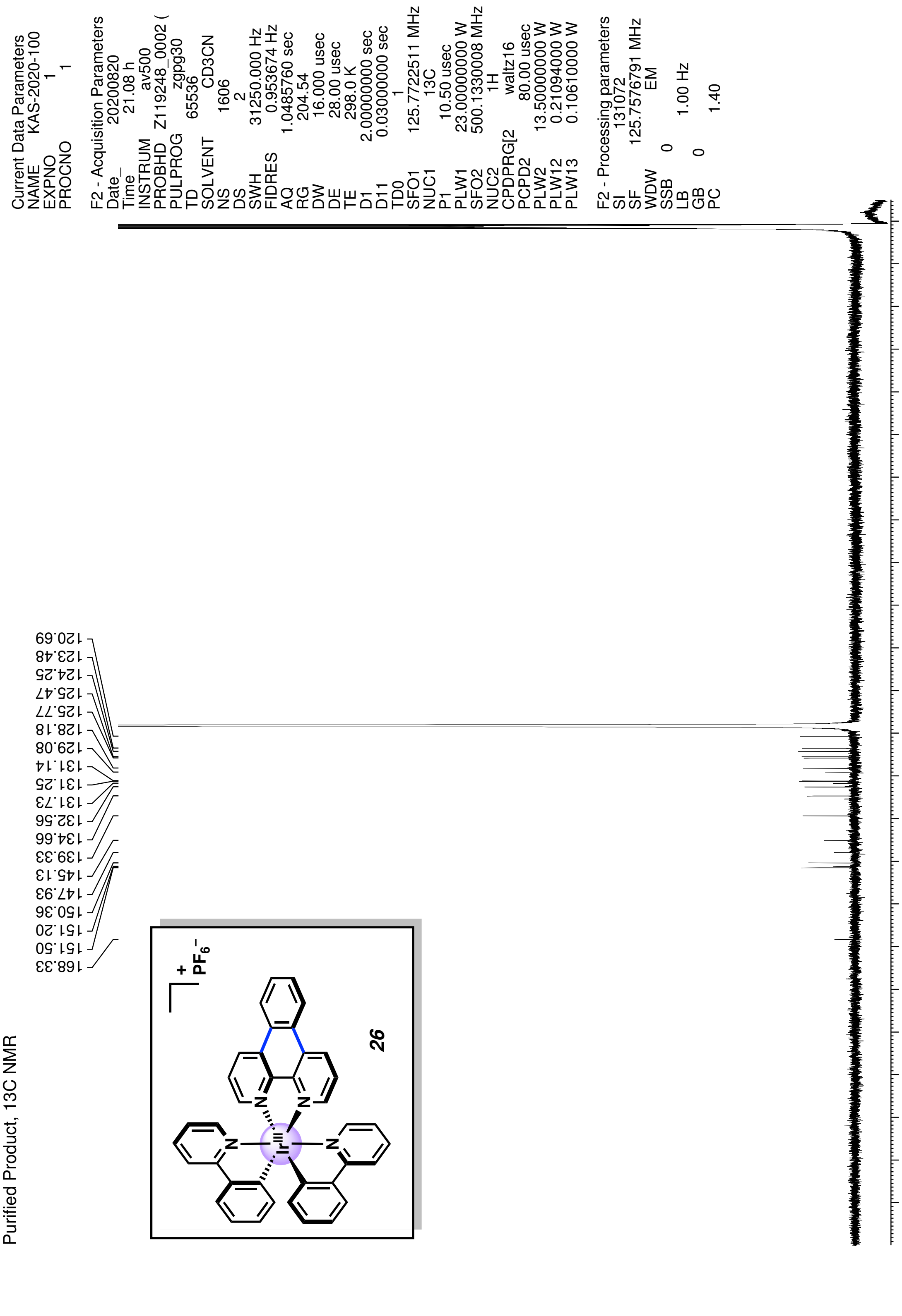

Supplementary Figure 52. **^{13}C NMR (125 MHz, CD_3CN) of 26.**

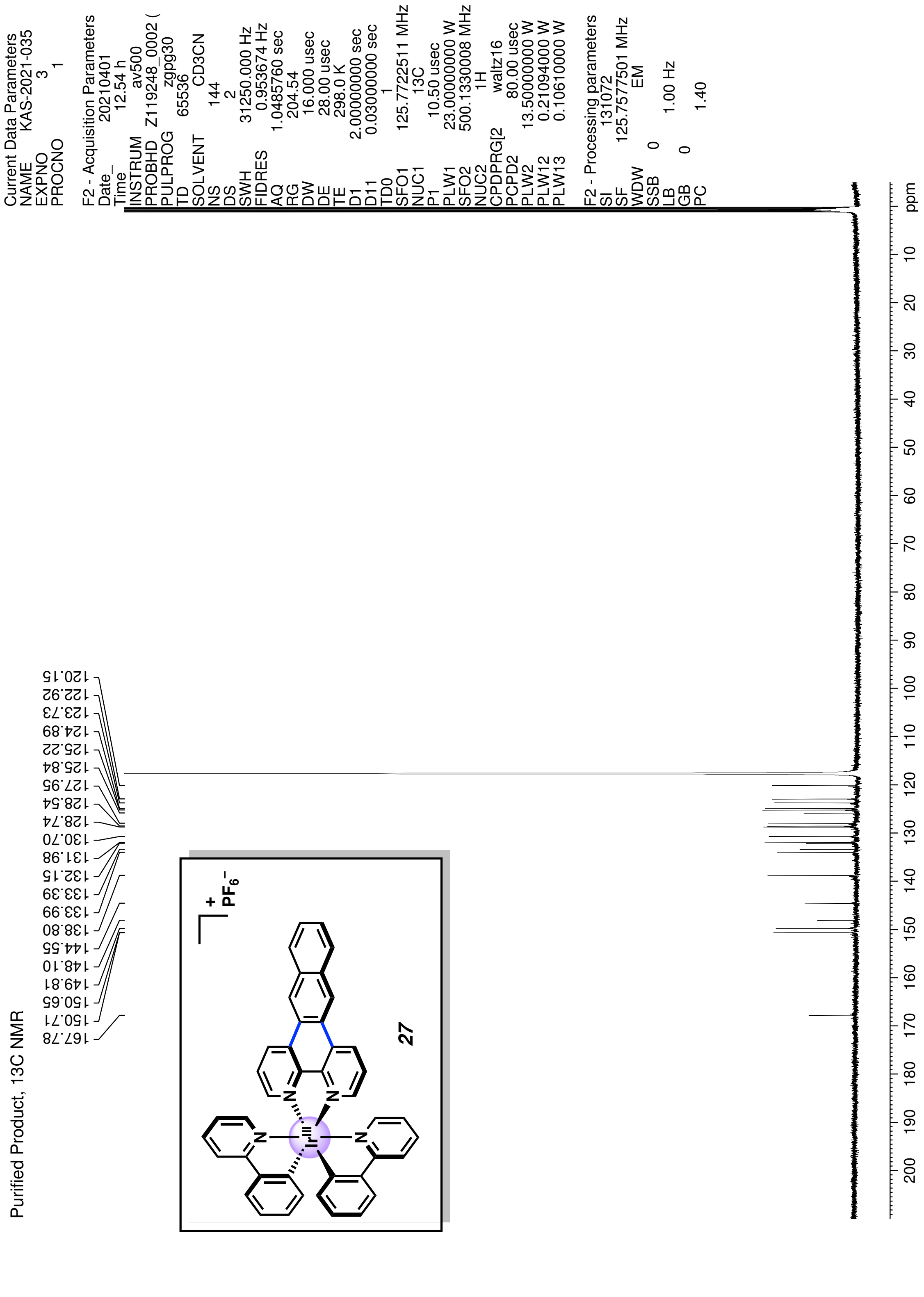

Supplementary Figure 53. ^{13}C NMR (125 MHz, CD_3CN) of 27.

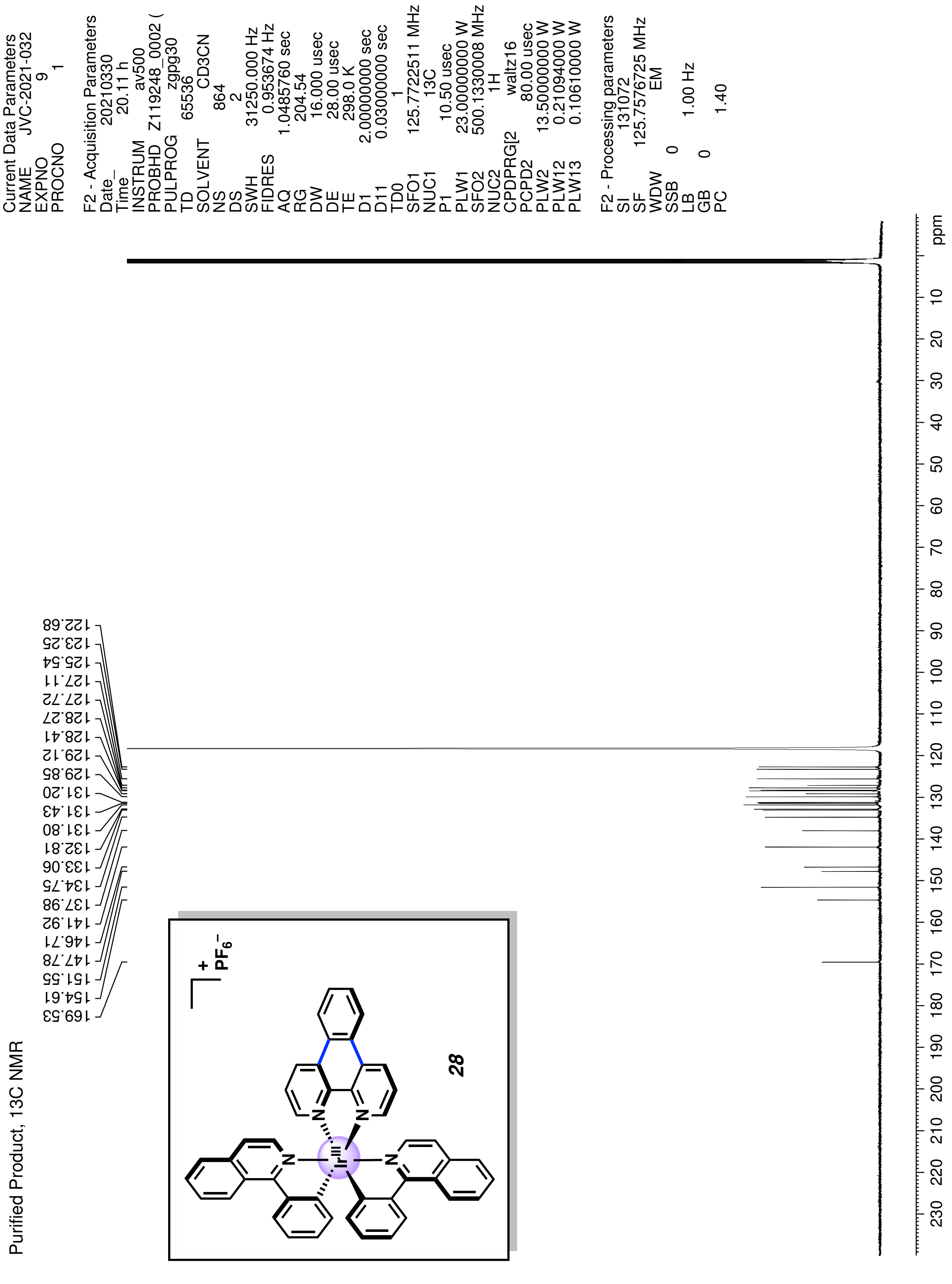

Supplementary Figure 54. ^{13}C NMR (125 MHz, CD_3CN) of 28.

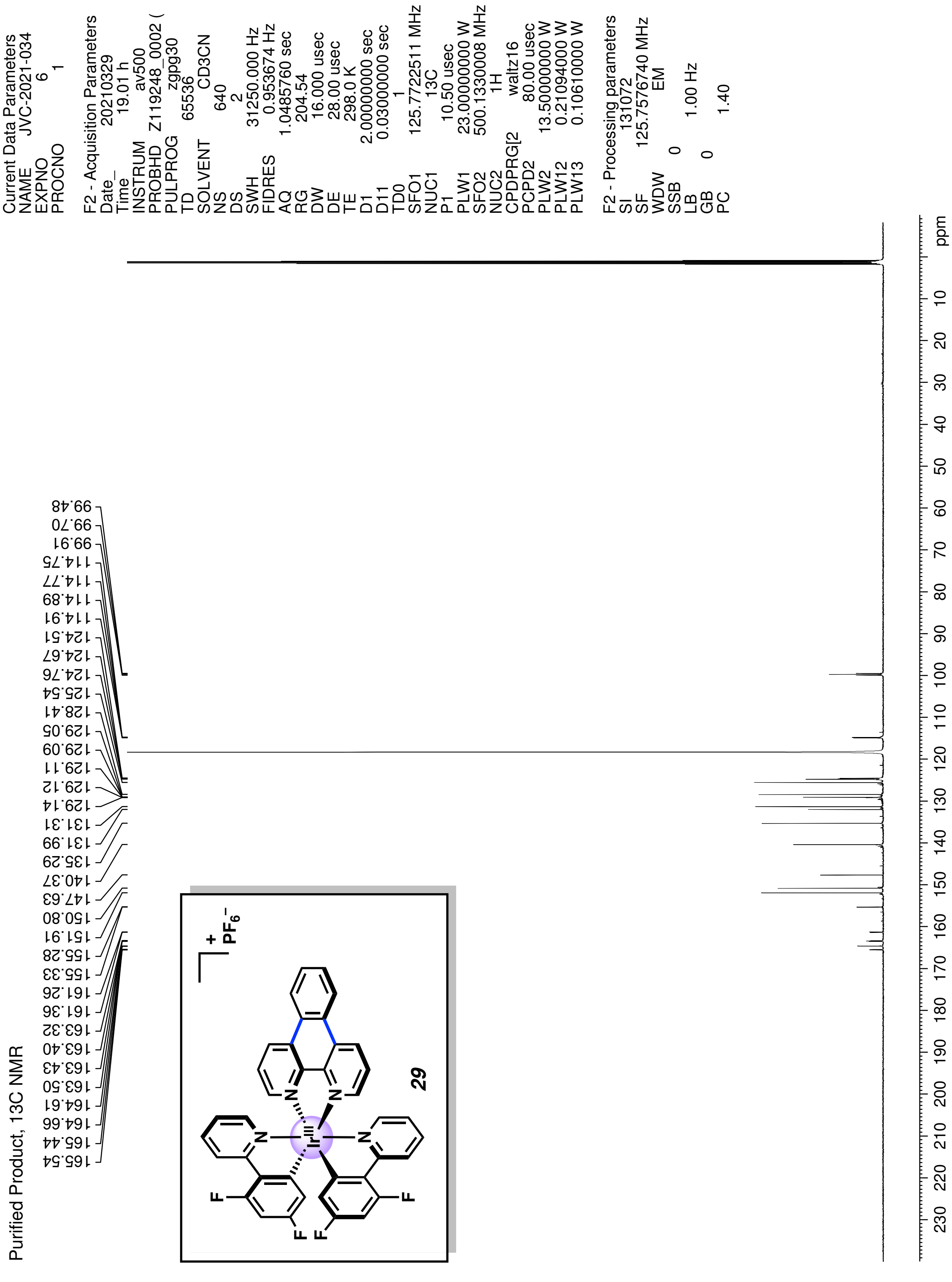

Supplementary Figure 55. ^{13}C NMR (125 MHz, CD_3CN) of 29.

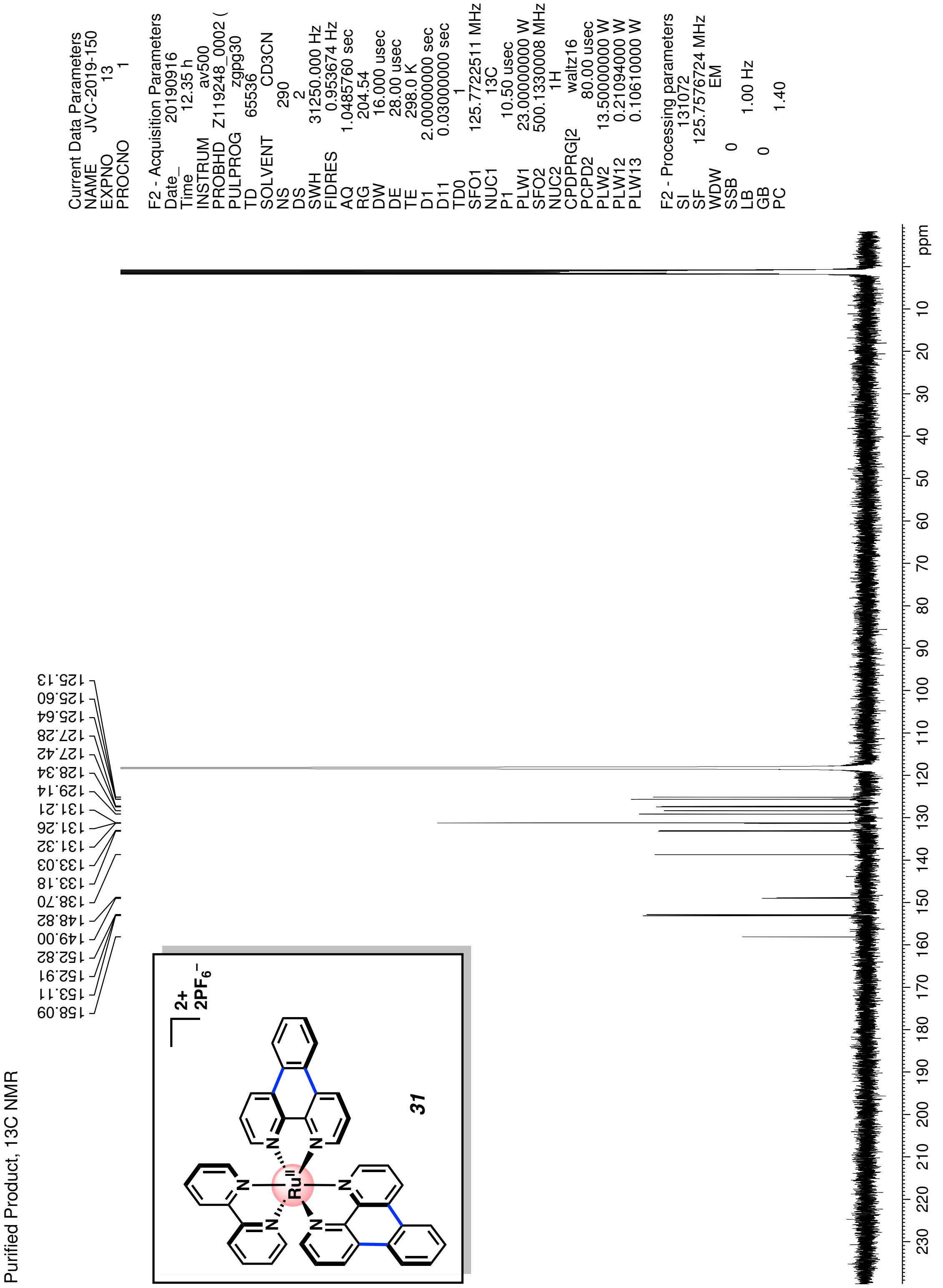

Supplementary Figure 56. ^{13}C NMR (125 MHz, CD_3CN) of 31.

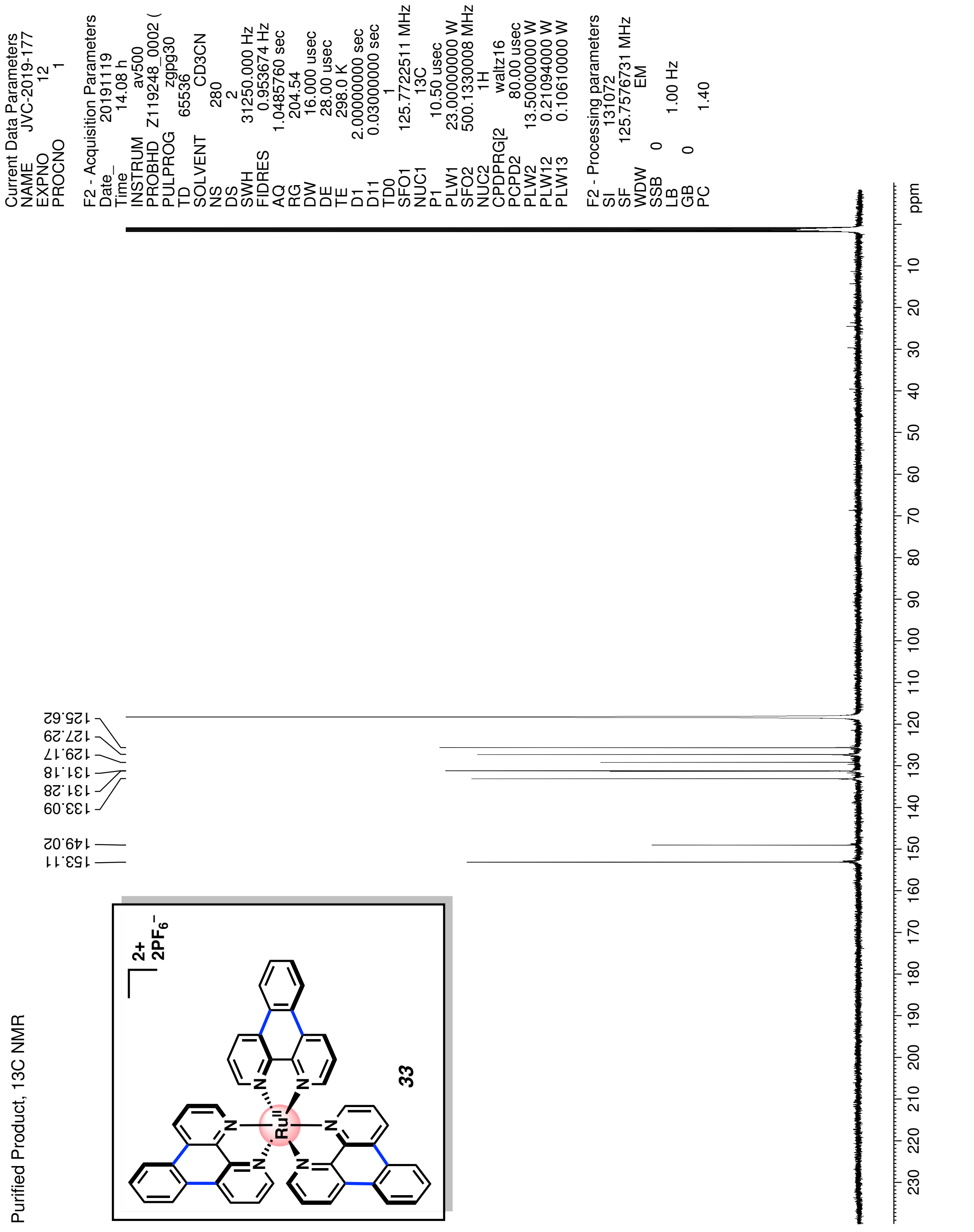

Supplementary Figure 57. ^{13}C NMR (125 MHz, CD_3CN) of 33.

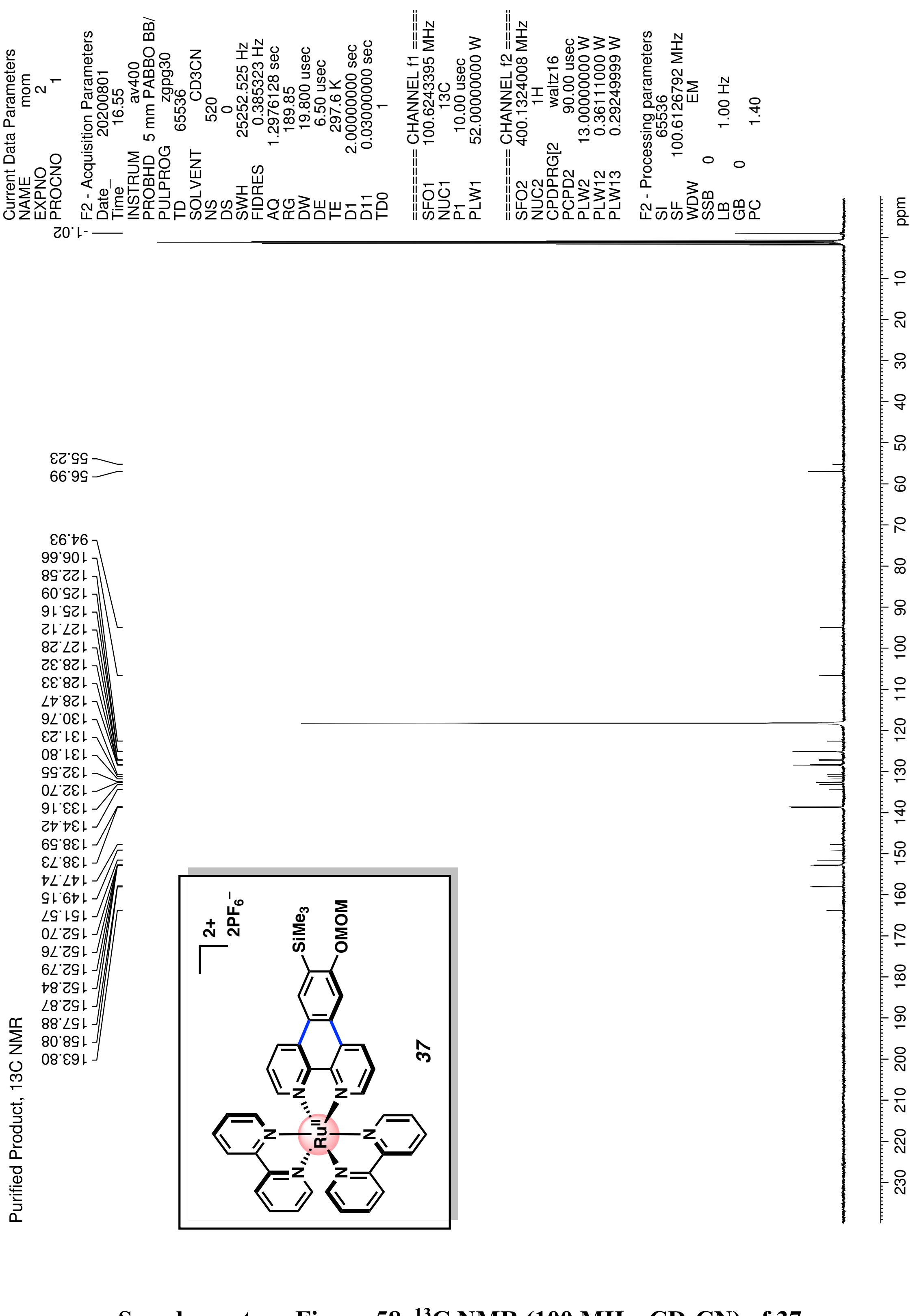

Supplementary Figure 58. ^{13}**C NMR (100 MHz, CD**$_3$**CN) of 37.**

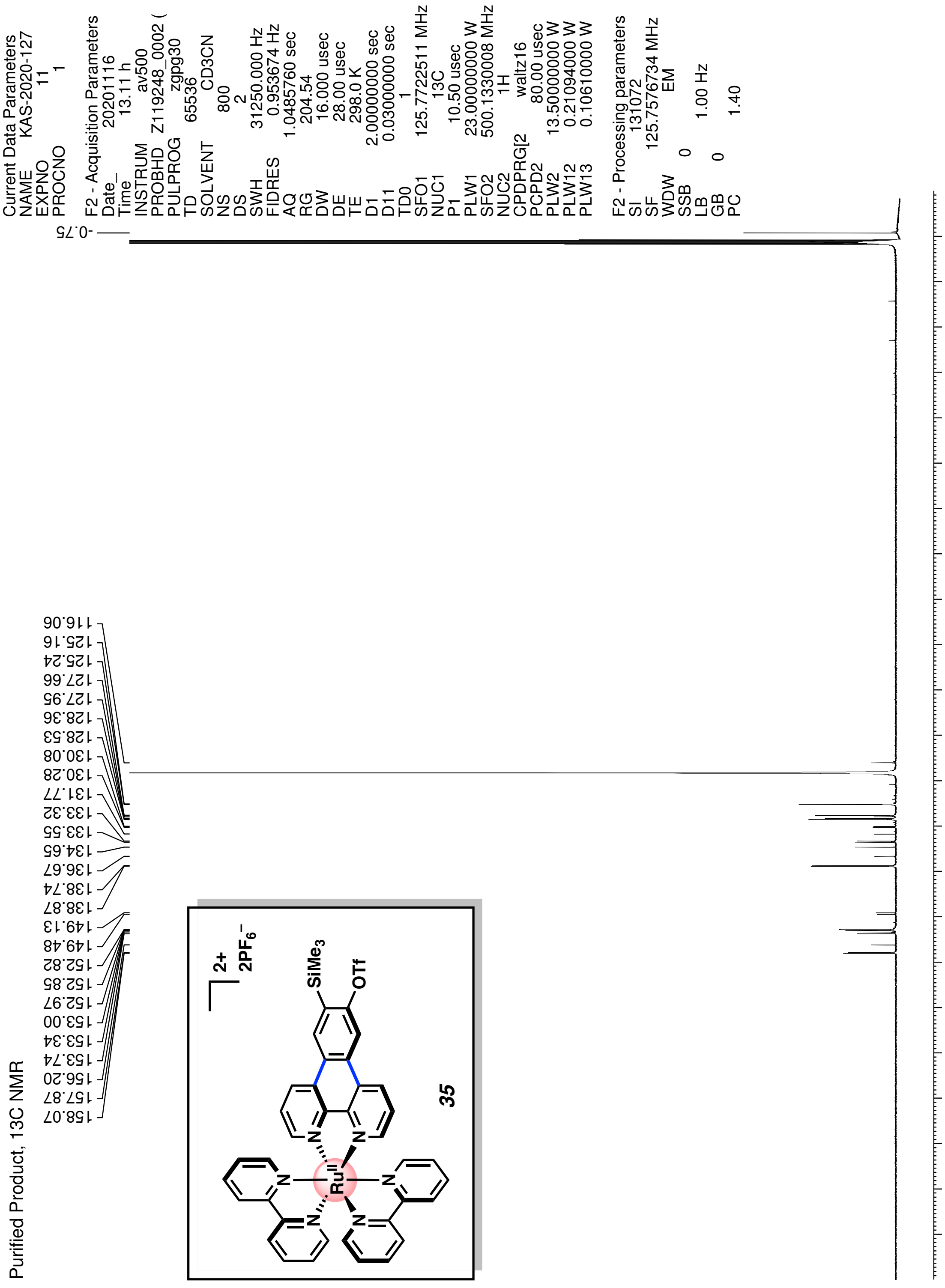

Supplementary Figure 59. ^{13}C NMR (125 MHz, CD_3CN) of 35.

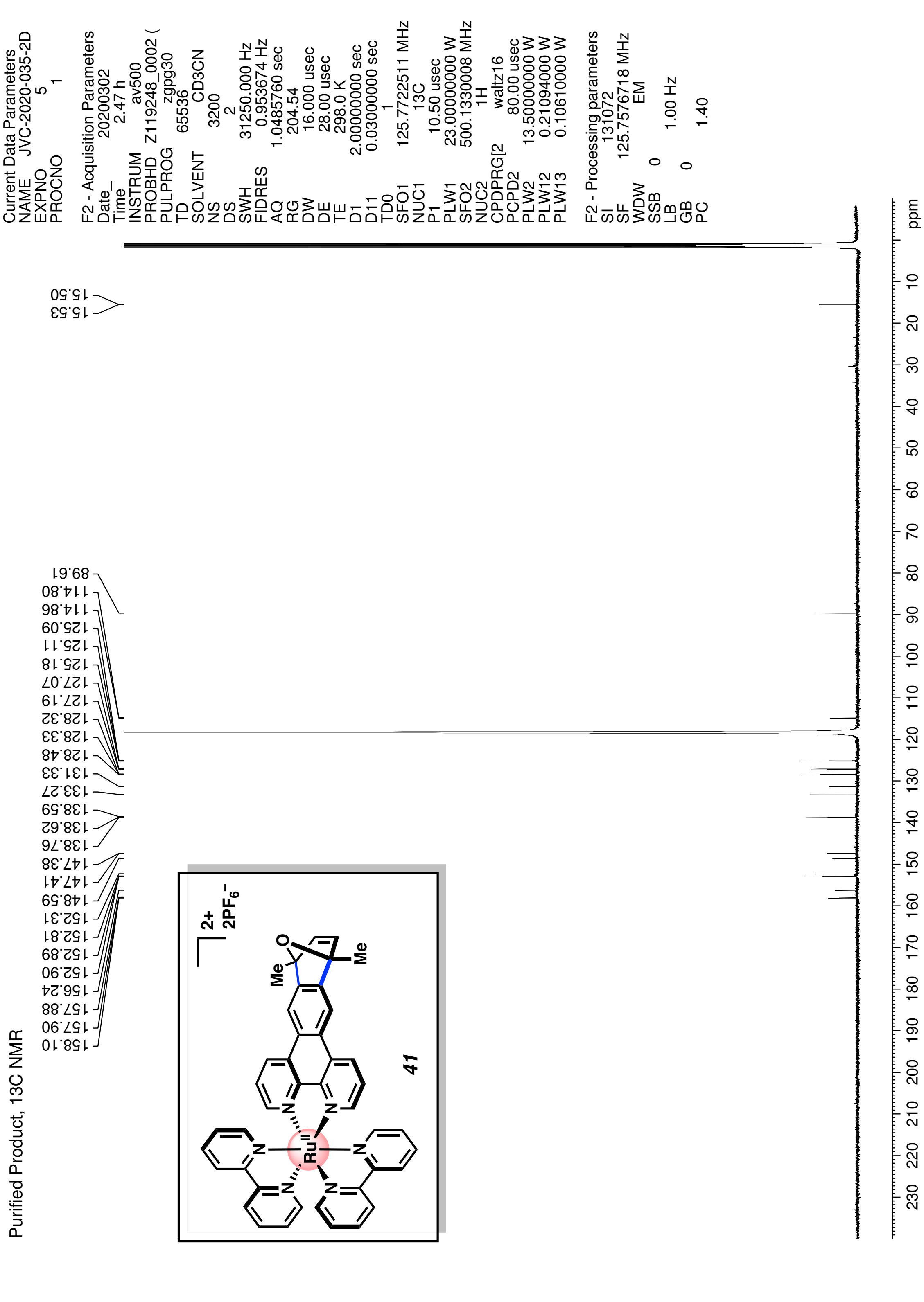

Supplementary Figure 60. ^{13}**C NMR (125 MHz, CD$_3$CN) of 41.**

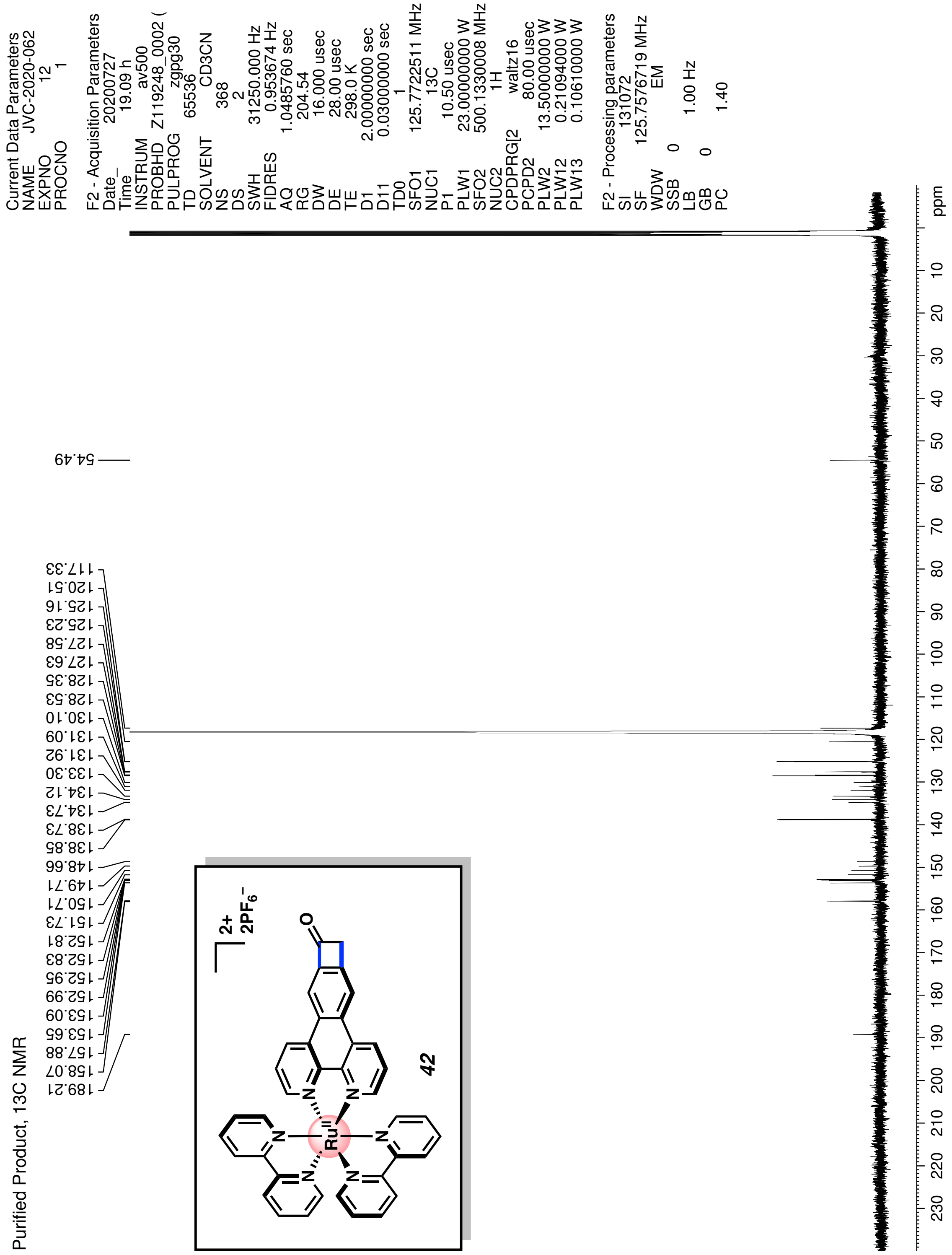

Supplementary Figure 61. ^{13}C NMR (125 MHz, CD_3CN) of 42.

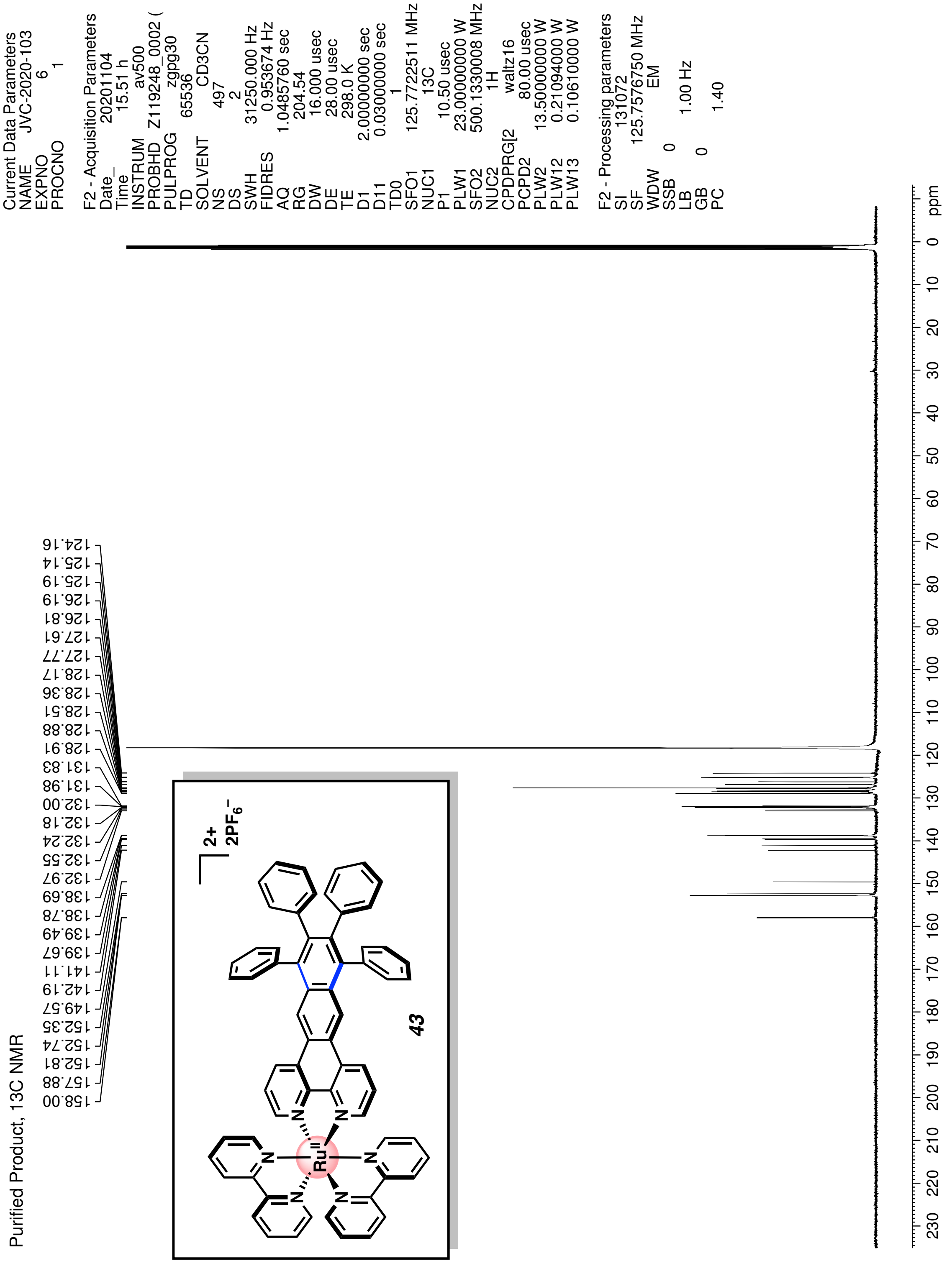

Supplementary Figure 62. ^{13}**C NMR (125 MHz, CD**$_3$**CN) of 43.**

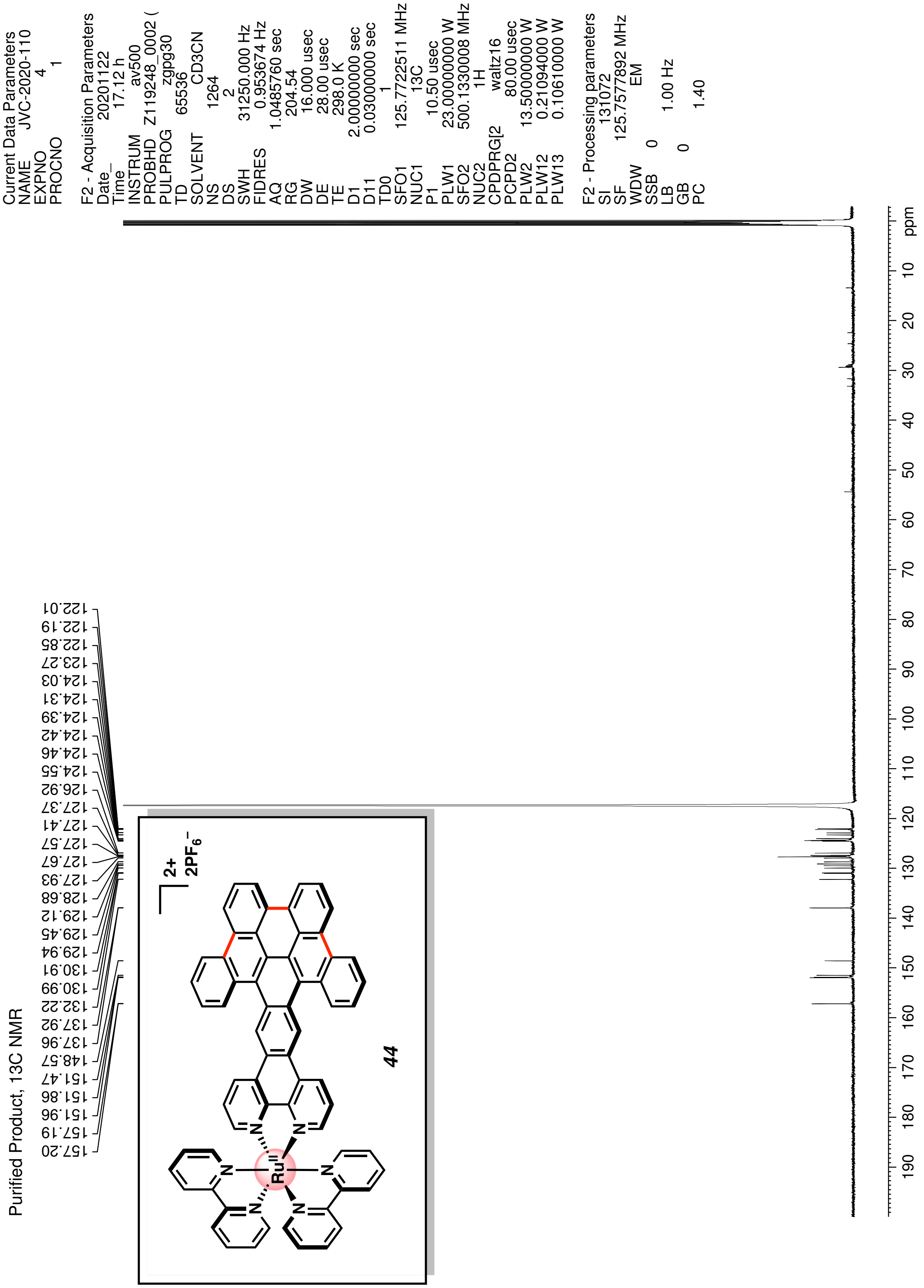

Supplementary Figure 63. ^{13}C NMR (125 MHz, CD_3CN) of 44.

Supplementary References

1. Young, M. C. et al. Spin state modulation of iron spin crossover complexes via hydrogen bonding self-assembly. *Chem. Commun.* **49**, 6331–6333 (2013).

2. Zucker, S. P. et al. Palladium-Catalyzed Directed Halogenation of Bipyridine *N*-Oxides. *J. Org. Chem.* **82**, 5616–5635 (2017).

3. Cheong, P. H.-Y. et al. Indolyne and Aryne Distortions and Nucleophilic Regioselectivites. *J. Am. Chem. Soc.* **132**, 1267–1269 (2010).

4. Suzuki, S., Itami, K. & Yamaguchi, J. Synthesis of Octaaryl Naphthalenes and Anthracenes with Different Substituents. *Angew. Chem. Int. Ed.* **56**, 15010–15013 (2017).

5. Breton, G. W. Selective Monoacetylation of Unsymmetrical Diols Catalyzed by Silica Gel Supported Sodium Hydrogen Sulfate. *J. Org. Chem.* **62**, 8952–8954 (1997).

6. Suzuki, K. et al. Reevaluation of absolute luminescence quantum yields of standard solutions using a spectrometer with an integrating sphere and a back-thinned CCD detector. *Phys. Chem. Chem. Phys.* **11**, 9850–9860 (2009).

7. Méndez-Hernández, D. D. et al. Building and testing correlations for the estimation of one-electron reduction potentials of a diverse set of organic molecules. *J. Phys. Org. Chem.* **28**, 320–328 (2015).

8. CYLview20; Legault, C. Y., Université de Sherbrooke, 2020 (http://www.cylview.org).
